# Evolutionary relationships, biogeography and morphological characters of *Glinus* (Molluginaceae), with special emphasis on the genus composition in Sub-Saharan Africa

**DOI:** 10.3897/phytokeys.173.60898

**Published:** 2021-02-22

**Authors:** Alexander P. Sukhorukov, Alexander Sennikov, Marie Claire Veranso-Libalah, Maria Kushunina, Maya V. Nilova, Roger Heath, Alison Heath, Yuri Mazei, Maxim A. Zaika

**Affiliations:** 1 Department of Higher Plants, Biological Faculty, Lomonosov Moscow State University, 119234, Moscow, Russia; 2 Laboratory Herbarium (TK), Tomsk State University, Lenin Ave. 36, 634050, Tomsk, Russia; 3 Botanical Museum, Finnish Museum of Natural History, P.O. Box 7, 00014 University of Helsinki, Finland; 4 Herbarium, Komarov Botanical Institute of Russian Academy of Sciences, Prof. Popov St. 2, 197376 St. Petersburg, Russia; 5 Institut für Molekulare Physiologie, Johannes Gutenberg–Universität Mainz, Germany; 6 Department of Plant Physiology, Biological Faculty, Lomonosov Moscow State University, 119234, Moscow, Russia; 7 University of Botswana, Plot 4775, Notwane Road, Gaborone, Botswana; 8 Royal Botanic Gardens, Kew, Richmond, TW9 3AE, United Kingdom; 9 Department of Hydrobiology, Biological Faculty, Lomonosov Moscow State University, 119234, Moscow, Russia

**Keywords:** Biogeography, *
Glinus
*, Molluginaceae, molecular phylogeny, Sub-Saharan Africa, taxonomic revision

## Abstract

*Glinus* is a small genus of Molluginaceae with 8–10 species mostly distributed in the tropics of the World. Its composition and evolutionary relationships were poorly studied. A new molecular phylogeny constructed here using nuclear (ITS) and chloroplast (*rbcL*, *trnK-matK*) markers confirmed the monophyly of the genus. Based on ITS analysis, the following well-supported lineages are present within *Glinus*: the *G. bainesii* lineage is recovered as sister to the remainder of the genus followed by *G. oppositifolius.* Three other clades are: *G. hirtus* with *G. orygioides*; *G. radiatus* and *G. lotoides*; the latter is represented by a sample from North America, and *G. zambesiacus* as sister to *G. setiflorus* + *G. lotoides* + *G. dictamnoides*. On the plastid gene tree, *G. bainesii* + *G. oppositifolius* form a sister clade to all other *Glinus* species. The next clade is formed by *G. hirtus* and *G. orygioides* followed by *G. radiatus* plus an American sample of *G. lotoides*. The next branch comprises *G. setiflorus* as sister to *G. zambesiacus* + *G. lotoides* + *G. dictamnoides*. *Glinus* seems to have originated from Africa around the Late Eocene or Early Miocene, with further radiations to Australia and the Americas during the Late Miocene or Late Pliocene. Compared with the previous limited character set used for the diagnostics, we have found ten new morphological and carpological traits distinguishing *Glinus* members. In both trees based on nuclear and plastid datasets, the major phylogenetic clades cannot be characterized by the peculiar morphological characters. Many shared character states leading to their contrasting pattern in the multivariate analysis model are interpreted as a high homoplasy in the phylogenetically distant species. We paid special attention to the composition of the genus in Sub-Saharan Africa, a region with the greatest species diversity. Our results provide new insight into the taxonomy of *Glinus* in this region. Glinus lotoides var. virens accepted in many previous works is a synonym of *G. dictamnoides* that is closely related to *G. lotoides* based on molecular analysis and morphological characters. The status of the American populations of *G. lotoides* needs further investigation due to different characters of the specimens from the Old and the New World. Many specimens previously identified as G. lotoides var. virens and as the intermediates *G. lotoides* × *G. oppositifolius* belong to *G. zambesiacus***sp. nov.** and *G. hirtus***comb. nov.** (≡ *Mollugo hirta*); the latter species is resurrected from synonymy after 200 years of unacceptance. In some African treatments, *G. hirtus* was known under the invalidly published name *G. dahomensis*. *Glinus zambesiacus* is distributed in the southern and eastern parts of tropical Africa, and *G. hirtus* previously assumed to be endemic to West Africa is indeed a species with a wide distribution across the tropical part of the continent. *Glinus microphyllus* previously accepted as endemic to West Tropical Africa together with other new synonyms (G. oppositifolius var. lanatus, *G. herniarioides*, *Wycliffea rotundifolia*) is considered here as G. oppositifolius var. keenanii**comb. nov.** (≡ Mollugo hirta var. keenanii), a variety found across the entire distribution of *G. oppositifolius* (Australia, Asia, and Africa). The presence of the American *G. radiatus* in Africa is not confirmed, and all records of this species belong to *G. hirtus*. The lectotypes of some names (*G. dictamnoides*, *G. herniarioides*, *Mollugo hirta*, *M. setiflora*, *Pharnaceum pentagynum*, *Wycliffea*) as well as a neotype of *G. trianthemoides* are designated. A new key to the identification of all *Glinus* species in Sub-Saharan Africa is provided. A checklist is given of all accepted species in this region (*G. bainesii*, *G. hirtus*, *G. lotoides*, *G. oppositifolius* s.l., *G. setiflorus*, and *G. zambesiacus*) with their nomenclature, morphological description and geographical distribution.

## Introduction

*Glinus* L. comprises six to ten species distributed in the warm regions of the world (Backer 1951; Endress and Bittrich 1993; Thulin et al. 2016). It includes annual and perennial herbs and rarely subshrubs covered with simple or stellate hairs, with exstipulate, lanceolate to obovate leaves arranged in false whorls of 3–5, several to many verticillate, sessile or pedicellate flowers, pentamerous perianth consisting of free segments that are green dorsally and white, pink or yellow ventrally, petaloid staminodes often present originating from the outer stamen whorl, 3–30 stamens and 3- or 5-valvate, strongly hydrochastic capsules with numerous small arillate seeds. Among Caryophyllales as well as Molluginaceae, *Glinus* is characterized by the remarkable seed aril divided into two parts: a white, usually well-visible hood covering the funiculus and a large ribbon-like appendage (Pitot 1965; Narayana and Lodha 1972; Endress and Bittrich 1993; Sukhorukov et al. 2018). Molecular phylogenetic analyses based on four species (*G. lotoides* L., *G. oppositifolius* (L.) Aug.DC., *G. radiatus* (Ruiz & Pav.) Rohrb. and *G. setiflorus* Forssk.) suggest the genus is monophyletic (Christin et al. 2011; Thulin et al. 2016).

Species delimitation within *Glinus* is usually based on the pubescence details (stellate vs. simple trichomes; presence of tiny prickles on stem and leaves), leaf dimensions and shape, number of flowers in the leaf axils, and number of stamens (e.g., Bogle 1970; Short 2002; Lu and Hartmann 2003; Vincent 2003). These character sets are sufficient for the delimitation of *G. lotoides* and *G. oppositifolius*, the most widespread species in the Old World (e.g., Hutchinson and Dalziel 1927; Backer 1951; Jeffrey 1961; Gonçalves 1978; Hargreaves 1995; Gilbert 2000; Short 2002; Retief and Meyer 2017). In some cases, seed size and ultrasculpture are useful tools for identification, e.g., in the characterization of *G. setiflorus*, *G. orygioides* F.Muell. and *G. bainesii* (Oliv.) Pax (Sukhorukov et al. 2018). Additional characters (pubescence density and pedicel length) were used for the infraspecific descriptions of the morphologically heterogeneous *G. lotoides* and *G. oppositifolius* (Gonçalves 1965, 1970, 1978). There was an attempt to divide *Glinus* into two subgeneric groups called *Euglinus* (≡ *Glinus*) and *Pseudoglinus* Endl. mainly based on stellate vs. non-stellate pubescence (Endlicher 1840), but this classification was never used.

The members of the genus are unevenly distributed across the tropics and subtropics of the world with most species present in Africa. In total, seven *Glinus* species are currently accepted in all parts of the continent (Klopper et al. 2006; APD 2019). *Glinus lotoides* is present throughout Africa (Jeffrey 1961; Maire 1962; Gonçalves 1978; APD 2019). *Glinus oppositifolius* possesses a similarly wide distribution, but is not reported from North Africa (Maire 1962; Boulos 1999; Hassan et al. 2005). Other species are considered to be restricted to smaller regions. *Glinus setiflorus* is widespread in E and NE Africa (Jeffrey 1961; Gilbert 1993a, 2000). *Glinus dahomensis* A.Chev. and *G. microphyllus* Hauman are considered to be local endemics to different territories of West Africa (Chevalier 1938; Hauman 1949; Lebrun and Stork 2003). Another African species, *G. bainesii* is restricted to Botswana, Mozambique, Zimbabwe and the eastern part of South Africa (Adamson 1961; Gonçalves 1978). A single adventive species *G. radiatus* (Ruiz & Pavon) Rohrb. from the Americas was referenced in some West African countries (Berhaut 1967, 1979; Lisowski 2009; Thiombiano et al. 2012; Schmidt et al. 2017). Additionally, it is postulated that *G. lotoides* and *G. oppositifolius* seem to freely hybridize in Africa with a large number of intermediates (Adamson 1961; Jeffrey 1961; Gonçalves 1970, 1978). Numerous specimens usually labelled as “*G. lotoides* × *G. oppositifolius*” are present in many herbaria. The reason for this identification is because of the less prominent stellate pubescence in these *Glinus* specimens compared to the typical densely pubescent specimens of *G. lotoides*. However, such hybrids were not reported from other parts of the Old World.

In a previous carpological study of all Molluginaceae (Sukhorukov et al. 2018), the disparity of many specimens belonging to the same *Glinus* species in the herbaria visited was noted. In fact, the limited number of diagnostic characters used and absence of detailed taxonomic treatment do not allow for an evaluation of the real diversity of *Glinus* in tropical Africa, the most species-rich region of the genus in the world. For this reason, a critical study of the taxonomy, morphology and distribution of the genus in Sub-Saharan Africa would be desirable. Therefore, the main aims of the present study are: (1) to conduct an expanded molecular phylogenetic analysis of *Glinus* worldwide, with further implications on its divergence and origin; (2) to deeply investigate the morphology and species distribution of *Glinus* in tropical Africa, the most species-rich region, and to provide precise diagnoses through an updated taxonomic treatment.

## Material and methods

### Phylogenetic analysis

#### Taxon sampling

In the molecular phylogenetic analysis, 149 accessions representing 31 species and 10 genera including outgroups were sampled. Except for the Indian *G. ononoides* where samples could not be amplified because of no recent collection, all other *Glinus* species were sampled. In total, nine *Glinus* species representing their entire distribution were sampled. A list of all samples used in this study is presented in Table 1.

**Table 1.** Voucher information and GenBank accession numbers for the species of Molluginaceae and outgroups included in the phylogenetic analysis (arranged in alphabetical order). The newly sequenced samples are highlighted in bold.

Species	Voucher	ITS	*rbcL*	*trnK-matK*
***Glinus bainesii* 8**	Botswana, Khwai River floodplain, Moremi Game Reserve, 21 Nov 2007, *A. Heath & R. Heath 1417* (K);	MW280260	MW275761	MW286109
***Glinus bainesii* 40**	Zimbabwe, Masvingo prov., Chiredzi, 31 May 1971, *S. Mari 1273* (LE);	MW280259	MW275762	MW286110
***Glinus dictamnoides* 18**	Yemen, Habban, 2 Mar 1997, *M. van Slageren & A. Al Gifri s.n.* (LE);	MW280251	MW275753	MW286101
***Glinus dictamnoides* 41**	India, [without exact location] 20 Feb 1955, *Baldev 7* (MW)	MW280250	MW275752	MW286100
***Glinus hirtus* 3**	Tanzania, Kagera region, Ngara, 6 Sep 1960, *R. Tanner 5126* (BR0000017454308)	MW280247	MW275760	MW286108
***Glinus hirtus* 6**	Malawi, Central Region, Nkhotakota distr., 13 May 1986, *I.H. Patel & R.B. Kwatha 3184* (BRLU0026262);	MW280249	MW275758	MW286106
***Glinus hirtus* 32**	Mali, nr Bamako, Niger river, Sotuba, 4 Jul 1973, *D. N’Golo 1291* (WAG0319754)	MW280248	MW275759	MW286107
***Glinus lotoides* 9**	Mali, Koulikoro Region, Koulikoro, 6 May 1993, *R. Ehrlich 463* (B100048300)	–	MW275747	MW286095
***Glinus lotoides* 19**	Eritrea, Northern Red Sea region, Wekiro, 31 Jan 1960, *D.J. Greathead 139* (BM)	MW280254	MW275746	MW286094
***Glinus lotoides* 20**	Niger, Touaret, 10 Dec 1965, *G. Popov 138* (BM)	MW280252	MW275748	MW286096
***Glinus lotoides* 37**	Australia, Queensland, 4 Sep 1984, *J.R. Clarkson 5494* (LE)	MW280253	MW275749	MW286097
*Glinus* “*lotoides*” (American sample)	USA, California, *Ertter 8854 & 8859* (NY)	KT907409	FN824412	FN825692
*Glinus oppositifolius*	Taiwan, *Huang & Huang 14175* (NY)	KT907366	FN824415	FN825695
*Glinus oppositifolius*	Australia, *Barbidge 5949* (ANH)	–	FN824415	FN825696
*Glinus oppositifolius*	China, Xisha Islands, *Anonymous s.n.* (without herbarium acronym)	MH768251	–	–
*Glinus oppositifolius*	China, Xisha Islands, *Anonymous s.n.* (without herbarium acronym)	MH768252	–	–
*Glinus oppositifolius*	Tanzania, *Balslev 630* (NY)	–	FN824414	FN825694
***Glinus oppositifolius* 34**	India, Goa State, Dec 2018, *A. Sukhorukov s.n.* (MW)	MW280255	MW275755	MW286103
**Glinus oppositifolius var. keenanii 4**	Zambia, Western Province, Bulozi plain near Mongu, Oct 1993, *M.G. Bingham 9743* (WAG0334359)	MW280256	MW275754	MW286102
***Glinus orygioides* 35**	Australia, Lake Eyre, 29 Sep 2007, *M.J. Thorpe & al. 119* (LE)	MW280263	MW275763	MW286111
*Glinus radiatus*	USA, *Thomas 114677* (NY)	KT907410	FN824417	FN825697
***Glinus radiatus* 25**	Brazil, Bahia, 2 Nov 1988, *R.M. Harley & al. 25863* (W)	MW280258	MW275756	MW286104
*Glinus setiflorus*	Kenya, *Burney & al. T46* (NY)	KT907367	N824418	FN825698
***Glinus setiflorus* 38**	Kenya, Kitui county, Ukasi Dam, 3 Aug 2006, *L. Festo & Q. Luke 2813* (LE);	MW280257	MW275757	MW286105
***Glinus zambesiacus* 29**	Zambia, Luapula prov., Samfya, 21 Apr 1989, *G. Pope & al. 2194* (BR0000018269093)	MW280261	MW275751	MW286099
***Glinus zambesiacus* 42**	Zambia, Southern prov., Namwala, 9 Jan 1957, *E.A. Robinson 2101* (BR0000018269581)	MW280262	MW275750	MW286098
**outgroup**				
*Adenogramma glomerata*	South Africa, *Ogburn 142* (BRU)	KT907379	FN824408	FN825689
*Adenogramma teretifolia*	South Africa, *Ogburn 156* (BRU)	KT907381	FN824410	FN825691
*Hypertelis cerviana_1* (sub *Mollugo cerviana*)	Namibia, *Potgieter 225* (K)	KT907402	FN824427	FN825707
*Hypertelis cerviana_2* (sub *Mollugo cerviana*)	Burkina Faso, *Ataholo 1809* (FR)	KT907392	FN824435	FN825715
*Hypertelis fragilis* (sub *Mollugo fragilis*)	Angola, *Gossweiler 6* (K)	KT907397	FN824441	FN825723
*Hypertelis spergulacea*	Namibia, *Thulin & Larsson 11962* (UPS)	KT907407	-	-
*Hypertelis spergulacea*	Namibia, *Giess et al. 5366* (K)	-	FN824420	FN825700
*Mollugo flavescens*	Ecuador, Galapagos Islands, *Wheeler et al. 17* (NY)	KT907417	FN824438	FN825720
*Mollugo floriana*	Ecuador, Galapagos Islands, *Eliasson 741* (K)	KT907413	FN824440	FN825722
*Mollugo snodgrassii*	Galapagos Islands, *Howell 9450* (NY)	KT907411	FN824457	FN825738
*Mollugo tenella*	Namibia, *Merxmüller & Giess 3316* (NY)	KT907414	FN824458	FN825739
*Mollugo verticillata*_1	no voucher information	MH768259	MH767665	MH767966
*Mollugo verticillata*_2	Bolivia, *Nee 37372* (G)	KT907368	FN824474	FN825743
*Paramollugo angustifolia* (sub *Mollugo angustifolia*)	Somalia, *Thulin et al. 7606* (UPS)	KT907356	FN824422	FN825702
*Paramollugo decandra* (sub *Mollugo decandra*)	Madagascar, *Croat 30852* (K)	KT907358	FN824437	FN825718
*Paramollugo nudicaulis* (sub *Mollugo nudicaulis*)	India, *Devi s.n.* (CANB)	KT907365	FN824451	FN825733
*Pharnaceum detonsum*	South Africa, *Fries 764* (NY)	KT907416	FN824463	FN825745
*Pharnaceum incanum*	South Africa, *Ogburn 148* (BRU)	KT907387	FN824466	FN825748
*Pharnaceum lanuginosum*	South Africa, *Ogburn 161* (BRU)	KT907385	FN824469	FN825752
*Polpoda capensis*	South Africa, *Acocks 17405* (CANB)	KT907384	FN824470	FN825753
*Psammotropha obovata*	South Africa, *Hilliard & Burtt 7045* (K)	KT907383	FN824471	FN825754
*Psammotropha quadrangularis*	South Africa, *Ogburn 160* (BRU)	KT907382	FN824472	FN825755
*Suessenguthiella scleranthoides*	South Africa, *Acocks 18950* (K)	KT907390	FN824473	FN825756
*Trigastrotheca molluginea* (sub *Mollugo molluginea*)	Australia, *Telford 11746* (CANB)	KT907408	FN824443	FN825725
*Trigastrotheca pentaphylla* (sub *Mollugo pentaphylla*)	India, *n/a*	KT907377	FN824455	FN825737

#### DNA extraction, amplification and sequencing

5–10 mg of dried herbaria leaf samples was used to isolate DNA using the CTAB protocol (Doyle and Doyle 1987). One nuclear (the nuclear ribosomal internal transcribed spacer, nrITS) and two plastid markers (the coding gene rbcL and the region encompassing *trnK* introns and *matK* coding genes, *trnK-matK*) were selected for phylogenetic analysis. The primers ITS4 and ITS5 (White et al. 1990) were used to amplify the ITS region. The *rbcL* and *matK*-*trnK* primer sequences were taken from Christin et al. (2011). Due to the fact that the samples were taken from herbaria and had a rather long storage term, various combinations of the forward primers trnK-matK_For A, C, G, and H and the reverse primers trnK-matK_Rev C, D, F and I were used. For *rbcL*, two external rbcL_4_For and rbcL_1353 and two internal primers rbcL_629_For and rbcL_760_Rev were used (Christin et al. 2011).

PCR reactions for all primers were performed in a total volume of 25 μl, using 5 μl DNA (10 ng/μl), 1 μl of each primer, 0.5 μl Encyclo polymerase (Evrogen, Russia), 0.5 μl 50× dNTP, 5 μl 10× Encyclo buffers and 14.5 μl mQ.

PCR amplification of nrITS primers was performed under the following conditions: initial denaturation for 3 min at 94 °C; 37 cycles of 1 min denaturation at 94 °C, 30 s annealing at 51 °C, 150 s extension at 72 °C, and final extension of 10 min at 72 °C (Thulin et al. 2016). PCR amplification for *rbcL* was performed under the following conditions: initial denaturation for 10 min at 94 °C; 34 cycles of 30 s denaturation at 94 °C, 30 s annealing at 48 °C, 150 s extension at 72 °C, and final extension of 10 min at 72 °C. The following PCR program was used for *matK-trnK* primers: initial denaturation for 10 min at 94 °C; 34 cycles of 30 s denaturation at 94 °C, 30 s annealing at 51 °C, 2 min extension at 72 °C, and final extension of 10 min at 72 °C.

PCR products were cleaned with Cleanup Mini BC023S Kit (Evrogen, Russia). Sanger sequencing was carried at Evrogen JSC (Moscow, Russia) using the same primers as in the PCR.

#### Phylogenetic inference and molecular dating

Sequences were aligned using MUSCLE v.3.5 (Edgar 2004) and the alignment was adjusted manually using PhyDe (version 0.9971; Müller et al. 2010). Three separate analyses were performed for the nuclear and plastid DNA datasets using maximum parsimony (MP), Bayesian inference (BI) and maximum likelihood (ML). Due to a conflict between *G. zambesiacus* and *G. setiflorus*, all subsequent analyses were conducted using the separate datasets. Models of nucleotide substitution were chosen according to the Akaike information criterion using jModelTestv.2.1.7 (Guindon and Gascuel 2003; Darriba et al. 2012) for each gene separately. The best-fit substitution model for both the nuclear and plastid datasets was GTR + G. For the ML analyses, we used RAxML Version 8 (Stamatakis 2014). Bootstrap analyses were conducted with 2500 replicates for ML. Parsimony analyses were conducted in PAUP* 4.0a162 (Swofford 2002) with the following settings: all characters have equal weight, MaxTrees set to 1000 (auto increased by 1000), TBR branch swapping and with 20000 jackknife (JK) replicates to calculate node support. Final trees were edited in TreeGraph ver. 2.14.0 (Stöver and Müller 2010).

Divergence times for *Glinus* were estimated using a Bayesian uncorrelated lognormal relaxed clock under a birth–death speciation process (Gernhard 2008) for the nuclear and plastid datasets separately. We selected a normal distribution for the secondary calibration with a standard deviation of 8.5, equivalent to the 95% HPD estimate of Yao et al. (2019) for the crown of Molluginaceae. Four independent MCMC analyses were run, each of 20 million generations sampling every 2000. The analyses were run using BEAST 2.4.5 (Bouckaert et al. 2014) on the CIPRES Science Gateway 3.3 (https://www.phylo.org; Miller et al. 2010). Output log files were analyzed using TRACER 1.6 (Rambaut et al. 2014) to assess convergence and ESS of all parameters. As “burn-in”, 10% of samples were removed prior to combining the independent runs using LOGCOMBINER 2.4.5 (Bouckaert et al. 2014). The MCC tree was generated using TREEANNOTATOR 2.4.5 (Bouckaert et al. 2014).

### Biogeographical analysis

Geographical distributions of all species were compiled from herbarium specimens and field work (see section “Field and herbarium studies” below). Due to the wide distribution of some species (*G. hirtus*, *G. lotoides*, *G. oppositifolius*, *G. radiatus*), the biogeographical analysis is based on the continents and not the floristic provinces. Four geographical areas were identified: A – Africa including Madagascar; B – Asia; C – Australia, and D – America (including Galapagos Islands). The BI gene trees were pruned to remove all duplicate accessions and *G. dictamnoides* using the drop.tip function in the package *ape* (Paradis et al. 2004). The nuclear and plastid gene trees used for the analyses had 32 accessions each corresponding to 31 species. Two accessions of *G. lotoides* were included representing the Old World and the American populations. The coded geographic data is available in Table 2.

**Table 2.** The coding of the geographical areas of *Glinus* species and outgroup (all – Molluginaceae).

Taxon	Geographical areas
*G. bainesii*	A
*G. hirtus* (known as *G. dahomensis* nom. inval.)	A
*G. lotoides*	A, B, C
*G. lotoides* (American sample)	D
*G. oppositifolius*	A, B, C
*G. orygioides*	C
*G. radiatus*	D
*G. setiflorus*	A
*G. zambesiacus*	A
**Outgroup**	
*Adenogramma glomerata*	A
*Adenogramma teretifolia*	A
*Hypertelis cerviana*	A & B
*Hypertelis fragilis*	A
*Hypertelis spergulacea*	A
*Mollugo flavescens*	D
*Mollugo floriana*	D
*Mollugo snodgrassii*	D
*Mollugo tenella*	A
*Mollugo verticillata*	D
*Paramollugo angustifolia*	A
*Paramollugo decandra*	A
*Paramollugo nudicaulis*	A & B
*Polpoda capensis*	A
*Pharnaceum detonsum*	A
*Pharnaceum incanum*	A
*Pharnaceum lanuginosum*	A
*Psammotropha obovata*	A
*Psammotropha quadrangularis*	A
*Suessenguthiella scleranthoides*	A
*Trigastrotheca molluginea*	C
*Trigastrotheca pentaphylla*	B

Ancestral range estimation (ARE) was conducted using the R package “BioGeoBEARS” (Matzke 2013, 2014). Out of the six models explored in this study, the DEC+J model was the best fit based on the AIC and likelihood ratio test (LRT) results (see Table 3). The analyses were unconstrained (without possible dispersal routes or ancestral areas assumed a priori). We allowed the inferred ancestor to occupy a maximum of three areas, corresponding to the maximum number of areas occupied by any extant species.

Based on the likelihood and AIC values, the best fit model was the DEC +J model for both nuclear and plastid datasets (Table 3). Both datasets had the same results and only varied at the divergence times.

**Table 3.** Results of the biogeographical analysis using BioGeoBEARS.

Nuclear and plastid datasets							
	LnL	numparams	d	e	j	AIC	AIC_wt
DEC	-51.29	2	0.0083	1.00E-12	0	106.6	0.19
DEC+J	-48.91	3	0.0063	1.00E-12	0.023	103.8	0.74
DIVALIKE	-53.03	2	0.0099	1.00E-12	0	110.1	0.033
DIVALIKE+J	-51.72	3	0.0079	1.00E-12	0.016	109.4	0.044
BAYAREALIKE	-66.51	2	0.012	0.031	0	137	4.60E-08
BAYAREALIKE+J	-55.51	3	0.005	1.00E-07	0.035	117	0.001

### Multivariate analysis

The same species set of *Glinus* as in the molecular phylogenetic analysis were included in the character matrix. The varieties of *G. oppositifolius* (var. glomeratus and var. keenanii) that deviate in some states of the studied characters as well as *G. ononoides* and *G. sessiliflorus* were not included. The multivariate analysis aims to test whether the morphological and carpological character subdivision corresponds with the phylogenetic reconstructions. In our previous papers, multivariate analysis provided a good support for the non-stochastic distribution of the characters in major clades of the entire Molluginaceae (Sukhorukov et al. 2018) and the genus *Microtea*, Microteaceae (Sukhorukov et al. 2019).

Different *Glinus* species were classified by group average linkage algorithm of cluster analysis constructed on a Gower similarity matrix (Gower 1971) based on seventeen characters including general morphology (life history, pubescence, leaves) and reproductive traits. This approach recognizes the species grouping based on similar characters, but does not provide a true phylogenetic context. The reliability of grouping was assessed at the level p<0.05 using SIMPROF algorithm (Clarke 1993; Clarke and Warwick 2001). Calculations were performed using PRIMER 6.1.6 statistical software (Clarke and Gorley 2006).

### Carpological study

The seeds were investigated using scanning electron microscopy (SEM) and anatomically. The hard seed coat does not require any special preparation prior to SEM due to absence of any trichomes on its surface. The seed colliculae if present are the thickenings of the outer walls of the testa cells. After sputter coating the material with gold-palladium, the SEM observations were made with a JSM–6380 microscope (JEOL Ltd., Japan) in the Laboratory of Electron Microscopy of Moscow State University. The anatomical cross-sections of seeds were prepared using a rotary microtome Microm HM 355S (Thermo Fisher Scientific, USA). Before sectioning, the seeds were soaked in water:alcohol:glycerin (1:1:1) solution, dehydrated in an ethanol dilution series and embedded in Technovit 7100 resin (Heraeus Kulzer, Germany). The cross-sections were observed using a Nikon Eclipse Ci microscope and photographed with a Nikon DS-Vi1 camera (Nikon Corporation, Japan) at the Department of Higher Plants (Moscow State University).

The list of samples used for the SEM is provided in Table 4.

**Table 4.** List of species and vouchers used in the carpological analysis. The specimens used for both anatomy and SEM analyses are marked with an asterisk (*) after the herbarium acronym. The samples of the widely distributed species *G. lotoides* and *G. oppositifolius* originated from different regions of the World.

Species	Origin of the material
*G. bainesii*	Botswana, Ngamiland, 100 m S of Samudupe newbridge, 19 Feb 2008, *B. Farrington et al. 486* (K)*;
*G. dictamnoides*	Kenya, South province, Magadi road, 3000 ft, 8 Aug 1943, *P.R.O. Bally 2679* (G)*; Sri Lanka, Central province, Sep 1974, *D.B. Sumithraarachchk 496* (K);
*G. hirtus*	South Africa, Northern Cape, Henkries, 30 Nov 1897, *B. Schlechter s.n*. (G); Malawi, Zomba, 2500–3500 ft, [without date] *A. Whyte s.n*. (G); Senegal, [without exact location and date] *Perrottet 373* (G); Somalia, Ganaane to Marro Mogale Umberto I, Mar 1893, *D. Riva 736* (G); Nigeria, Niger prov., Bida distr., 1 Mar 1968, *B.O. Daramola & A. Binuyo 61930* (K)*; Burkina Faso, Kompienga, 40 km E of Tindangou, 2 May 2003, *L. Sanou & M. van Slageren 1352* (K); DR Congo, Inkisi river, 17 Oct 2007, *Nsimundele 2060* (BR0000000530230);
*G. lotoides*	Indonesia, Java, 1908, *H. Winkler s.n*. (BM); Madagascar [Boeny Region], Madirovalo, 4 Oct 1930, *M. Decary 8194* (G); Israel, Upper Jordan valley, 19 Jul 1943, *T. Kushnir s.n*. (HUJ)*; Australia, Northern territory, Simpson desert, Jul 1968, *A.E. Orchard 759* (K); Nigeria, Kainji Lake, 21 Jul 1973, *R. Linnavuori s.n*. (H1209977)*; Niger, 100 km S of Agadez, 23 Nov 1985, *C. Pase 3149* (K);
*G. ononoides*	[India, leg. D. Freyn] (G00808773)*
*G. oppositifolius*	Philippines, [no location and year] *Blanco 385* (LE); India [state of Assam], Cachar [distr.], Mar 1873, *R.L. Keenan s.n*. (K000641798), type of Mollugo hirta var. keenanii; [Vietnam] nr Hanoi, Jun 1891, *B. Balansa 4613* (K000641793), as *G. herniarioides*; DR Congo, Boma, 1 Aug 1913, *Bequaert 536* (BR000000895538) (var. keenanii); Tanzania, Kyimbila distr. N of Lake Nyasa, 1915, *A. Stolz s.n*. (K)*; [DR Congo] Mateba, 14 Dec 1912, *R. Verschueren 203* (BR000000895505); Sierra Leone, nr Kasanko, 13 May 1951, *P. Adams 225* (K); Tanzania, [Tanga Region] Handeni distr., Horogwe to Handeni, 18 Nov 1955, *E. Milne-Redhead & P. Taylor 7337* (K) (var. keenanii); Zambia, Mongu distr., Kande Lake, 8 miles NE of Mongu, 11 Nov 1959, *R.B. Drummond & A.J.Cookson 6335* (E); Angola, Cuando-Cubango prov., Longa, 1360 m, 17 Mar 1960, *E.J. Mendes 3155* (M) (var. glomeratus); DR Congo, 26 km NW of Lubumbashi, Lupoto, 2 Mar 1961, *A. Schmitz 7129* (P05307011) (var. glomeratus); DR Congo, Katanga prov., Kundelungu Plateau, Lualaba, 1700 m, Mar 1969, *S. Lisowski & al. 4053* (BR0000018268348) (var. glomeratus); India, Goa State, Dec 2018, A. *Sukhorukov s.n*. (MW)*;
*G. orygioides*	Australia, Eyre Lake, 29 Sep 2007, *M.J. Thorpe et al. 119* (K)*;
*G. radiatus*	Cuba, 1824, *Anonymous s.n*. (BR); Dominican Rep., Santo Domingo, Jul 1912, *M. Fuertes 1859* (E); Mexico, Temascaltepec distr., Feb 1933, *G.B. Hinton 4065* (BR)*; Venezuela, Estado Guárico, 8 May 2010, *A. Fernández et al. 27692* (G);
*G. sessiliflorus*	Australia, Northern Territory, Annaburroo, 8 Sep 2002, *B.J. Lepschi & J.R. Connors 4854* (W)*;
*G. setiflorus*	Ethiopia, Ogaden, nr Mardere, 2 Dec 1953, *G. Popov 1143* (K)*; Somalia, El Buur distr., 27 km S of Guri Ceel, 6 Jun 1988, *P. Kuchar 17836* (K);
*G. zambesiacus*	Zambia, Barotseland [Western province], Mongu, 6 Feb 1966, *Robinson 6780* (M)*; DR Congo, Katanga prov., Pweto, 18 May 1971, *F. Malaisse 6767* (BR0000018269567).

### Choosing the territory for taxonomic study

Due to the fact that *Glinus* has the highest taxonomic diversity in the tropics of Africa but is represented only by a single widespread species (*G. lotoides*) in North Africa (Maire 1962; Boulos 1999) and a poorly known local endemic in Luxor (*G. runkewitzii* Täckh. & Boulos), which is closely related to *G. lotoides* (Täckholm and Boulos 1972), we thus exclude the northern part of the continent (Algeria, Egypt, Libya, Morocco and Tunisia) from our investigations. The territory under study corresponds to the geographical concept designed for the framework for the investigation of biodiversity and conservation of Sub-Saharan plants (Gautier et al. 2006; Klopper et al. 2006).

### Field and herbarium studies

Field investigations were conducted by the first author (APS) in many regions of Namibia in 2017–2018, Tanzania (Arusha and Kilimanjaro Regions, Unguja Island) in 2019 and 2020, in surroundings of Victoria Falls (Zimbabwe), Livingstone (Zambia) and Kasane (Botswana) in 2020. Alison and Roger Heath, Honorary Research Associates at RBG Kew, UK and Research Affiliates at University of Botswana, carried out a 20-year botanical research project in the Okavango Delta and surrounding areas of the Kalahari Desert and published interim results of the floristic investigations as a book (Heath and Heath 2009). The study of the herbarium material was undertaken by the first author (APS) at B (images), BACH (images), BM, BOL (images), BR, BRLU, COI (images), E, FI (images), FR, FT (images), G, H, HUJ, K, L (incl. U & WAG), LE, LICS (images), M, MHA, MSB, MW, P, PE (images), PRE (images), RO, STU (images), W, WU, Z (images). Only a part of the images seen from BOL, LISC and PRE were exactly identified and cited in the present article; other specimens from these herbaria requiring more detailed analysis were not cited here. No *Glinus* specimens were seen from Equatorial Guinea, Lesotho and West Sahara.

Distribution maps are based on the specimens cited in the text and were prepared using SimpleMappr online tool (http://www.simplemappr.net).

### Nomenclature

Protologues of each plant name involved were examined for valid publication, legitimacy, and other nomenclatural issues in agreement with the International Code of Nomenclature for algae, fungi, and plants (Turland et al. 2018). As far as possible, original material was traced and type specimens were cited or designated here.

## Results

### Molecular phylogeny and dating

**Figure 1.**
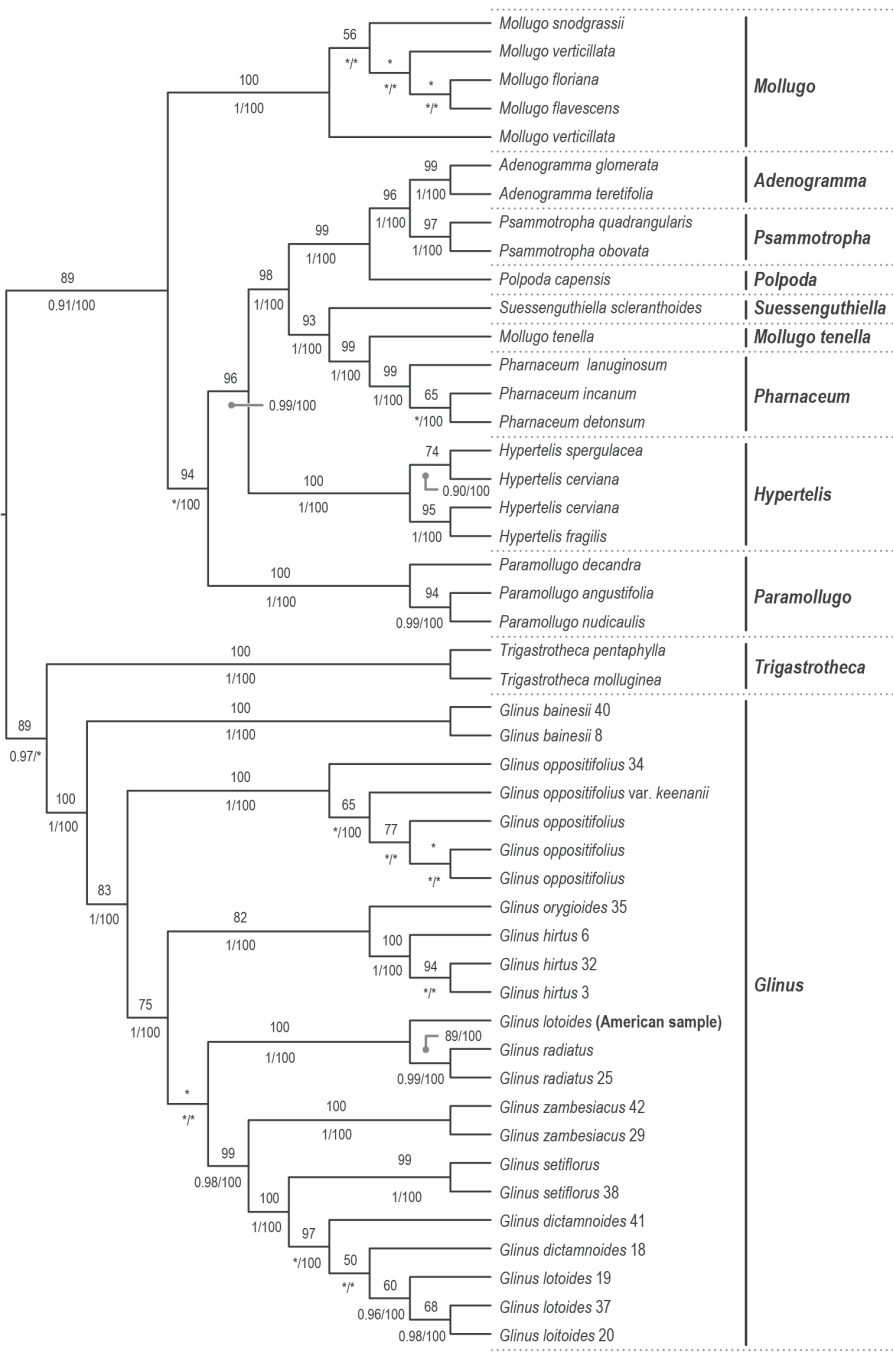
Maximum likelihood phylogenetic cladogram of *Glinus* derived from the nrITS. Values above branches refer to bootstrap values resulting from the ML analysis (only values ≥50). Values below branches refer to posterior probabilities resulting from Bayesian inference (only values ≥0.95) and bootstrap values resulting from the parsimony analysis (only values ≥50). An asterisk (*) denotes a branch unsupported by either bootstrap values or posterior probability.

**Figure 2.**
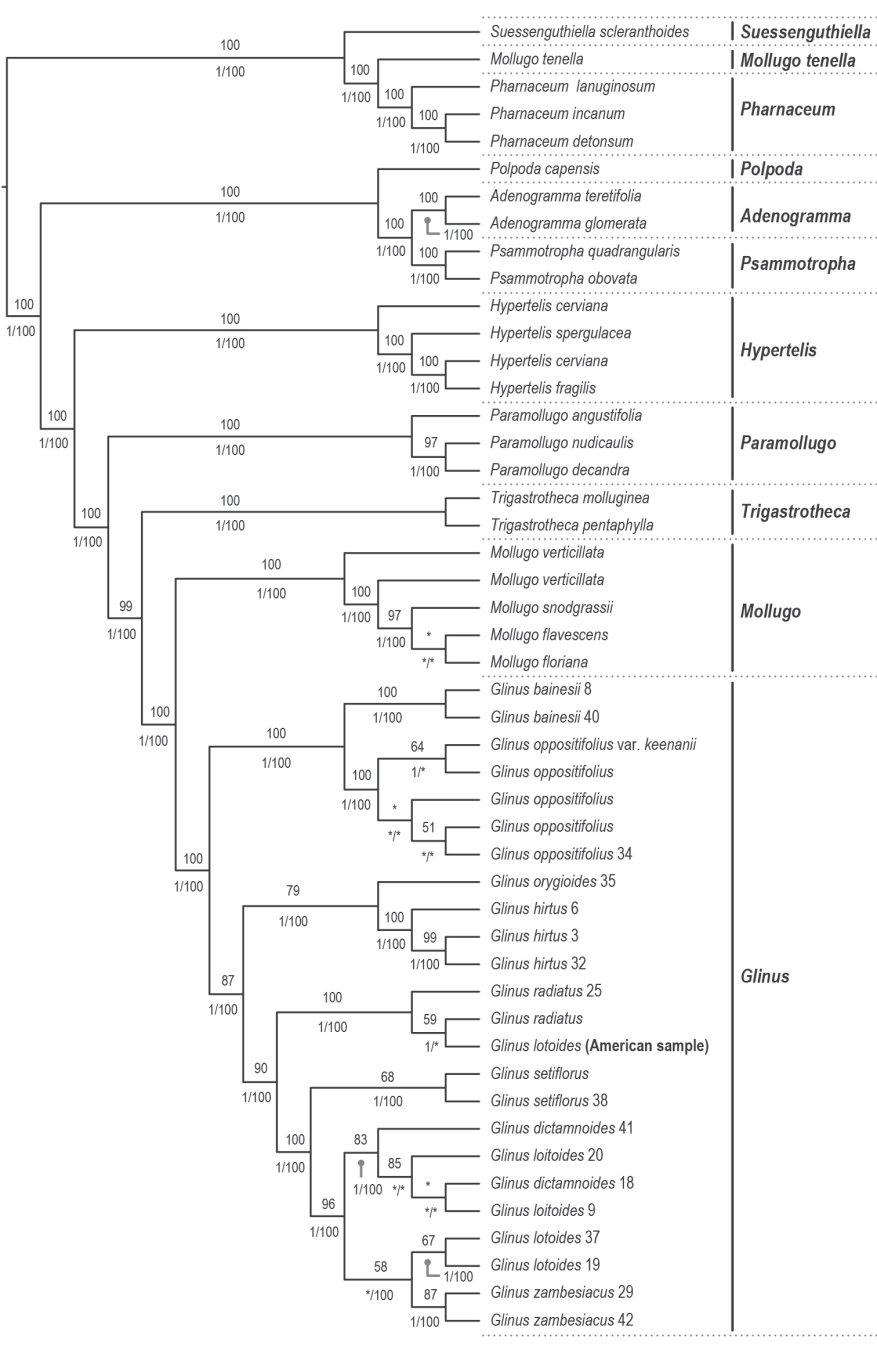
Maximum likelihood phylogenetic cladogram of *Glinus* derived from the combined plastid matrix (rbcL, trnK-matK). Values above branches refer to bootstrap values resulting from the ML analysis (only values ≥50). Values below branches refer to posterior probabilities resulting from Bayesian inference (only values ≥0.95) and bootstrap values resulting from the parsimony analysis (only values ≥50). An asterisk (*) denotes a branch unsupported by either bootstrap values or posterior probability.

The combined plastid dataset of *rbcL* and *matK*-*trnK* comprises 4009 aligned bp and 48 accessions while the nrITS dataset has 760 aligned bp and 47 accessions. The ML and BI analyses revealed identical topologies, although slightly different from the MP (see Figs 1, 2). In all three analyses, *Glinus* is resolved as monophyletic. In the nuclear gene trees, *Glinus* is sister to *Trigastrotheca* F.Muell. (Fig. 1; BSL 89; PP 0.97) while in the plastid gene trees it is recovered as sister to *Mollugo* (Fig. 2; BSL 100; PP 1). In the parsimony analyses, *Glinus* is sister to *Mollugo* in both the plastid (BSP 100) and nuclear (BSP 100) gene trees.

In the plastid gene trees, *G. bainesii* + *G. oppositifolius* form a clade sister to all other *Glinus* species. In this clade, *G. hirtus* + *G. orygioides* are in turn sister to the remaining species. *Glinus radiatus* plus the northern American *G. lotoides* sample are sister to a clade composed of *G. setiflorus*, *G. dictamnoides*, *G. zambesiacus* and *G. lotoides* (Fig. 2).

In the nuclear gene tree, *G. bainesii* is recovered as sister to the remaining species. The *G. oppositifolius* lineage is recovered as sister to a well-supported clade of (1) *G. hirtus* + *G. orygioides*; (2) *G. radiatus* and the North American *G. lotoides* and (3) *G. zambesiacus* as sister to *G. setiflorus* + *G. lotoides* + *G. dictamnoides* (Fig. 1).

The plastid and nuclear gene trees had a conflict regarding the position of *G. zambesiacus* and *G. setiflorus*. In the plastid gene tree, *G. setiflorus* is well-supported (BSL 100; BSP 100; PP 1) as sister to a clade composed of *G. zambesiacus*, *G. dictamnoides* and *G. lotoides* whereas in the nuclear gene tree *G. zambesiacus* is well-supported (BSL 99; BSP 100; PP 0.98) as sister to a clade composed of *G. setiflorus*, *G. dictamnoides* and *G. lotoides* (Figs 1, 2).

**Figure 3.**
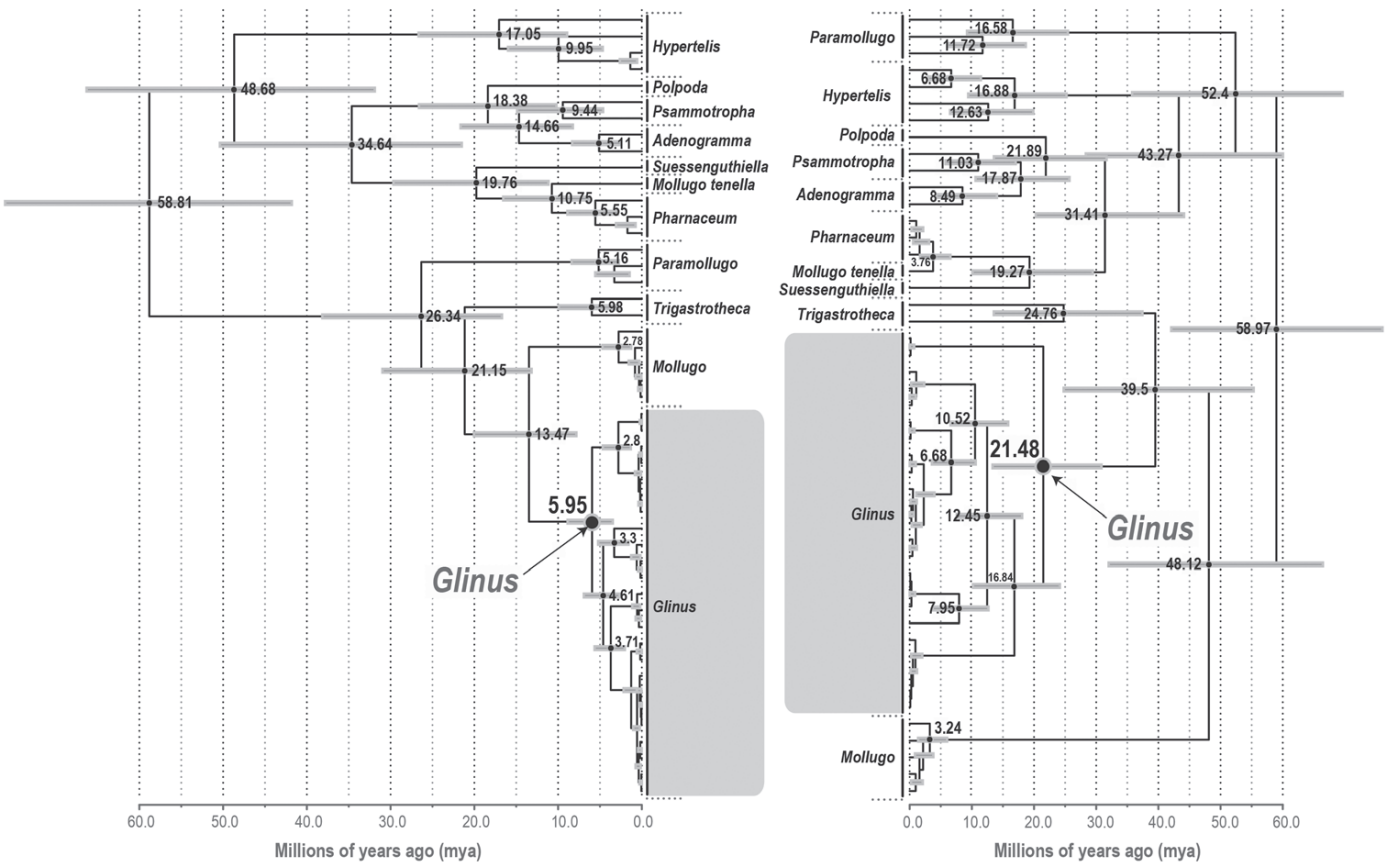
Maximum clade credibility (MCC) tree of *Glinus* obtained from the BEAST analysis calibrated using secondary calibrations (see Methods). Mean divergence times (values at some nodes) are shown with their 95% highest posterior density (HPD: grey bars). To the left is the plastid gene tree and to the right the nuclear gene tree, respectively.

Except for the crown node of Molluginaceae, the other nodes show very different ages for both the nuclear and plastid gene trees. The nuclear gene tree had much older node ages compared to the plastid gene tree (Fig. 3). Molluginaceae started to diversify during the Late Paleocene at ~58.97 mya (95% HPD 42.07–76.03 mya) or ~58.81 mya (95% HPD 41.8–76.03 mya) based on the nuclear and plastid trees, respectively. The diversification of *Glinus* started around the Late Eocene ~39.5 mya (stem age, 95% HPD: 13.33–30.85) or around the Middle Miocene ~13.47 mya (stem age, 95% HPD: 7.82–20.06 mya) for the nuclear and plastid trees, respectively (Fig. 3).

### Biogeographical analysis

**Figure 4.**
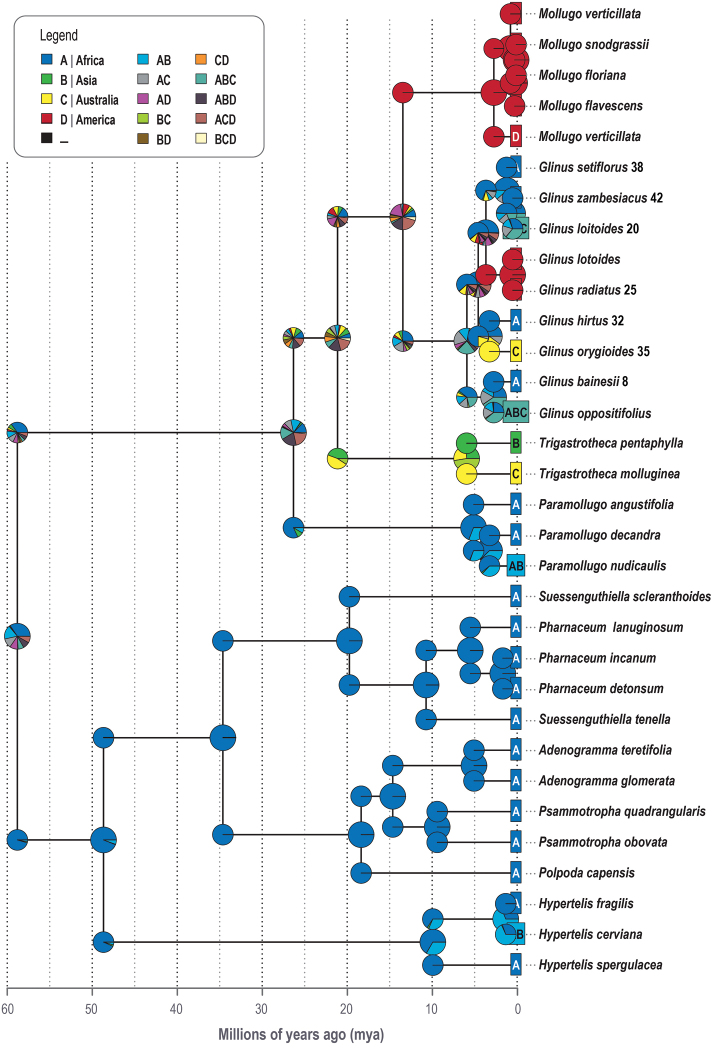
Ancestral range estimation (ARE) based on the reduced MCC tree (“BioGeoBEARS” DEC+J on *Glinus* unconstrained ancstates: global optim, three areas max. anagenetic dispersal rate, d = 0.0063; extinction rate, e = 0; cladogenetic dispersal rate, j = 0.023; likelihood ratio test, LnL = -48.91). Coding of biogeographical areas as shown in the legend. Coding of species areas is given in coloured squares left of each species. Pie charts represent the ancestral area probability inferred for each node.

In the biogeographical analysis, the nuclear and plastid gene trees based on the reduced data showed the same topology but only varied with the divergence times. The nuclear and plastid trees based on the reduced data showed the same topology, as such similar biogeographic results only differing by node ages. The ancestral area of *Glinus* is uncertain (see Fig. 4 ACD: p = 0.21; AD: p = 0.18; ABD: p = 0.16; A: p = 0.10). The ancestral area of the crown node of *Glinus* remains uncertain (Fig. 4; ABC: p = 0.23; A: p = 0.19; AC: p = 0.19; AC: p = 0.19; AB: p = 0.14) even though it seems to be connected to Africa. There have been two shifts from Africa, one to Australia for *G. orygioides* and the other to America for *G. radiatus* and the American *G. lotoides* clade.

### Diagnostic characters revisited

We coded 17 characters (14 morphological and 3 anatomical characters) used for the multivariate analyses (see Table 5). Out of the 17 characters coded, 10 are used for species delimitation for the first time (characters 4, 7, 8, 10, 11, 13, 14, 15, 16 and 17; see Table 5). The following characters and states were coded.

**Table 5.** Coded matrix for multivariate analysis of *Glinus* species. The varieties of the species that deviate in characters compared with the type variety were not included in the Table. To be consistent with the molecular phylogeny, the same species set was used for the multivariate analysis (*G. ononoides* and *G. sessiliflorus* were included in the Table, but they are absent in both molecular and multivariate analyses).

Taxon/character states	1	2	3	4	5	6	7	8	9	10	11	12	13	14	15	16	17
*G. bainesii*	1	0	1	0	1	0	1	1	1	2	2	0	2	0	2	1	1
*G. dictamnoides*	0	1	0	1	0	0	0	0	1	1	2	1	2	0	1	1	1
*G. hirtus*	0	1	0	0	0	1	1	0	0	1	1	0	1	0	1	1	1
*G. lotoides* (Old World)	0	1	0	1	0	0	0	1	1	1	2	1	2	0	1	1	1
*G. lotoides* (New World)	0	1	0	0	0	0	0	0	0	0	0	0	0	0	1	0	0
*G. ononoides*	0	1	1	0	0	1	1	0	0	1	1	0	0	0	0	0	0
*G. oppositifolius*	0	0	0	0	1	0	1	0	0	1	0	0	1	0	1	1	1
*G. orygioides*	2	0	1	0	1	0	0	1	1	1	2	0	2	1	2	1	1
*G. radiatus*	0	1	0	0	0	1	1	0	0	0	0	0	0	0	0	0	0
*G. sessiliflorus*	0	0	0	0	0	0	1	0	0	1	0	0	1	0	1	1	1
*G. setiflorus*	0	1	0	1	0	0	0	1	1	1	1	1	2	1	1	1	1
*G. zambesiacus*	0	1	0	0	1	0	1	1	1	2	2	0	1	0	1	1	1

1. Life form: annual (0); annual to short-lived perennial herb (1); subshrub (2).
2. Pubescence: soft simple hairs (0); stellate hairs (1).
3. Presence of prickles on the stem, leaves and perianth: absent or scattered and unnoticeable (0); present and distinguishable (1).
4. Leaf veins: adaxially not recessed and abaxially not prominent (0); adaxially recessed and abaxially prominent (1).
5. Presence of pedicel: flowers sessile or subsessile (pedicel up to 5.0 mm long) (0); pedicel more than 5.0 mm long (1).
6. Flower clusters: usually up to 7–10 flowers (0); more than 10 flowers (1).

Note. Within the state (0), *G. setiflorus* usually has 1–2 flowers in the cluster, and other species – from 4 to 10 flowers. However, this quantitative difference is not strictly expressed in *G. bainesii*, *G. oppositifolius* and *G. zambesiacus*.

**Figure 5.**
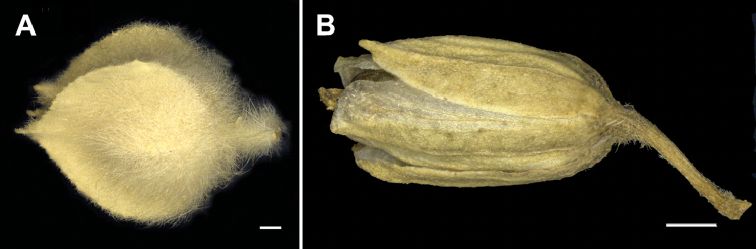
Closed anthocarp of *Glinus* species **A***G. setiflorus***B***G. zambesiacus*. Scale bar: 1 mm.

1. Flower buds and closed anthocarp (Fig. 5): ovoid or roundish (0); cylindrical (1).
2. Length of perianth segments in fruiting: up to 5.5 mm (0); more than 5.5 mm (1).
3. Number of stamens: 2–7 (0); 10 or more (1).
4. Length of anthers: up to 0.6 mm (0); 0.6–1.0 mm (1); (1.0)1.2–1.8 mm (2).
5. Length of stylodia and stigmas (or style + stigmas): up to 0.6 mm (0); (0.5)0.6–1.0 mm (1); more than 1.0 mm (2).
6. Stigmas (or style with stigmas): 3 (0); usually 5 (1).

Note. A character “Presence of the style” (united part of the stylodia) is not included in the matrix, because it can be variable within a species. The short style is mentioned in *G. lotoides* (Hofmann 1973). In *G. hirtus*, *G. lotoides*, *G. oppositifolius*, *G. setiflorus* and *G. zambesiacus* the style, if present, is equal in the length to the stigmas.

**Figure 6.**
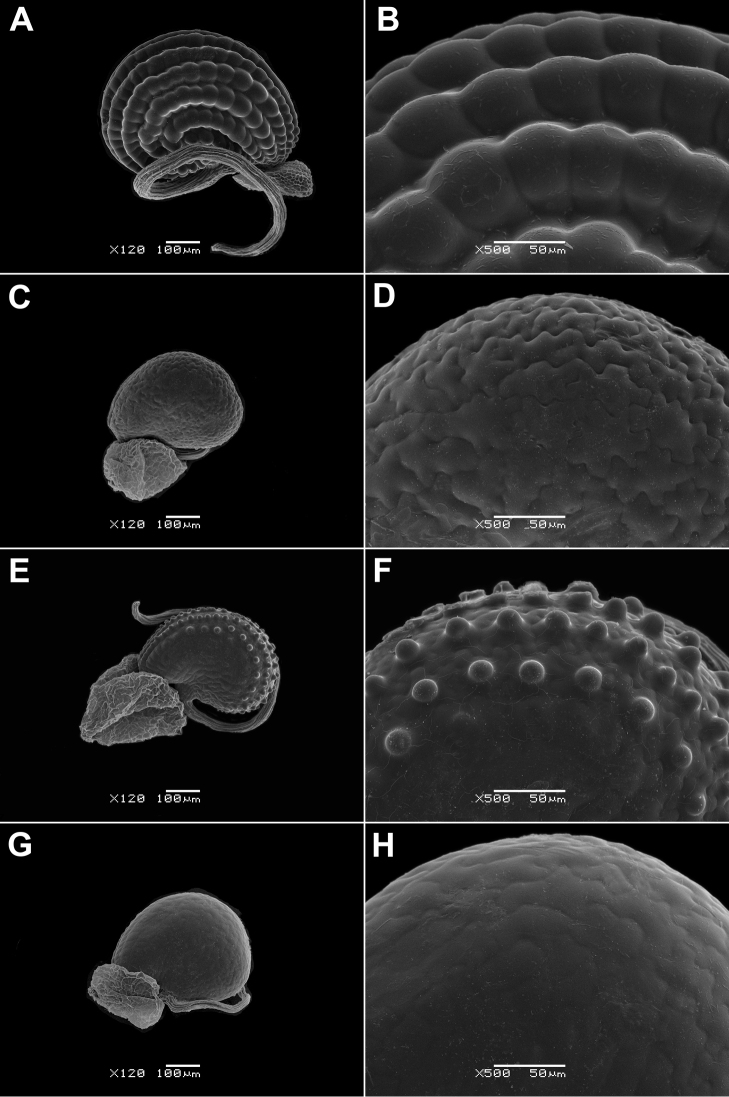
Seeds of *Glinus* species **A, B***G. bainesii* (Botswana, *B. Farrington et al. 486*, K) **C, D***G. hirtus*, a form with smooth seeds (Senegal, *Perrottet 373*, G) **E, F***G. hirtus*, colliculate seeds (DR Congo, *Nsimundele 2060*, BR) **G, H***G. ononoides* (India, *D. Freyn* s.n., G). Magnification: 120× (**A, C, E, G**); 500× (**B, D, F, H**).

**Figure 7.**
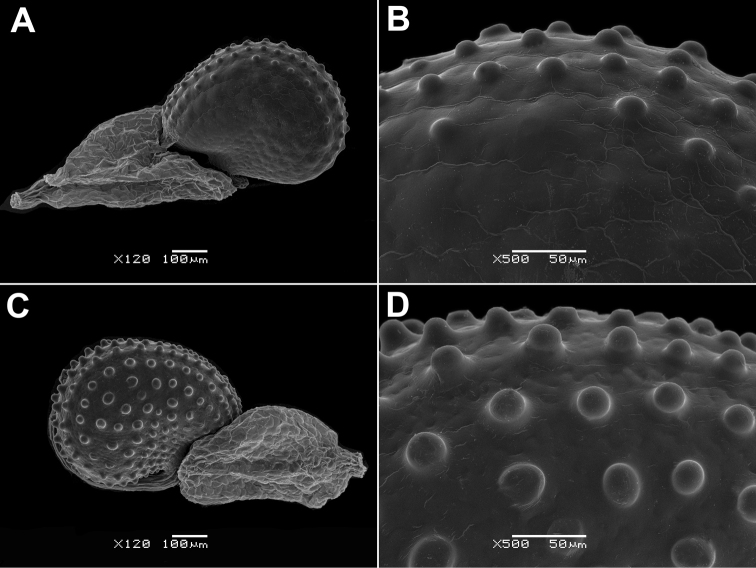
Seeds of *Glinus* species **A, B***G. lotoides* (Israel, *T. Kushnir* s.n., HUJ) **C, D***G. dictamnoides* (Kenya, *P.R.O. Bally 2679*, G). Magnification: 120× (**A, C**); 500× (**B, D**).

**Figure 8.**
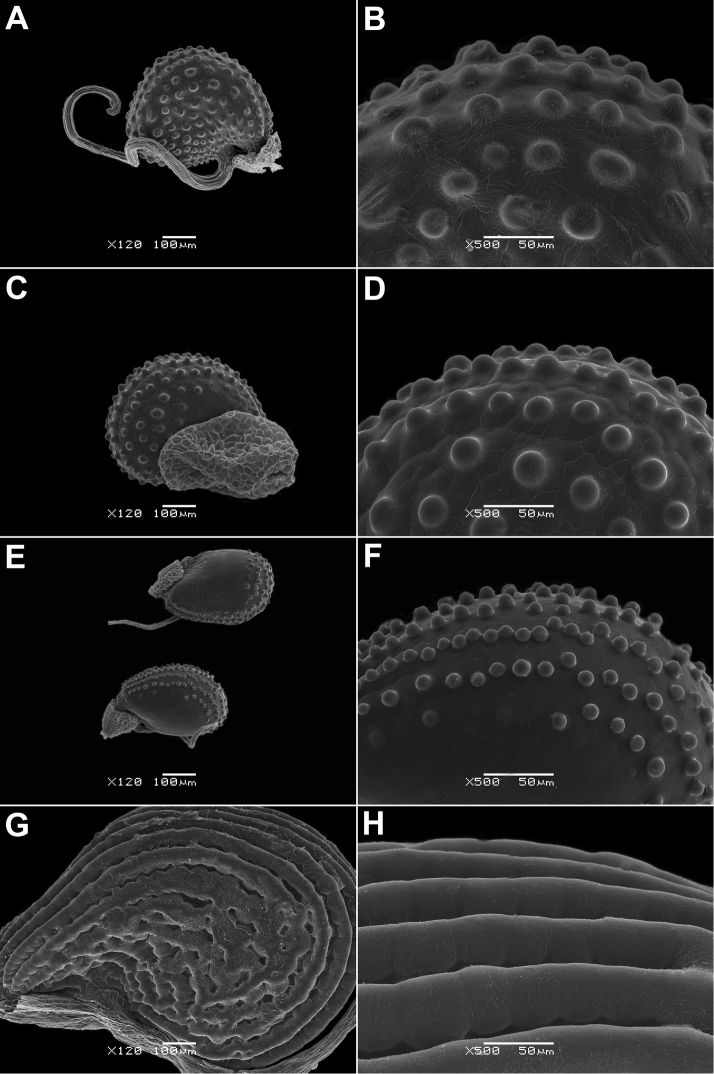
Seeds of *Glinus* species **A, B***G. oppositifolius* (Zambia, *R.B. Drummond & A.J.Cookson 6335*, E) **C, D**G. oppositifolius var. glomeratus (Angola, *E.J. Mendes 3155*, M) **E, F**G. oppositifolius var. keenanii (DR Congo, *R. Verschueren 203*, BR) **G, H***G. orygioides* (Australia, *M.J. Thorpe et al. 119*, K). Magnification: 120× (**A, C, E, G**); 500× (**B, D, F, H**).

**Figure 9.**
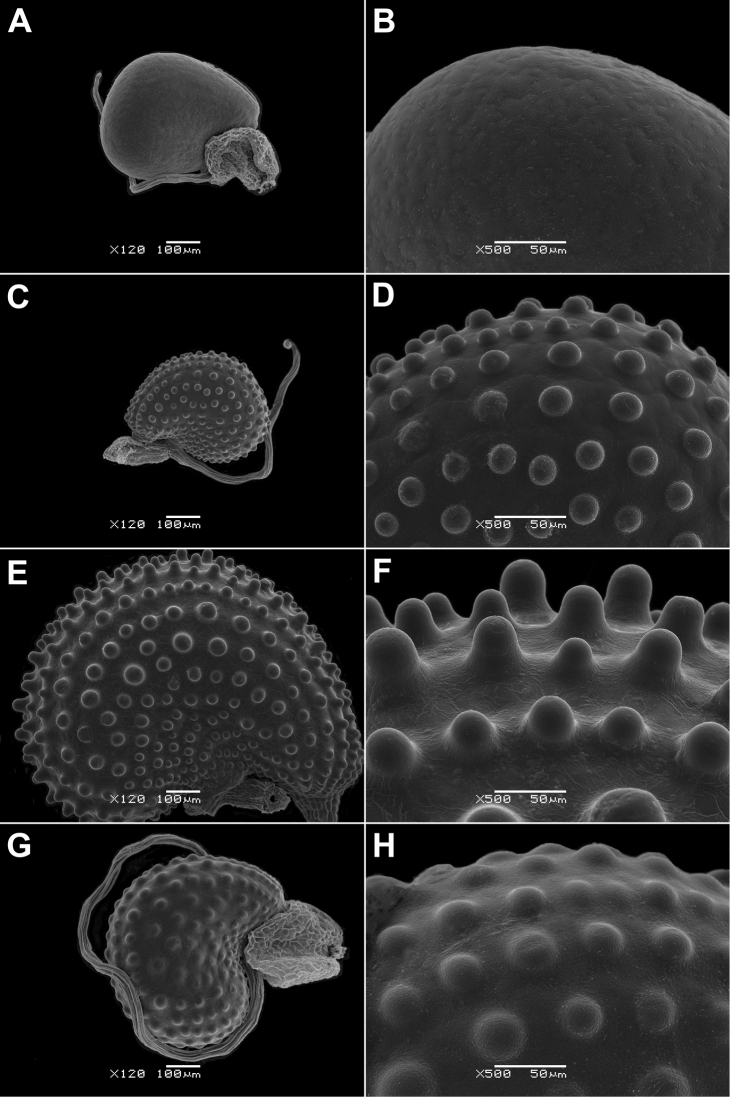
Seeds of *Glinus* species **A, B***G. radiatus* (Venezuela, *A. Fernández et al. 27692*, G) **C, D***G. sessiliflorus* (Australia, *B.J. Lepschi & J.R. Connors 4854*, W) **E, F***G. setiflorus* (Ethiopia, *G. Popov 1143*, K) **G, H***G. zambesiacus* (Zambia, *Robinson 6780*, M). Magnification: 120× (**A, C, E, G**); 500× (**B, D, F, H**).

1. Seed colour: yellow-brown (0); red, brown or reddish-brown (1); dark red or almost black (2).
2. Seed length: 0.35–0.60 mm (0); 0.7–0.9 mm (1).
3. Seed ultrasculpture (Fig. 6–9): seeds smooth or with barely noticeable colliculae (0); seeds without ridges, only with colliculae (1); seeds with concentric ridges and colliculae (2) (Figs 6A, B, 8G, H).

Note. Two seed types were observed in *G. hirtus*: seeds with smooth surface found in several specimens only (Fig. 6C, D) and more common colliculate ones (Fig. 6E, F). The colliculate ultrasculpture is the most common type in almost all species and their varieties (Figs 6E, F, 7A–D, 8A–F, 9C–H). All *Glinus radiatus* samples have a smooth seed surface (9A, B), as well as those of *G. ononoides* (Fig. 6G, H) and some specimens of *G. hirtus* (Fig. 6C, D).

**Figure 10.**
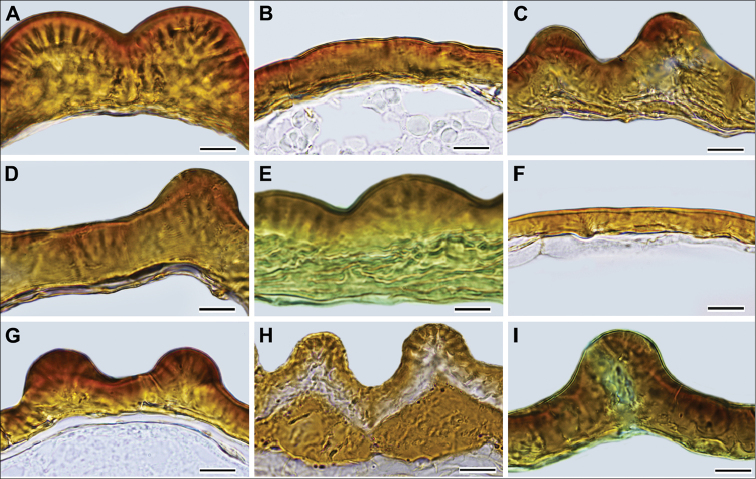
Seed coat cross-sections **A***G. bainesii***B***G. hirtus***C***G. lotoides***D***G. oppositifolius***E***G. orygioides***F***G. radiatus***G***G. sessiliflorus***H***G. setiflorus***I***G. zambesiacus*. Scale bar: 10 µm. *Glinus dictamnoides* has the same seed coat structure as *G. lotoides* and is not illustrated here. Origin of the material is provided in the Table 4 and is designated with an asterisk (*).

1. Stalactites in the testa (Fig. 10): not well-visible (0); prominent (1).
2. Thickness of the seed-coat testa: thin (up to 8 μm) (0); thick (10–30 μm, the thickest area is collicula) (1).

Note. Due to the presence of the colliculae making the seed coat more robust in these areas, the thickness of the seed coat was measured between them.

### Multivariate analysis

**Figure 11.**
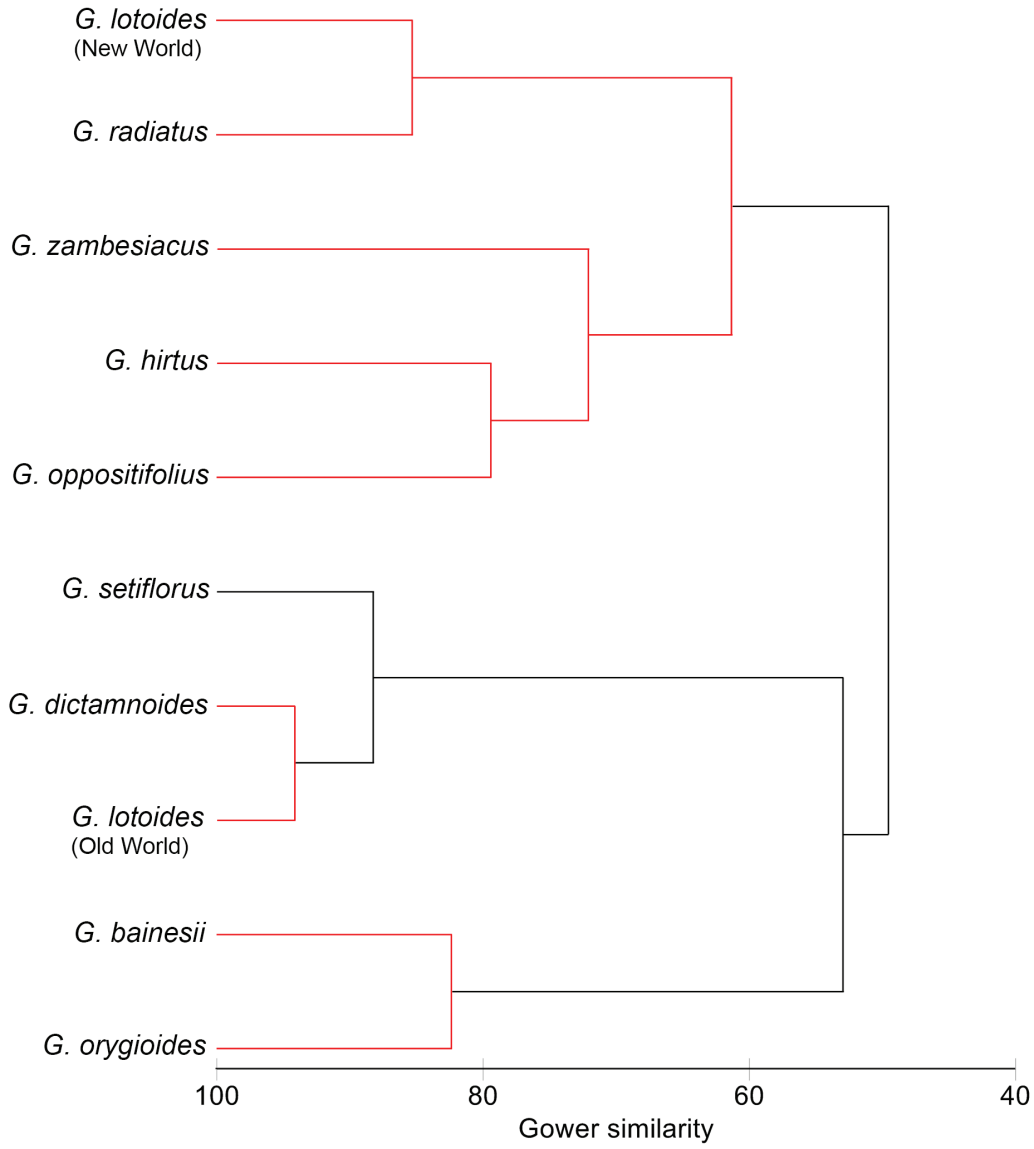
Classification of *Glinus* species by group average linkage algorithm of cluster analysis based on 17 characters. Black branches connect significantly (P < 0.05) different groups, red branches – insignificantly different groups.

The data of the multivariate analysis were evaluated using the matrix of the characters and their states provided in the Table 5. The results of cluster analysis of all characters suggest the existence of four significantly different groups within *Glinus*, these branches are highlighted in black (Fig. 11): (1) *G. bainesii* + *G. orygioides* (2) *G. setiflorus* (3) *G. lotoides* (Old World) + *G. dictamnoides*, (4) *G. radiatus* / *G. lotoides* (New World) + *G. zambesiacus* + *G. hirtus* / *G. oppositifolius*. The groups are significantly (p<0.05) distinct on different levels of Gower’s index.

The first group, *G. bainesii* and *G. orygioides*, share 13 characters states, and two of them (3:1, 15:2) are unique and not known in other *Glinus* species. *Glinus lotoides* from the Old World, *G. dictamnoides* and *G. setiflorus* (groups 2 and 3) show morphological similarity based on 15 characters (1:0, 2:1, 3:0, 4:1, 5:0, 6:0, 7:0, 8:1, 9:1, 10:1, 12:1; 13:2, 15:1; 16:1; 17:1). The highest number of the same character states (16 out of 17) is detected in the third group *G. lotoides* (specimens from the Old World) – *G. dictamnoides*. The fourth group unites the species with different character states, and only two of them (1:0 and 3:0) are the same for each species. Within this group, 13 character states are shared between *G. radiatus* and the American populations of *G. lotoides*, and between *G. hirtus* and *G. oppositifolius*. *Glinus zambesiacus* shares 10 character states (1:0, 3:0, 4:0, 7:1, 12:0, 13:1, 14:0, 15:1, 16:1, 17:1) with both *G. hirtus* and *G. oppositifolius*.

## Discussion

### Morphological interpretation of the phylogenetic results

Monophyly of *Glinus* is not surprising because all species share the same unique trait (presence of seed aril) not encountered in other genera of Molluginaceae. *Mollugo* s.str. was suggested to be closely related to *Glinus* after the first molecular studies (Christin et al. 2011; Thulin et al. 2016), but this is only partially supported by our results. The merging of *Glinus* into *Mollugo* s.l. previously undertaken by several authors (e.g., Bentham in Bentham and Mueller 1866; Oliver 1871; Trimen 1894; Clifton 2003) cannot be accepted, even though species of both *Mollugo* and *Glinus* share similar morphological and carpological characters, e.g., whorled leaves, leafy inflorescences, multi-seeded capsules, colliculate seeds with a relatively thick seed coat with stalactites. The carpological differences between the related genera were determined by Sukhorukov et al. (2018) based on an extended seed analysis.

The major clades of *Glinus* do not possess distinct morphological characters. None of the peculiar character states provided in Table 5 are known in *G. bainesii* and *G. oppositifolius*, the earlier diverging lineages of *Glinus*. Nevertheless, both species share 9 out of 17 character states (2:0, 4:0, 5:1, 6:0, 7:1, 12:0, 14:0, 16:1, 17:1). The longitudinally ridged seeds of *G. bainesii* are absent in *G. oppositifolius* and almost all other species except in the phylogenetically distant *G. orygioides*. Seeds with concentric ridges and colliculae (character state 15:2) are rather common in many *Mollugo* s.str. (Sukhorukov and Kushunina 2017).

The close relationship between the Australian *G. orygioides* and *G. hirtus* is unexpected from a morphological point of view. These species share only five character states (4:0, 10:1, 12:0, 16:1, 17:1). Compared with other *Glinus* species, *G. orygioides* differs by being a subshrub, while *G. hirtus* has no peculiar character states. Surprisingly, *G. hirtus* shares eleven character states (mostly gross morphological) with the unrelated *G. radiatus*. From the six states distinguishing these two taxa, only one (seed colour) is visible to the naked eye; however, some *G. hirtus* specimens have yellow seeds (13:0), a usual character state in *G. radiatus*. The other five character states are micromorphological (length of anthers and stylodia, seed ultrasculpture, presence of stalactites in the testa and its thickness). This similarity in the gross morphology has caused the misidentification of both species.

The results of the molecular analysis support the polyphyly of *G. lotoides*; a sample from the New World forms a clade together with *G. radiatus*. The specimens of American *G.* “*lotoides*” seen by us have yellow or bright brown seeds, like *G. radiatus* (not dark red or almost black as in *G. lotoides* s.str.: state 13:2 in the Table 5), and differ from it by colliculate seed sculpture (e.g., Thieret 1966) and larger perianth segments (Christy 1998). We provisionally accept only one native species in the Americas (*G. radiatus* s.l.). The close relationship between the Old World *G. lotoides* specimens and *G. setiflorus* is supported by many identical character states (Table 5; Figs 7A–D, 10C, H), and they both share nine states with *G. zambesiacus*. In the absence of a well-resolved relationship between *G. lotoides* and *G. dictamnoides* in the plastid and nuclear gene trees, we suggest that *G. dictamnoides* be treated as a synonym to *G. lotoides*, as was proposed in the earlier studies (e.g. Backer 1951; Zohary 1966; Hassan et al. 2005).

Due to the discordance between the gene trees and multivariate analysis of morphological characters, we cannot propose any infrageneric groups within *Glinus*. We assume that character states shared between phylogenetically distant taxa should be interpreted as homoplasies. Similarly, the former genus subdivision proposed by Endlicher (1840) and based on the pubescence details is also not supported.

### Biogeography

Both the plastid and nuclear gene trees suggest that *Glinus* started to diversify during the Neogene. Even though our results do not indicate a clear origin of *Glinus*, it seems to be connected to Africa (Fig. 4). Origin and adaptation to Neogene aridification in Africa has also been reported in many other plant lineages such as *Acridocarpus* Guill. & Perr., Malphigiaceae (Davis et al. 2002), *Coccinia* Wight & Arn., Cucurbitaceae (Holstein and Renner 2011), *Guibourtia*, Fabaceae (Tosso et al. 2017), *Manilkara* Adans., Sapotaceae (Armstrong et al. 2014) and the tribe Melastomateae, Melastomataceae (Veranso-Libalah et al. 2018). The Australian *Glinus orygioides* and the American *G. radiatus* group probably originated during the Late Miocene and Pliocene based on the plastid and nuclear gene trees, respectively. Long-distance dispersal might be the most appropriate explanation for migration of the species to Australia and America during the Neogene.

### Extant geographical distribution

Two species from the basal clade(s) – *G. oppositifolius* and *G. bainesii* – prefer different climates. *Glinus bainesii* is well adapted to the hot semi-arid climate [climate classification used here is according to Köppen (1936), with additions by Geiger (1961)]. *Glinus oppositifolius* is more frequently found in regions with tropical rainforest and savanna climates. In the regions with hot semi-arid or desert climates, it clearly prefers the habitats near water sources (e.g., river banks). Another widely distributed species, *G. lotoides*, as well as East African *G. setiflorus*, are drought-adapted species and avoid territories with tropical rainforest and monsoon climates.

**Figure 12.**
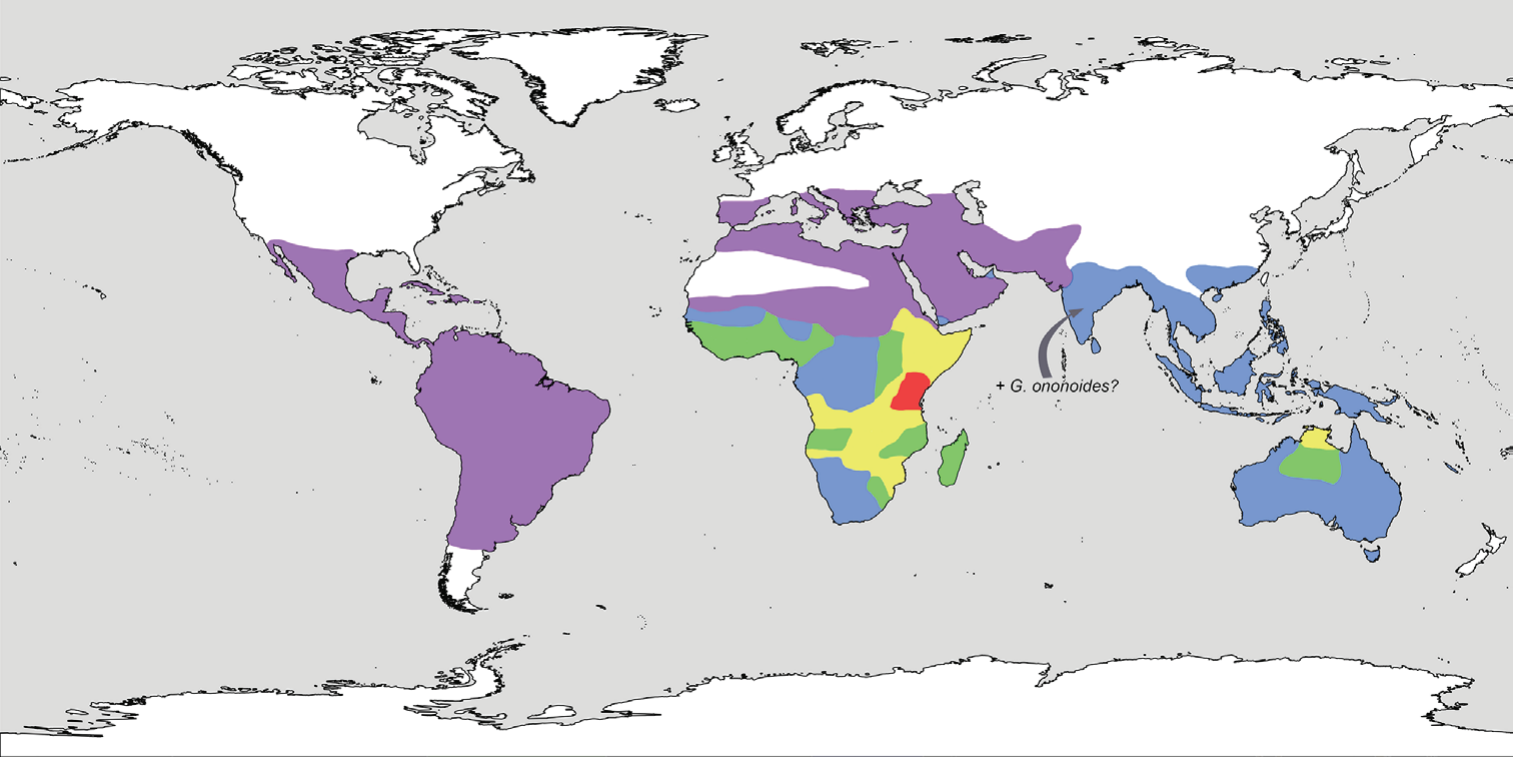
The number of *Glinus* species around the world. Areas coloured in mauve – one species, blue – two species, green – three species, yellow – four species, red – five species. Area boundaries are approximated. *G. ononoides* is a poorly known Indian taxon that needs to be studied further.

Altogether, we accept 9–10 species in (sub)tropical parts of the World. These can thrive in different habitats (riversides, deserts, stone outcrops, sandy coastal areas) and sometimes are noxious weeds, especially in the tropics with a humid climate. The species number is unevenly distributed around the World (Fig. 12). Four species occur in Australia (*G. lotoides*, *G. oppositifolius*, *G. sessiliflorus* and *G. orygioides*, endemic to N & C Australia: Short 2002, 2011), with the northern regions being the most species-rich. The Americas are reported to have two species: *G. lotoides*, considered to be an alien from the Old World, and *G. radiatus* (Grayum and Koutnik 1982; Boetsch 2002; Vincent 2003; Vigosa Mercado 2015). However, the presence of *G. lotoides* in the New World is doubtful. Further taxonomic studies are needed to decide whether the carpological and chorological data support the acceptance of the second (presumably native) *Glinus* species in North America. The species number in Asia is two or three: poorly known Indian *G. ononoides* with only two old collections seen by us (G! K!), *G. lotoides* and *G. oppositifolius* (e.g., Backer 1951; Hedge and Lamond 1975; Heller and Heyn 1994; Lu and Hartmann 2003; Townsend 2016; Byalt and Korshunov 2020) with synonyms or insufficiently studied taxa described from Asia (see Taxonomic section below). Only one species (*G. lotoides*) is present in S Europe (Paradis 1993; Tutin et al. 1993) as well as in North Africa (Maire 1962; Boulos 1999). On the contrary, the Sub-Saharan area incorporates two to six species, depending on the regions (Fig. 12). The most species-rich region (5 spp.) is East Africa (Kenya and Tanzania). A large region with a tropical savanna climate is the second richest region with four species.

### Possible mode of species dispersal

All *Glinus* species have hydrochastic capsules which open when triggered by rain drops (ombrohydrochory). This seems to be a somatic response of the plants to the climates characterized by alternating dry and wet periods. It is also known in many members of the Aizoaceae (Parolin 2006; Kurzweil and Burgoyne 2009) from areas with hot desert and semi-arid climates. In light of the presumable African origin of *Glinus*, such disseminative adaptation allows for a rapid seed dispersal during the rainy season.

In all species of the genus, the dispersal unit is a seed. The rains enable rapid dehiscence of the capsules and further dispersal of the seeds with surface water runoff. Additionally, the dry seeds due to their tiny size can easily roll over the substrate (Sulakshana and Raju 2018). However, it is unlikely that the seeds can move long distances in this manner, and, at least in *G. lotoides*, they remain viable only for several months (Bhatia 1987; Teshome and Feyissa 2015). We suggest that epizoochory may play a significant role in the dissemination, whereby moist substrate particles with seeds attached may inadvertently be carried by animals or humans.

### Taxonomic treatment of *Glinus* in Sub-Saharan Africa

The following treatment provides a new insight into the identification, taxonomic composition and distribution of *Glinus* in Sub-Saharan Africa where the genus is represented by 6 species.

#### Genus Glinus

Taxon classificationPlantaeCaryophyllalesMolluginaceae

L., Sp. Pl. 1: 463 (1753).

ABA6FDF2-F908-5B7E-BB1E-8365C3C4FBE6

≡ Mollugo sect. Glinus (L.) Benth. in Benth. & Mueller, Fl. Austr. 3: 333 (1866).
≡ Rolofa Adans., Fam. Pl. 2: 256 (1763), nom. illeg.
Type: Glinus lotoides L.
= Physa Touars, Gen. Nov. Madagasc.: 20 (1806).
Type: Physa madagascariensis DC., Prodr. 1: 393 (1824) (= Glinus oppositifolius (L.) Aug.DC.)
= Plenckia Raf., Specch. 1: 194 (1814), nom. rej.
Type: Plenckia setiflora (Forssk.) Raf. (≡ Glinus setiflorus Forssk.).
= Tryphera Blume, Bijdr. Fl. Ned. Ind. 11: 549 (1826).
Type: Tryphera prostrata Blume, Bijdr. Fl. Ned. Ind. 11: 549 (1826) (= Glinus oppositifolius (L.) Aug.DC).
= Wycliffea Ewart & A.H.K.Petrie, Proc. Roy. Soc. Victoria n.s. 38: 167 (1926).
Lectotype (designated here): Wycliffea obovata Ewart & A.H.K.Petrie, Proc. Roy. Soc. Victoria n.s. 38: 167 (1926).

##### Description.

Annual, rarely perennial, prostrate herbs or erect subshrubs with a rootstock, covered with stellate or simple (multiseriate, soft and crispate) hairs, in the latter case additionally with multiseriate, stout thick-walled and broad-based hairs (prickles). Stems branched from the base, often forming mats, rarely erect (*G. orygioides*). Leaves in false whorls, lanceolate to obovate, entire or denticulate (mostly in their upper half), with several lateral nerves that can be adaxially recessed and abaxially prominent. Flowers usually several to many (up to 20) in leaf axils forming loose or rarely dense inflorescences, bracteate, sessile or pedicellate. Perianth of 5 free oblong, ovate or roundish segments, green (brown) abaxially and white, pinkish or yellowish adaxially, with a green or brown midvein, horizontally spreading when fully opened, sometimes white petaloid staminodes present, always shorter than perianth segments. Stamens (2–4)5–30, outer stamen series corresponding to another staminode whorl (if stamen number is more than 5) often with filaments terminating with teeth (and without anthers), 0.3–1.8 mm, oblong or roundish; anthers yellow; pollen tricolpate (studied in *G. lotoides*: Perveen and Qaiser 2000). Stylodia 3–5, free or united in lower half into a style. Anthocarp (fruit enclosed by perianth) cylindrical or ovate to roundish. Fruit a hydrochastic loculicidal capsule. Ovary three- or five-carpellate, ovules arranged in two rows. Seeds usually more than 50, yellow, red, brown or black, up to 1.0 mm long, ovoid or reniform, smooth or with numerous colliculae; seed aril divided into two parts: a white, very noticeable hood covering the funiculus and a large ribbon-like appendage, sometimes the hood is reduced. Embryo curved; perisperm abundant and easily visible (in larger seeds) or scanty (in small seeds).

The basic chromosome number in *G. radiatus* is x = 9 (Lane and Keil 1976), which corresponds with that of other Molluginaceae (Bogle 1970, with references therein). However, Mitra and Datta in Löve (1967) indicated the basic number x = 18 for the Indian populations of *Glinus lotoides* and *G. oppositifolius*.

### Artificial key to the *Glinus* species in sub-Saharan Africa based on gross morphology

**Table:** 

1	Plants glabrous or with simple (sometimes additionally with prickle-like) hairs mostly localized in young plant parts	**2**
–	Plants covered with stellate hairs	**3**
2	Stems, sometimes leaf petioles and perianth segments with tiny (up to 0.5 mm) stout prickle-like hairs; perianth segments in fruiting 7.0–9.0 mm long; stamens more than 10; anthers 1.3–1.5 mm long; seed surface with longitudinal ridges bearing colliculae	***G. bainesii***
–	All plant parts without stout prickle-like hairs, or such hairs almost unnoticeable; perianth segments in fruiting up to 5.0(5.5) mm long; stamens (3)5(7); anthers up to 1.0 mm long; seed surface smooth or with colliculae, but in latter case without longitudinal ridges	***G. oppositifolius***
3(1)	Plants white to grayish-green due to abundant stellate hairs; leaves obovate, broadly obovate or roundish, their veins adaxially recessed and abaxially prominent; flower buds or anthocarp (capsules with the closed perianth) ovoid or roundish, 5.0–12.0 × (3.0)3.5–9.0 mm; stigmas usually 5; seeds dark red to black	**4**
–	Plants green; leaves narrowly oblong to obovate, veins neither recessed nor prominent; flower buds and anthocarp cylindrical, 3.0–7.0 × 2.0–3.0 mm; stigmas 3; seeds reddish or brown-red	**5**
4	Glomerules of (1)2–4 flowers; perianth in fruiting 8.0–12.0 mm long; seeds 0.7–0.9 mm long; funicular hood often reduced	***G. setiflorus***
–	Glomerules of 4–12 flowers; perianth in fruiting 5.5–8.5 mm long; seeds 0.5–0.6 mm long; funicular hood easily visible	***G. lotoides***
5(3)	Flowers sessile or with short pedicels up to 3.0(4.0–5.0) mm; perianth in fruiting (3.5)4.0–5.0(5.5) mm long; stamens (3–4)5(6–8); anthers 0.6–0.9 mm long; seeds (0.35)0.40–0.50 × 0.25–0.30 mm	***G. hirtus***
–	Flowers with well-developed pedicels 5–20 mm long, rarely 2–5 mm long; perianth in fruiting 6.5–8.0 mm long; stamens more than 10; anthers (1.0)1.2–1.8 mm long; seeds 0.5–0.6 × 0.35–0.50 mm	***G. zambesiacus***

### List of accepted species in Sub-Saharan Africa (arranged alphabetically)

#### Glinus bainesii

Taxon classificationPlantaeCaryophyllalesMolluginaceae

(Oliv.) Pax, Nat. Pflanzenfam. 3(1b): 40 (1889).

12806517-1129-5685-92B0-65072712BBD1

≡ Mollugo bainesii Oliv., Fl. Trop. Afr. 2: 590 (1871).
Type: [BOTSWANA, North-West distr.] “Koobie [Chobe] to N.[orton] Shaw valley, Jan–Mar 1863, *T. Baines* [s.n.]” (K000232022!).

##### Note.

(1) The so-called “Norton Shaw valley” lies close to Ngami Lake (Passarge 1904), and “Koobie” should be applied to “Chobe”; (2) The type specimen indicated by Adamson (1961) as kept at GRA is absent in this herbarium (Tony Dold, pers. comm.).

##### Description.

**Figure 13.**
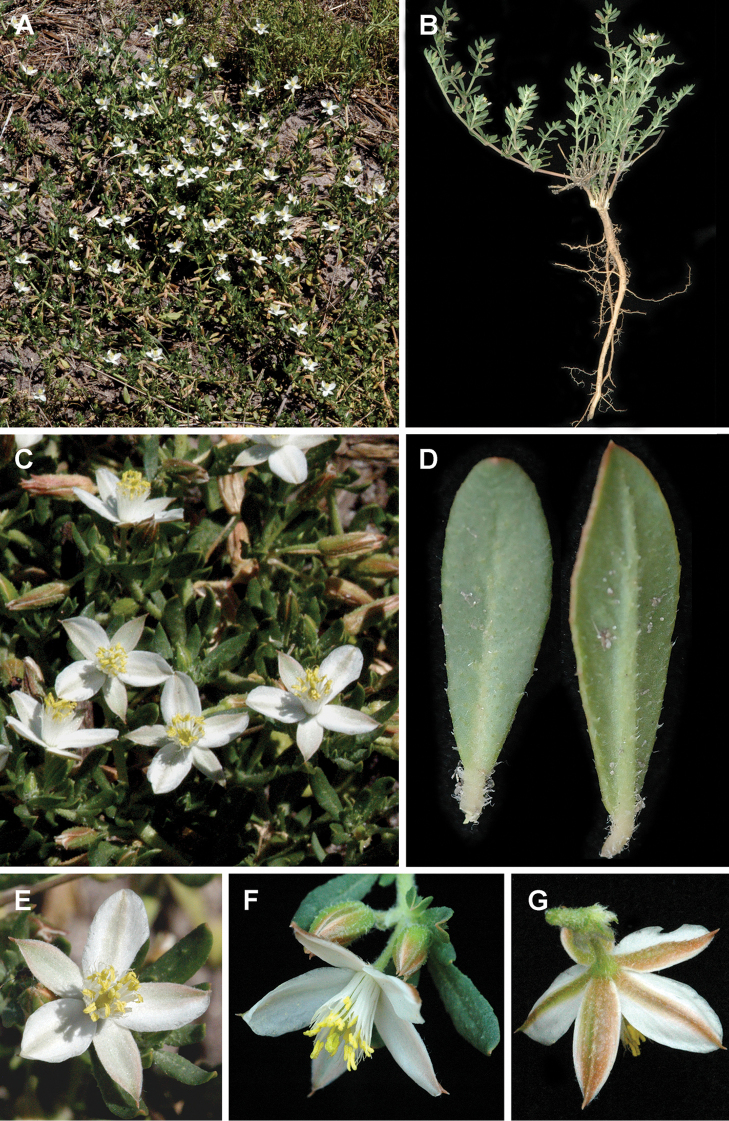
*Glinus bainesii***A, B** an overview of the plant **C** flowers **D** leaves **E–G** close-up of individual flowers. Photographers – Roger and Alison Heath (**A, C, E** Okavango Delta, Ngamiland, Botswana, 28 Nov 2004 **B, D, F, G** Moremi Game Reserve, Ngamiland, Botswana, 21 Nov 2007).

(Fig. 13A–G). Annual or perennial, prostrate or ascending, up to 12 cm tall, often forming large mats up to 1.5 m in diameter. Stems covered with simple crispate hairs; stems, peduncles and sometimes leaf petioles and midveins additionally covered with small, stout hairs (prickles). Leaves rosulate, short-lived, and cauline, green, stout, entire, 10.0–17.0 × 2.0–4.0 mm, oblanceolate or narrowly oblong, sessile or short-petiolate (petioles up to 3.0 mm), mucronulate, veins neither recessed adaxially nor prominent abaxially. Flowers in clusters of 2–7, distant, pedicellate, pedicels 5.0–16.0 mm, in fruiting up to 20.0 mm; buds and closed anthocarp cylindrical. Perianth segments in flowering 6.0–8.0 mm × 2.5–3.0 mm (10–16 mm in diam.), in fruiting 7.0–9.0 mm long, glabrous or sparsely pubescent, dorsally green or with white margins, ventrally white, creamy, pink or pale mauve. Stamens 10–15, outer stamen series (petaloids) sterile, with filaments terminating in teeth; anthers 1.3–1.5 mm long. Stigmas 3, ~1.2 mm long. Seeds 0.6 × 0.5 mm, almost black, with longitudinal ridges carrying colliculate cells; aril hood clearly visible, 0.25 mm long.

##### Remarks.

**Figure 14.**
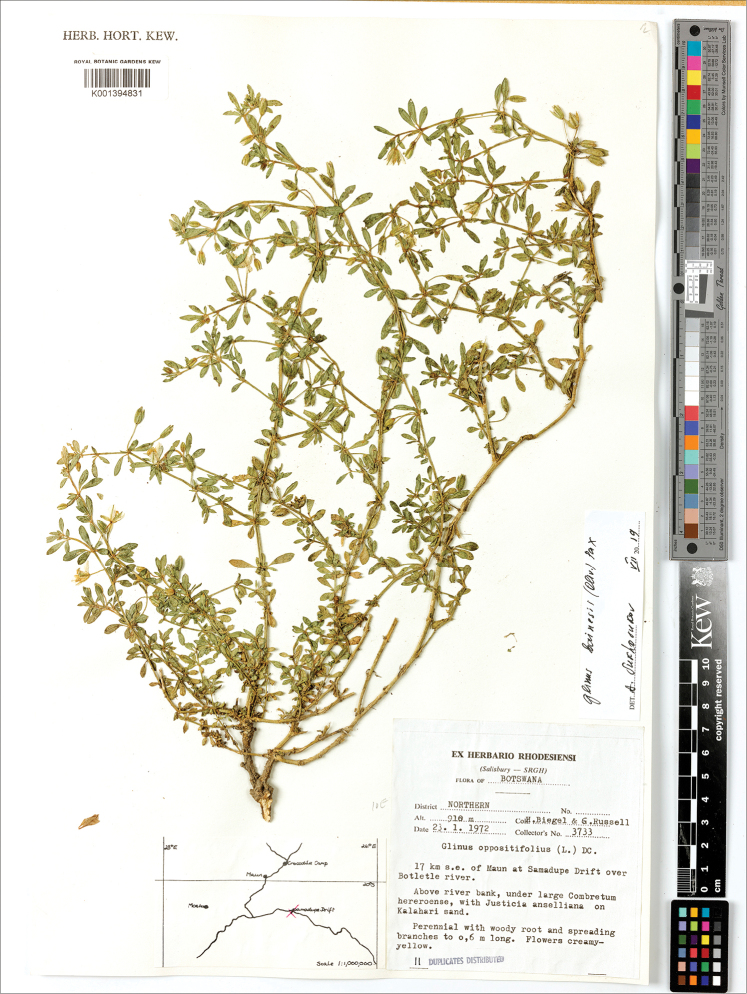
A herbarium specimen of *Glinus bainesii* (Botswana, Northern distr., 17 km SE of Maun at Samadupe drift over Botletle river, 23 Jan 1972, *H. Biegel & G. Russell 3733*, K001394831). Copyright of the Board of Trustees of the Royal Botanic Gardens, Kew.

In the herbaria *G. bainesii* (Fig. 14) is often confused with *G. oppositifolius*, but it is differentiated from the latter species by having small prickles, larger perianth and anthers, and seeds with longitudinal ridges (Table 5).

##### Habitat.

Riverbed sands, flat flood plains, margins of muddy seasonal pans, sparsely vegetated areas at elevations 0–1200 m a.s.l. Associated plants found in Botswana: *Dicerocaryum eriocarpum*, Pterococcus oppositifolius var. oppositifolius, poor soil Cypreaceae and Poaceae spp. (*A. Heath & R. Heath 734*, K); *Fuirena pubescens*, *Neptunia oleracea*, *Hibiscus meeusei*, *Cyperus compressus*, *Senna obtusifolia* (*A. Heath & R. Heath 428*, K); *Vahlia capensis*, *Heliotropium ovalifolium*, *Cynodon dactylon*, *Streptoglossa decurrens*, *Jamesbrittenia elegantissima* (*A. Heath & R. Heath 1417*, K). Flowers during both the early and the main rains.

##### Distribution

**Figure 15.**
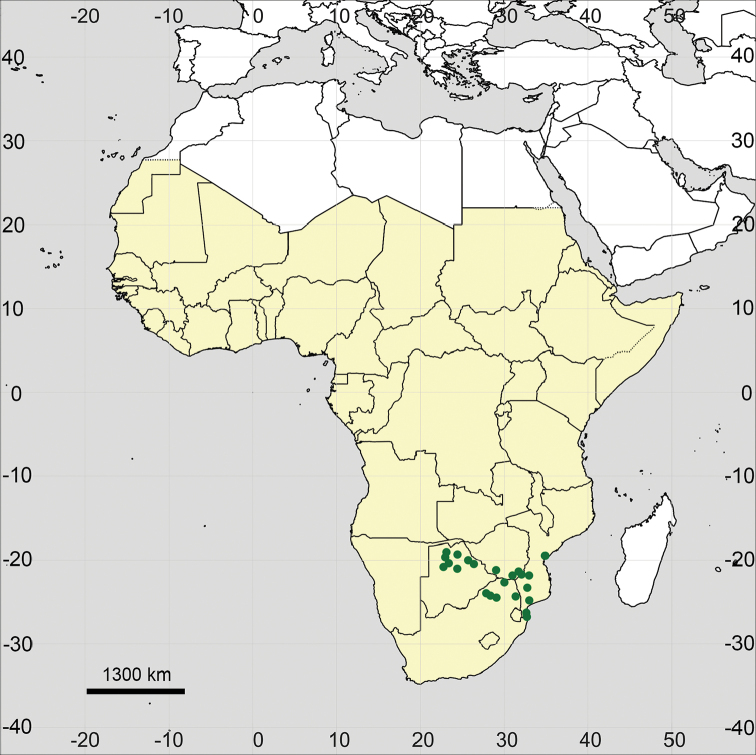
Distribution map of *Glinus bainesii* in Sub-Saharan Africa (coloured in yellow).

(Fig. 15). Botswana: Ngamiland, Kwebe Hills, 3300 ft, 4 Feb 1898, *E.J. Lugard 152* (K); Northern Bechuanaland, Sigere pan, 30 miles W of mouth of Nata river, 896 m, 25 Apr 1957, *Drummond & Seagrief 5216* (K); Ngamiland, Sehitwa, Lake Ngami, 930 m, 25 Mar 1961, *H.M. Richards 14849* (K); Northern distr., 17 km SE of Maun at Samadupe drift over Botletle river, 23 Jan 1972, *H. Biegel & G. Russell 3733* (BR0000017454490, K001394831, M, P04577251); Northern distr., Moremi reserve, 19°10.4'S, 23°15.7'E, 26 Jan 1974, *P.A. Smith 848* (B101143636, K, WAG1103318); Northern distr., Nata river delta, 21 Apr 1974, *J.F. Ngoni 529* (K, PRE0825248); Ngami Lake, 13 Dec 1982, *P.A. Smith 3981* (BR0000017454506, E, K); between Motswiri and TFC [Tsetse Fly control] road, 18°45.671'S, 23°15.033'E, 966 m, 19 Mar 2003, *A. Heath & R. Heath 428* (K); 17 km S of Tsetse Fly control road, 18°45.565"S 23°05.208"E, 964 m, 28 Nov 2004, *A. Heath & R. Heath 734* (K); Khwai River floodplain, Moremi Game Reserve, 19°10.505'S, 23°44.176'E, 940 m, 21 Nov 2007, *A. Heath & R. Heath 1417* (K); Ngamiland, 100 m S of Samudupe bridge, 20°6'59"S, 23°31'38"E, 938 m, 19 Feb 2008, *B. Farrington et al. 486* (K);

Mozambique: Lorenço Marques [Maputo], 7 Dec 1897, *B. Schlechter 11643* (BM, BR0000018267990, E, G, LE, P04577163, WAG1103122); Sofala [prov.], Nov 1901, *Anonymous 2654* (WAG1103309); Lorenço Marques [Maputo], 21 Mar 1920, *Berle 761* (B101143603, BR0000018267983, M); Lorenço Marques [Maputo prov.], between Umbeluzi and Porto Henrique, 20 Nov 1940, *A.R. Torre 2090* (K); Gaza prov., Aldeira da Barragem, 20 Nov 1957, *L.A.G. Barbosa & F. de Lemos 8222* (K); Gaza prov., Caniçado, 23 Aug 1969, *M.F. Correia & A. Marques 1158* (WAG1103272); Gaza prov., Limpopo, Massangena, 21 Jul 1973, *M.F. Correia & A. Marques 2979* (K, M, WAG1103326); Gaza prov., Caniçado, 6 Aug 1973, *M.F. Correia & A. Marques 3184* (WAG1103323); Lorenço Marques [Maputo prov.], Porto Henrique, Bela Vista, 25 Mar 1975, *A. Marques 2670* (WAG1103271); Maputo, 13 Dec 1979, *J. de Koning 7754* (BR0000013706678, BM, K);

South Africa: [Limpopo province] Messina [Musina], alt. 2000 ft, Sep 1917, *F.A. Rogers 19299a* (BM, G); Transvaal [Limpopo prov.], Messina [Musina], 27 May 1927, *R.G. Young 18391* (BM); Transvaal prov., Ellisras, 2900 ft, 24 Feb 1954, *L.E. Codd 8491* (K, L1698855); Transvaal [Limpopo prov.], Potgietersrust [Mokopane], Doornpoort Farm, 19 Jan 1955, *A.D.J. Meeuse 9552 & 9552a* (K, M); Transvaal [Limpopo prov.], Waterberg distr., Tamboetie river, Ellisras–Vila Nova road, 7 Jan 1959, *A.D.J. Meeuse & R.G. Strey 10446* (BM, BOL217406, BRLU0026260, K, M, Z-000092195); Transvaal [Limpopo prov.], Kruger NP, 31 Jan 1962, *H. Schlieben 9321* (W20978); Mpumalanga prov., Kruger NP, 26 Nov 2015, *G. Zambatis 1195* (PRE0990262);

Zimbabwe: [Masvingo prov.] Sabi-Lundi Junction District, Chiribira Falls, 6 Jun 1950, *Wild 3448* (B101143602, BR0000018267976, K); [Matabeleland South prov.] Gwanda Distr., 700 ft, Nov 1956, *R.M. Davies 2185* (K); [Masvingo prov.] Nuanetsi, nr Malipate, 2 May 1961, *R.B.Drummond & R.O.B. Rutherford-Smith 7680* (K); [Masvingo prov.] Chiredzi, nr Sabi-Ludi junction, 31 May 1971, *S. Mari 1273* (K, LE);

##### General distribution.

Endemic to Zambezi floristic province (according to Takhtajan 1986). Reported from Okavango region, NE Namibia (Friedrich 1966), but the cited specimen (“*Lightfoot 65*”) has not been found by us (SAM?).

#### Glinus hirtus

Taxon classificationPlantaeCaryophyllalesMolluginaceae

(Thunb.) Sennikov & Sukhor. comb. nov.

4CF5D934-5306-5412-B2B6-0B7C902C94C4

urn:lsid:ipni.org:names:77215157-1

≡ Mollugo hirta Thunb., Prodr. Pl. Cap. 1: 24 (1794).
≡ Pharnaceum hirtum (Thunb.) Spreng., Syst. Veg., ed. 16, 1: 949 (1824).
≡ Glinus lotoides subsp. hirtus (Thunb.) M.R.Almeida, Fl. Maharashtra 2: 342 (1998) (as “hirta”).
Lectotype (designated here): South Africa. “E Cap. bon. spei” [Cape of Good Hope], *C.Thunberg* (UPS-Thunb 2851!; isolectotype UPS-Thunb 2850!).
= Glinus dahomensis A.Chev., Fl. Afrique Occ. Franc. 1: 323 (1938), nom. inval. (Art. 39.1).
Original material: [Benin] Dahomey, Kouandé à Kontobiri entre Quétécou et Firou [from Kouandé to Kontobiri between Quétécou and Firou], 29 Jun 1910, *A. Chevalier 24288* (P00461735!).
– Glinus congolanus Hauman in herb. BR.

##### Description.

**Figure 16.**
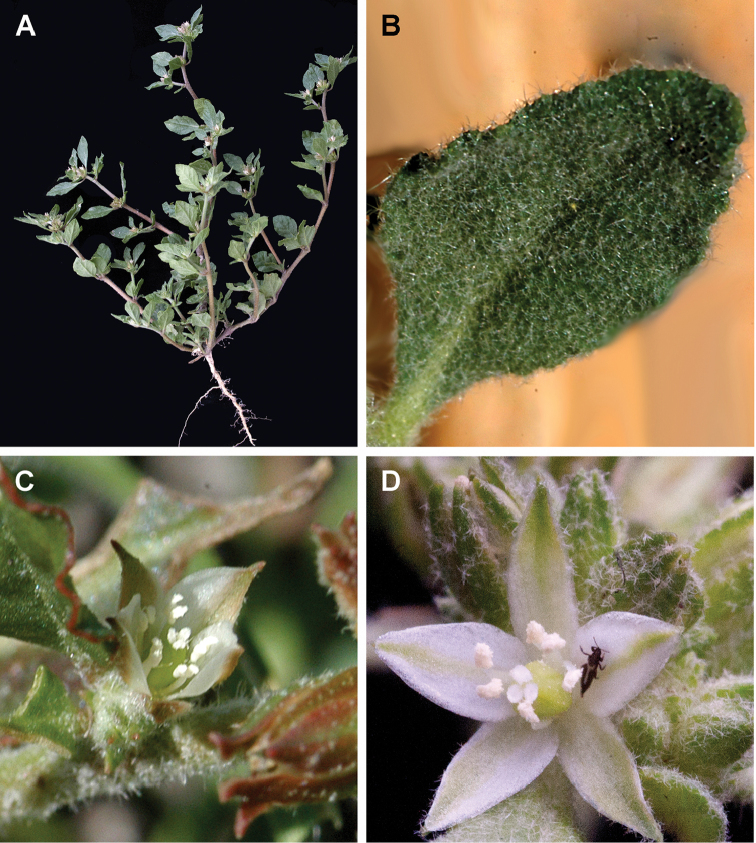
*Glinus hirtus***A** an overview of the plant **B** close-up of the leaf **C, D** flowers. Photographers – Roger and Alison Heath (**A, D** Moremi Game Reserve, Ngamiland, Botswana, Nov 2010 **B, C** Selinda, Ngamiland, Botswana, 04 Nov 2004 (**B**), 23 Jan 2004 (**C**).

**Figure 17.**
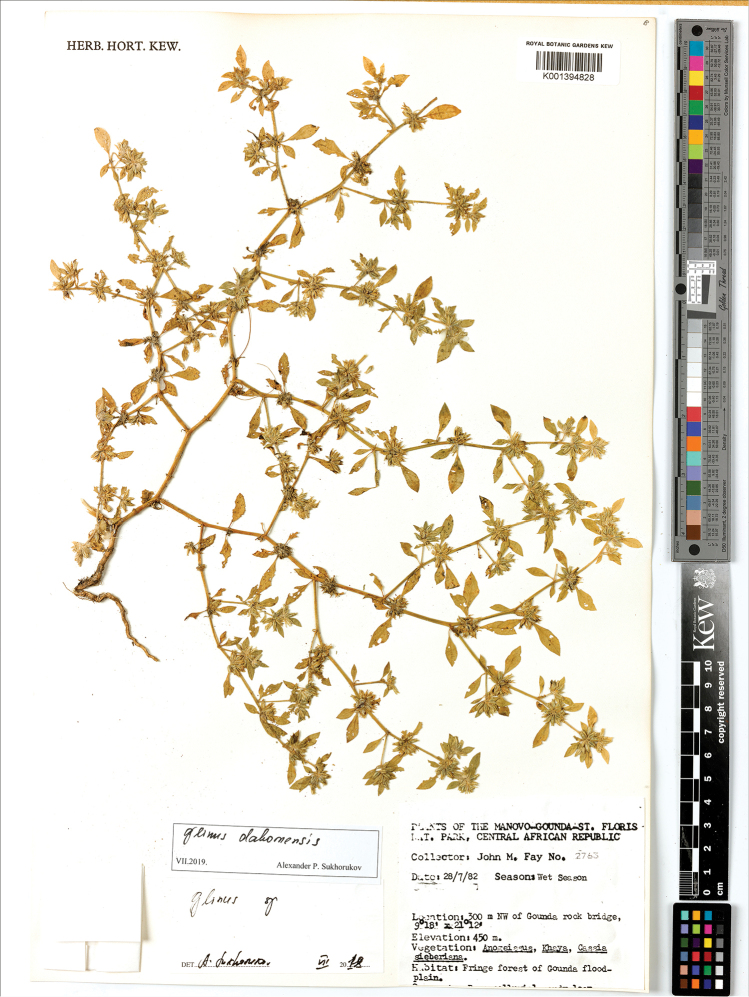
A herbarium specimen of *Glinus hirtus* (Central African Republic, 300 m NW of Gounda, 9°18'N, 21°12'E, alt. 450 m, 28 Jul 1982, *J.M. Fay 2763*, K001394828 as *G. dahomensis*). Copyright of the Board of Trustees of the Royal Botanic Gardens, Kew.

(Figs 16, 17). Annual, highly branched, with prostrate or ascending stems up to 100 cm long, covered with stellate (sometimes very scattered) and branched hairs, prickles absent. Leaves rosulate, short-lived, and cauline, green or grayish-green turning red at senescence, sparsely to moderately pubescent, rarely glabrous, petiolate (petioles up to 10.0 mm), entire or slightly crisp or scarcely denticulate, ovoid, obovate or oblong-spatulate, 10.0–40.0(45.0) × 3.0–15.0(18.0) mm, acuminate, lateral veins neither recessed adaxially nor prominent abaxially. Flowers in clusters of 8–20, distant or approximated in the upper part of the inflorescence, 8–13 mm in diameter, sessile or with short pedicels up to 3.0(4.0–5.0) mm; buds and closed anthocarp cylindrical. Perianth segments in flowering 3.0–4.2 mm long, in fruiting (3.5)4.0–5.0(5.5) mm long, glabrous or sparsely pubescent, dorsally green or with white margins and ventrally white or mauve; tips ± recurved at fruiting. Stamens (3–4)5(6–8), sometimes with sterile teeth; anthers 0.6–0.9 mm long. Stigmas 3, 0.5–1.0 mm long. Seeds reddish or brown-red, (0.35)0.40–0.50 × 0.25–0.30 mm, colliculate or rarely almost smooth, longitudinal ridges absent.

##### Note.

*Mollugo hirta* described from South Africa was very rarely accepted in old taxonomic literature, and only cited as a poorly known species (Fenzl 1836). Otherwise, it has been commonly considered a synonym of *G. lotoides* (e.g., Fenzl 1840; Harvey and Sonder 1860; Just 1879; Adamson 1961; Hedge and Lamond 1975; Gonçalves 1978). We state for the first time that (1) *Mollugo hirta* must be resurrected in specific rank based on both molecular and morphological studies as *G. hirtus*, and (2) this species is conspecific with the name *G. dahomensis*. Specimens of this species were misidentified in collections with various names, particularly *G. lotoides*, G. lotoides var. virens, *G. lotoides* × *G. oppositifolius*, *G. spergula*, and *Mollugo glinoides* (both latter names belong to the synonymy of *G. oppositifolius*). Almeida (1998) referred the Indian plants to G. lotoides subsp. hirtus in the belief that they differ from the European populations (G. lotoides subsp. lotoides), but he did not indicate any differences between them. However, his description of the subspecies rather belongs to *G. lotoides* based on the number of stamens (>10) and stigmas (5), while *G. hirtus* has up to eight stamens and three stigmas.

The name *G. dahomensis* was originally introduced (Chevalier 1938) with a description in French, whereas the nomenclatural code required a description or diagnosis in Latin. To date the name *G. dahomensis* remains invalidly published because of having been commonly placed in the synonymy of G. lotoides var. virens.

While considering *G. hirtus* distinct from *G. lotoides*, we resurrect the name published by Thunberg because it is the only one available for the species as circumscribed in our study.

*Glinus hirtus* is morphologically very close to *G. ononoides* Burm.f. described from India (Burman 1768), a species completely forgotten in the past (Dizionario… 1842) and present. The differences between both species are minor and limited to the following characters: (1) the type of *G. ononoides* has stellate hairs that are mostly localized on the young stem parts and leaves and additionally very short (up to 0.4 mm) prickle-like simple hairs present on the stem. In contrast, *G. hirtus* has well-expressed stellate pubescence on stem and perianth, and no prickle-like hairs, and (2) the seeds of *G. ononoides* are smooth, whereas those of *G. hirtus* are usually colliculate (however, the seeds of the specimens from Senegal, [without date] *Perrottet*; Botswana, 1982, *P.A. Smith*; and Somalia, 1893, *D. Riva*, are smooth). The collections of *G. ononoides* are very scarce, and the variability of the characters mentioned and the distribution of the species in Asia require further investigations. The morphologically very similar, but phylogenetically distant *G. radiatus* distributed in South and Central America has shorter anthers (0.35–0.6 mm long) and stylodia (0.2–0.6 mm long) as well as smooth seeds; by contrast, *G. hirtus* has longer anthers (0.7–0.9 mm) and stylodia (0.5–1.0 mm) and its seeds are usually colliculate (rarely smooth). Also, the distribution areas of both species do not overlap.

##### Habitat.

River beds, wetlands, damp areas or as a weed, mostly on sandy soils at elevations 0–2400 m a.s.l. Sometimes, *G. hirtus* is found growing together with *G. oppositifolius* (collections of *Cook 405 & 408* from Nigeria, K!). Observations in Botswana recorded the following associated plants (*A. Heath & R. Heath 457*, K): *Cynodon dactylon*, *Sida cordifolia*, *Glinus bainesii*, *Portulaca oleracea*, *Cyperus polystachyos*, *C. compressus*, *C. longus*, *Pseudognaphalium luteo-album*. Flowers in early and main rains (obs. in Botswana by A. Heath and R. Heath).

##### Distribution

**Figure 18.**
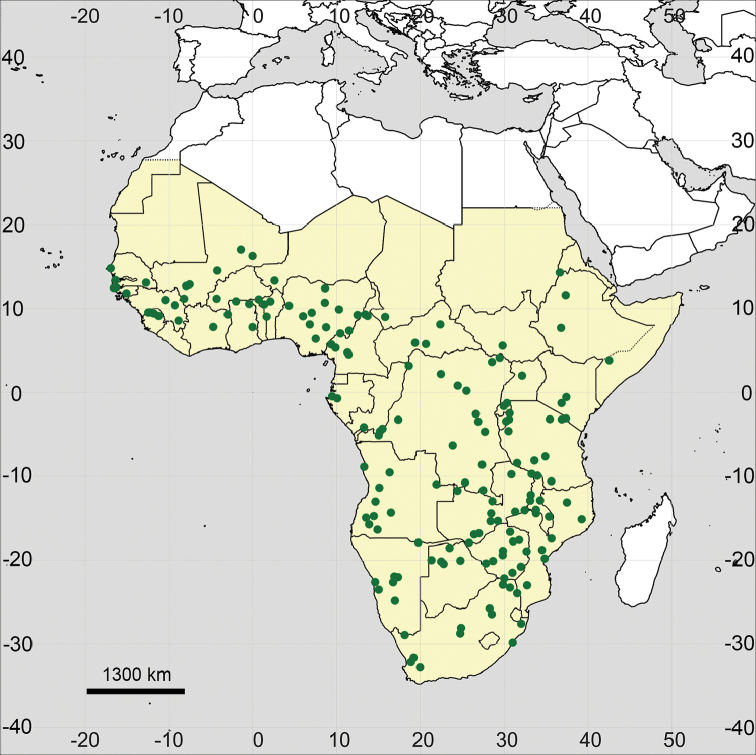
Distribution map of *Glinus hirtus* in Sub-Saharan Africa (coloured in yellow).

(Fig. 18). The species was originally known as *Mollugo hirta* from South Africa (Thunberg 1794) and not reported as a distinct species from any other African territory. *Glinus dahomensis* was described from Benin (Chevalier 1938), and later reported from Belgian Congo (DR Congo) (Hauman 1951). Hauman (1951) also noted the presence of the species in other territories of tropical Africa (“Du Dahomey au Transvaal”). However, he probably was not sure of that and did not reidentify the specimens from any other countries. We confirm that the range of *G. hirtus* is not restricted to Benin and DR Congo, but the species is distributed in all sub-Saharan Africa and seems to be a common weed in many regions according to the collector’s observations. *Glinus hirtus* has not been previously reported for almost any Sub-Saharan countries (e.g., Adamson 1961; Jeffrey 1961; Berhaut 1967; Gonçalves 1970, 1978; Sita and Moutsambote 1988; Barry and Celles 1991; Mapaura and Timberlake 2004; Phiri 2005; Setshogo 2005; Hassan et al. 2005; Sosef et al. 2006; Germishuizen et al. 2006; Darbyshire et al. 2015). In some West African checklists and manuals it was confused with the American *G. radiatus* (Berhaut 1967, 1979; Boudet et al. 1986; Akoegninou et al. 2006; Lisowski 2009; Thiombiano et al. 2012; Brundu and Camarda 2013; Schmidt et al. 2017). Only a few specimens from Burkina Faso, DR Congo, and Nigeria were correctly identified as *G. dahomensis*.

Angola (new records): [Moxico prov.] Chizanda, 23 Sep 1899, *H. Baum 135* (BM, BR0000018269482, K, M); [Malanje prov.] Malange [Malanje], 21 Sep 1932, *R.G.N. Young 846* (BM); Huila [prov.], Lubango, 11 Dec 1955, *E.J. Mendes 1083* (BM, LISC032007); Luanda, Namuculungo, 1100 m, 13 Nov 1957, *J.B. Teixeira 3185* (LISC032013); Huila prov., Canguelas, Vila Artur de Paiva, 1450 m, 30 Dec 1959, *E.J. Mendes 1879* (M); Benguela prov., Ganda, 30 Jun 1960, *J.M. Teixeira & A.M. Andrade 4988* (LISC032014); Cuanza Sul prov., Uaco-Cungo, 2 Nov 1961, *J.M. Teixeira & J.M. Figueira 5956* (LISC032015); [Cunene prov.] Mucope, Tchica [river], 9 Nov 1963, *C. Henriques 217* (BM, K, LISC032005); Huila prov., Gambos, 18 Nov 1970, *R.M. Santos & E. Barroso 2874* (LISC032010); Huila prov., Quipungo, 1200 m, [without date] *J.M. Teixeira 12580* (LISC032004);

Benin: see type of *G. dahomensis*; Atakora [dept.], Toukountouna, 12 Apr 1999, A. *Akoegninou et al. 2440* (WAG0235864); Atakora [dept.], Kérou, 16 Apr 1999, *A. Akoegninou et al. 2568* (WAG0235868); [Donga dept.] Bassila, 460 m, 30 May 2001, *A. Akoegninou 4810* (WAG0235872);

Botswana (new records): Northern distr., Nxauna Pan, 1 Jan 1973, *P.A. Smith 318* (K); [North-West distr.] nr Tsau–XaiXai road, 19°55'2"S, 21°24'4"E, 21 Apr 1982, *P.A. Smith 3847* (BR0000018269659; PRE0671232, PRE0520975); [Ngamiland distr.] Ngami Lake, 13 Dec 1982, *P.A. Smith 3980* (BR0000018269666, E, G, PRE0671267); Ngamiland distr., between Caecae and Gwihaba Hills, 1000 m, 15 Mar 1987, *D.Anonymous Long & D.A.H. Rae 300* (E, K); Zibalianja lagoon, 18°34.728'S, 23°32.145'E, 957 m, 23 Jan 2004, *A. Heath & R. Heath 457* (BACH, K);

Burkina Faso (new records): Kompienga [prov.], 40 km E of Tindangou, 2 May 2003, *L. Sanou & M. van Slageren 1352* (K); Houet [prov.], 18 km NW of Bobo Dioulasso, 12 May 2003, *L. Sanou & M. van Slageren 1390* (K);

Burundi (new records): [Karuzi prov.] Nyabibuye, 4500 ft, 15 Nov 1960, *R. Tanner 5602* (K, WAG1103206); [Ruyigi prov.] Ruyigi, 2°59'S, 30°28'E, alt. 1450 m, 27 Sep 1978, *M. Reekmans 7098* (K);

Cameroon (new records): Southwest region, Mamfe distr., 15 Mar 1953, *C.F.A. Onochie et al. 30889* (K); [Centre Province region] 62 km SE of Bafia, 27 Mar 1963, *J. Raynal & A. Raynal 10537* (P0456668); [North region] nr Garoua, Benoue river, 18 Dec 1964, *W.J.J.O. de Wilde et al. 4959* (WAG0180494); [Litoral region] plaine des Mbo, 7 km E of km 25 Melong–Dechang (Dschang) road, 5°11'24"N, 10°1'12"E, alt. 700 m, 14 Apr 1972, *A.J.M. Leeuwenberg & C.C. Berg 9607* (BR0000018268621, K, P04576510, WAG0185131); North region, Bénoué NP, 7 May 1974, *C. Geerling & J. Néné 4705* (BR0000018268591, WAG0330521); [Centre region] river Sanaga, bridge nr Nkol Ngok, 4°10'N, 11°01'E, alt. ~300 m, 15 Feb 1978, *J. Lowe 17675* (K);

Central African Republic (new records): Kémo [pref.], 17 Feb 1892, *Anonymous 691* (P04577135); Ouaka pref., Bambari, 7 May 1927, R.P. Tisserant 2180 (p04577138); [Haute-Kotto pref.] 300 m NW of Gounda, 9°18'N, 21°12'E, alt. 450 m, 28 Jul 1982, *J.M. Fay 2763* (K001394828);

Chad (new record): [Logone Occidental region] Kim [Krim], 18 May 1969, *Anonymous Fotius 1458* (P04576514);

DR Congo (selected): [nr Kinshasa] Stanley Pool [Pool Malebo], 1888, *F. Hens 29* (BR0000018268034); [Kongo Central prov.] Kisantu, 1900, *J. Gillet 1386* (BR000018268027); Kongo Central prov.] Kisantu, 1903, *J. Gillet 3029* (BR000018268010); [Kasai prov.] Zambi vill., 27 Jun 1915, *Bequaert 7885* (BR000018268003); [Kwilu prov.] Wombali [Bandundu], Oct 1915, *H. Vanderyst 2316* (BR0000018268171); Haut-Katanga prov., Kafubu, 18 Nov 1927, *Quarré 804* (BR0000018268386); [Mongala prov.] Bumba, 19 Feb 1931, *R. Letouzey & J.F. Villiers 10386* (K, P04576657, WAG0185129); [Haut-Uele prov.] Garamba NP, 20 Feb 1950, *H. de Saeger 147* (BR0000018268232); Orientale prov., Dungu, 2 Apr 1951, *H. de Saeger 1084* (K); Orientale prov. [Haut-Uele prov.] Dungu, Garamba NP, 28 Mar 1951, *H. de Saeger 1119* (K); [Lualaba prov.] Dikuluwe, 21 Aug 1956, *J. Brynaert 520* (BR0000018268324, K); [Haut-Katanga prov.] Mitwaba, 2 Nov 1956, *J. Brynaert 541* (BR0000018268317, K, LE, L1698856, M, W13591); [Tshopo prov.] Yangambi, 5 Feb 1958, *R. Devred 2925* (WAG0185147); [Kasai-Oriental prov.] Katanda Territory, 17 Sep 1959, *L. Liben 3701* (BR0000017455121); [Maniema prov.] Kabambare Territory, 10 Aug 1959, 570 m, *A. Leonard 5736* (BR0000018268201, WAG0185149); [Maniema prov.] Kamisuku, 700 m, 20 Aug 1959, *A. Leonard 6034* (BR0000018268218, P04576678, WAG0185148); Yangambi prov., Bangi, Ile Yangambi, 7 Sep 1959, *P. Bamps 700* (BR0000018268188, K); [Haut-Lomami prov.] Bukama, Lualaba river, 570 m, 2 Oct 1970, *M. Lukuesa 758* (BR0000018268355, WAG0185146); Kongo-Central prov., Kasangulu, Sabuka, 6 Apr 1972, *C. Evrard 6929* (BR0000018269673); Haut-Katanga prov., Kumanua, 1150 m, 18 Dec 1975, *F. Malaisse 8869* (BR0000018268379); [Eastern prov.] Waine-Rukula, 22 Oct 1978, *J. Lejoly 4180* (BR0000017455336); [nr Kinshasa] Inkisi river, 17 Oct 2007, *Nsimundele 2060* (BR0000000530230);

Ethiopia (new records): [Amhara region] Begember prov., [years] 1863–1868, *Schimper 1340* (K); [Oromia region] Jimma, Apr 1957, *Anonymous S2* (K); Godjam prov. [Amhara region], Bahir Dar, 1800 m, Jun 1968, *O. Sebald 2166 & 2255* (M, STU);

Gabon (new records): Moyen-Ogooué prov., Lambaréné, 17 Oct 2012, *Boupoya 760* (BRLU0026277); Ogooué-Maritime prov., Wonga-Wongué Reserve, 20 Oct 2014, *A. Boupoya & al. 1087* (BRLU0026275); [Moyen-Ogooué prov.] Onangué Lake, 35 km SE of Lambaréné, 22 Oct 2014, *E. Bidault et al. 1820* (BR0000016174696, BRLU0026276, P00854718);

Gambia (new record): [North Bank Division] Albreda, 1839, *Haudelot 100* (G);

Ghana: [Oti Region], on road to Oti from Kete Krachi, 18 May 1952, *J.K. Morton 7294* (K); [Upper West region] Tumu, edge of Dam, 25 May 1952, *J.K. Morton 7554* (K, WAG0185143); [North East region] Gambaga, 26 Dec 1954, *J.K. Morton A1378* (K);

Guinea (new records): [Kankan region] Yri Kiri, 19 May 1955, *Anonymous Roberty 18001* (G); [Kankan region] Kankan, Bordo, 26 Apr 1956, *J.-Anonymous Adam 12119* (P00695349); [Nzérékoré / Kankan Regions] Simandou Range, Oueléba swamp, alt. 1270 m, 19 Oct 2008, *I. Darbyshire 495* (K000580882); [Nzérékoré / Kankan Regions] Simandou Range, Canga East, May 2009, *P.K. Haba 579* (K000024353, P00990780, WAG1476650);

Guinea-Bissau (new record): [Bafatá Region], Bambadinca, 11 Jun 1945, *Anonymous 2065* (K, WAG0185158);

Ivory Coast (new records): [Vallée du Bandama distr.] Fétékro, 18 Jan 1947, *Anonymous Roberty 6915* (G); [Bouna dept.] Bouna, 1 Dec 1986, *P. Poilecot 3833* (G40376);

Kenya (new records): [Nairobi] Nairobi Dam, 31 Jan 1961, *C. Teesdale 26* (K); Nairobi National Park, 1525 m, 7 Oct 1977, *J.B. Gillett & W.T. Staam 21582* (BM, K); [Eastern prov.] 36 km from Embu town, 14 Nov 1979, *AnonymousW. Gatheri et al. 79/1* (K);

Malawi (new records): [Northern region] nr Karonga, 1893–1894, *AnonymousF. Scott-Elliot 8403* (BM, K); Zomba, 2500–3500 ft, *A. Whyte s.n*. (G, P04576503); Nyasaland [without exact location], 1895, *J.Buchanan 15* (BM, BR0000018269611, E); [Central region] Dedza Distr., Chongoni Forest reserve, 18 Sep 1967, *A.J. Salubeni 832* (K); [Central region] Kasungu distr., Kasungu NP, 12 Dec 1970, *A.J. Hall-Martin 1283* (K); [Central region] Kasungu distr., Kasungu NP, 1000 m, 23 Dec 1976, *J. Pawek 12032* (K, WAG1103230); [Southern region] Liwonde NP, 9 Oct 1978, *A.J. Salubeni & E. Tawakali 2339* (WAG1103225); Central Region, Mchinji distr., 21 Nov 1983, *A.J. Salubeni & I.H. Patel 3484* (BOL217407); [Central region] Lilongwe Distr., Nature Sanctuary, 8 Nov 1984, *I.H. Patel & al. 1656* (K); Central Region, Nkhotakota distr., 13 May 1986, *I.H. Patel & R.B. Kwatha 3184* (BRLU0026262); Northern region, Chitipa, 9°58'51"S, 33°25'37"E, 1528 m, 18 Dec 2007, *H.T. Chapama & al. 788* (K);

Mali (new records): [Koulikoro region] Koulikoro, 11 May 1912, *Vuillet 609* (P04577141); [Inner Niger Delta region] Mopti, 24 Apr 1932, *J. Rogeon 76* (P04577146); [Gao region] Bamba, 1927, *O. Hagerup 327* (BR0000018268447, K, P04576677); [Gao region] Gao, 21 Jun 1936, *M. de Wailly 5070* (P04577142); [nr Bamako] Niger river, Sotuba, 4 Jul 1973, *D. N’Golo 1291* (WAG0319754); [Sikasso region] Sikasso, Yanfolila, 7 Jul 2009, *L. Sanou et al. 624* (K);

Mozambique (new records): [Nampula prov.] Nampula, Mutivasi river, 3 Nov 1942, *F.A. Mendonça 1217* (K); [Zambezia prov.] Boroma, 4 miles from Zambesi river, 25 Jul 1950, *N.C. Chase 2788* (BM); [Sofala prov.] Gorongosa distr., 300 ft, 26 Sep 1953, *N.C. Chase 5087* (BM); Sofala prov., Gorongoza Game Reserve, 25 Aug 1958, *N.C. Chase 6980 & 6981* (BR0000018269185, FT0007098, K); Sofala prov., Gorongosa NP, 3 Nov 1963, *A.R. Torre & J. Paiva 9006* (BR0000018269161, M); [Sofala prov.] Beira, Jul 1970, *K.L. Tinley 1936* (BR0000018269642); Sofala prov., Gorongosa NP, Jul 1970, *K.L. Tinley 1938* (K); Gaza prov., Caniçado, 17 Nov 1970, *M.F. Correia 2018* (WAG1103222); Niassa prov., Lugenda river, Marrupa to Mecula, 11 Aug 1981, *P.C.M. Jansen et al. 225* (K, WAG0312529); Sofala prov., Gorongosa NP, 18°55'4.8"S, 34°30'38.04"E, 9 Nov 2007, *P. Ballings & B. Wursten 944 & 958* (BR581135 & BR0000018269635);

Namibia (new records): [Otjozondjupa Region] Okahandja, Nov 1906, *K. Dinter 301* (BM, BR0000018269222, E, G, K, P04577101, U139844, WU, Z-000092198); [Khomas region] Windhoek, 21 Dec 1929, *C.E. Moss 18055* (BM); [Khomas region] Khomas Highland, Friedenau Farm, 2000 m, 6 Apr 1939, *Anonymous Gassner 124* (M); [Erongo region] Swakopmund distr., Goabeb Research Station, 9 Oct 1963, *C. Koch s.n*. (K, PRE0403669); [Hardap region] 30 miles E of Maltahöhe, 4400 ft, 15 Feb 1950, *E.C.Macdonald 369* (BM); [Kavango-East region] Rundu [between Kapuko & Sambiu], 22 Dec 1955, *B. de Winter 4035* (K, M, PRE0403672); [Khomas region] Windhoek, 1575 m, 29 Apr 1961, *R. Seydel 2819* (B101143626, G, L1693130); [Erongo region] Swakopmund, mouth of Swakop river, 11 Feb 1962, *R. Seydel 3041* (B101143623, M); [Khomas region] Windhoek, Swakop river, 2000 m, 20 Feb 1965, *R. Seydel 4194* (B101143629, B101143630, K);

Niger (new record): [Tillabéri region] Kouré, 8 Mar 1897, *Anonymous Roberty 2556* (P04577117);

Nigeria (new records): [Benue State] Abinsi, 29 May 1912, *J.M. Dalziel 715* (K); [Jigawa State] Takara, 3200 ft, 4 May 1921, *H.V. Lely 101* (K); Adamawa State, Jimeta (Yola), 29 Dec 1957, *F.N. Hepper 1621* (K); [Niger State] Shagunu, 10 km N of Bussa, 25 Jul 1965, *C.D.K. Cook 405* (K); Niger State, Bida distr., 1 Mar 1968, *B.O. Daramola & A. Binuyo 61930* (K); [Taraba State] Sardauna area, Bissaula, 18 Dec 1968, *B.O. Daramola 62328* (K); [Taraba State] Gashaka distr., Gashaka river, 1100 ft, 25 Mar 1970, *Z.O. Gbile & B.O. Daramola 23888* (WAG0185150); Bauchi State, Yankari Game Reserve, 24 Mar 1971, *C. Geerling 3486* (B100480213, BR0000017456135, WAG0083282); Gombe State, Dadin Kowa, Gongola river, 2 May 1972, *Gbile et al. 65453* (K); Adamawa State, Yola distr., 6 May 1972, *Gbile et al. 1303* (K); Enugu State, Amechi vill., 21 Feb 1973, *Latilo & Oguntayo 67620* (K, WAG0185136); [Kogi State] Koton Karfe, Niger river, 22 Mar 1973, *Eimunjeze et al. 70400* (K); Niger State, Kafin Koro, 23 May 1973, *Eimunjeze et al. 66496* (K);

Republic of the Congo (new record): [Bouenza dept.] Jacob [Nkayi], 27 Sep 1969, *Y. Attims 255* (WAG00034942);

Rwanda (new records): Mutara area, 1400 m a.s.l., 3 Oct 1956, *Anonymous Troupin 2810* (BR0000017455824, K); [Eastern prov.] Nyagatare, 1450 m, 27 Jan 1958, *Anonymous Michel 5077* (BR0000017455817);

Senegal (new records): [without exact location and date] *Perrottet 373* (G, P04577123); Wab, 20 Dec 1824, *Perrottet 51* (P04577122); [Waalo Region] N’ghiau, [without date] *Lepier s.n*. (G); Oussodou, 2 Jan 1954, *R.P. Berhaut 4330* (P04577147); [Ziguinchor Region] Bignona, 22 May 1957, *J.-Anonymous Adam 13656* (P04577113); Niokolo Koba NP, Feb 1960, *J.-Anonymous Adam 17485* (P04577114); Basse Casamance [Ziguinchor], 11 Jul 1963, *R.P. Berhaut 6084* (BR7000919, P04577119); Basse Casamance [Ziguinchor], 29 Jul 1977, *C. Vanden Berghen 1915* (BR0000018268461, WAG0106332);

Sierra Leone (new records): [Northern prov.] Samaia, 10 May 1914, *N.W. Thomas 245* (K); [Northern prov.] Mange, 21 Feb 1949, *F.C. Deighton 4978* (K); [Northern prov.] Yifin, 23 Mar 1964, *Norton & Gladhill 994* (K, WAG0185139); [Northern prov.] Koinadugu distr., SE of Fadugu, 21 May 2014, *X.M. van der Burgt 1854* (K);

Somalia (new record): [Gedo prov.] nr Ganane, Mar 1893, *D. Riva 736* (G, RO);

South Africa: [Gauteng prov.] Pretoria, Apies river, [without date] *ex herb. J. Burtt Davy s.n*. (K); [Gauteng prov.] Pretoria distr., Apies river, [without date] *Zeyher 612* (K); [Western Cape] Clanwillam, Feb [no year] *Zeyher & Ecklon 1818* (P04577109); [Western Cape] Clanvillam, secus fluvium Olifantsrivier ad villam Brackfontein, Jun 1847 *Zeyher 612* (P04577110, P04577124); Cape of Good Hope, 1847, *C.-L. Zeyher 612* (FR-0132679, G, LE, P04577110); Northern Cape, Henkries, 30 Nov 1897, *B. Schlechter s.n*. (BR0000018269291, G, LE, L1693128, P04576623, WAG1103197, Z-000092196); [Limpopo prov.] Musina, 2000 ft, [without date] *F.A. Rogers 22240* (G); [KwaZulu-Natal prov.] Durban, Sep 1904, *M. Wood s.n*. (L1693126); [Limpopo province] Messina [Musina], Nov 1917, *Moss & Rogers 57* (BM, K); [Mpumalanga prov.] Klipfontein, Nov 1937, *J.P.H. Acocks 5145* (K); [Transvaal prov.] Zoutpansberg, Kruger Nat. Park, 800 ft, 2 Nov 1948, *O’Dyer 4630* (K); [Northern Cape] Lokenburg, 2100 ft, 16 Mar 1957, *J.P.H.Acocks 19196* (K, M, PRE0403696, PRE0403696-2); [Northern Cape prov.] Kimberley, Rooipoort, 3450 ft, 1 Dec 1958, *O.A. Leistner 1210* (K); Cape Prov., [Northern Cape prov.] Warrenton, [without date] *J.P.H.Acocks 15916* (K); [KwaZulu-Natal prov.] Ubombo Distr., Mkuzi Gane reserve, 9 Dec 1959, *C.J.Ward 3353* (K, M); Transvaal prov., Kruger NP, 350 m a.s.l., 27 Jan 1982, *J. Lambinon & M. Reekmans 82* (BR0000018269246, MSB-129442, PRE0626149); [Limpopo prov.] Giyani distr., 18 Oct 1988, *S. Venter 13049* (PRE0810982);

South Sudan (new record): [Equatoria Region] Zande, 6 Mar 1940, *J.W.AnonymousWyld 781* (BM);

Tanzania (new records): [Ruvuma region] 7 km W of Songea, 990 m, 18 Jan 1956, *E. Milne-Redhead & P. Taylor 8355* (BR0000018269017, K); [Rukwa region] Sundu Lake, 1500 m, 13 Dec 1958, *H.M. Richards 10316* (K); Kigoma region, Mugombasi, 1 Sep 1959, *R.M. Harley 9495* (K); [Kagera region] Ngara, 4500 ft, 6 Sep 1960, *R. Tanner 5126* (BR0000017454308, K, WAG1103204); [Singida region] Ruaha NP, Great Ruaha river, 840 m, 19 Jan 1966, *M. Richards 21007* (K); Arusha region, Ngurdoto Crater NP, 22 Mar 1966, *P.J. Greenway & Kanuri 12474* (BR0000017454285, K); Arusha region, Arusha NP, 1524 m, Nov 1969, *M. Richards 24683* (K, M); [Singida region] Ruaha NP, 820 m, 12 Oct 1970, *A. Bjørnstad 601* (K); [Arusha region] Ngorongoro Conservation area, 2200 m, 30 Dec 1988, *T. Pocs & S. Chuwa 88308* (K); Mbeya region, 19 Dec 1989, *J. Lovett et al. 3775* (L0717072, P04576671); [Kilimanjaro region] Kilimanjaro, 1220 m, 2 May 1994, *J.M. Grimshaw s.n*. (K);

Uganda (new record): [Western region] Kiryadongo, 3200 ft, Mar 1943, *Anonymous 1338* (K);

Zambia (new records): [North-Western prov.] Mwinilunga distr., 6 Nov 1937, *E. Milne-Redhead 3115* (BR0000017454414, K); [Southern prov.] Mapanza, 20 Sep 1953, *E.A. Robinson 324* (BR0000018269109, K); [Copperbelt prov.] Ndola, 23 Jun 1955, *D.B. Fanshawe 2343* (B101143625, BR0000018269604, K); [Lusaka prov.] S of Lusaka, Kafue river, 3000 ft, 11 Dec 1955, *E.B. Best* 121 (K); [Eastern prov.] Lundazi, Tigone Dam, 1200 m, 19 Nov 1958, *N.K.B. Robson 655* (BM, K); [Southern province] Choma, 25 Oct 1958, *E.A. Robinson 2900* (M); [Eastern prov.] stream beside Chadiza turn-off, Nsadzu to Fort Jameson [Chipata], 900 m, 25 Nov 1958, *N.K.B. Robson 703* (BM, BR0000017454438, K); [Northern prov.] Kali Dambo, [without date], *H.M. Richards 4091* (K); [Central prov.] Broken Hill [Kabwe], 14 Oct 1959, *J.M. Mutimushi 74* (K); [Eastern prov.] Chimutengo/Petauke, 3 Oct 1966, *J.M. Mutimushi 1542* (K); Lusaka prov., Chitendabunga, 1200 m, 13 Nov 1993, *M.Anonymous Bingham 9795* (WAG1103214);

Zimbabwe (new records): Bulawayo, May 1898, *R.F. Rand 331* (BM); Salisbury [Harare], Sep 1919, *Eyles 1806* (K); [Matabeleland North prov.] Victoria Falls, Nov 1933, *A. Meebold 11992* (M); [Matabeleland South prov.] Beitbridge, Limpopo river, 21 Dec 1935, *Smuts & Gillett 3108* (PRE0800058); [North-Western prov.] Salisbury [Harare], Makabusi river, 4500 ft, 24 Oct 1945, *H. Wild 269A* (K); [Matabeleland South prov.] Bulalimamangwe distr., Tegwani, 8 Jan 1946, *F.W.J. McKosh 14297* (K); [Manikaland prov.] nr Mutare, 3500 ft, 29 Dec 1946, *B.S. Fisher 16533* (K); Nuanetsi, 1850 ft, Nov 1955, *R. Davies 1610* (K); Bulawayo, 4800 ft, Dec 1956, *O.B. Miller 4000* (BR0000018269628, K); [Mashonaland West prov.] Urungwe distr., Zwipani Vlei, 30 Nov 1957, *R. Goodier 425* (K); [Matabeleland South prov.] Nuanetsi, 2050 ft, 6 May 1958, *R.B. Drummond 5599* (K); [Matabeleland South prov.] Gwanda Distr., nr Chikwarakwara, 12 May 1958, *R.B. Drummond 5789* (K); [Mashonaland Central prov.] Sipolilo, Angwa river, 3 Jun 1965, *M.Anonymous Bingham 1548* (BR0000018269550); [Midlands prov.] Owelo [Gweru], 4200 ft, 30 Dec 1965, *B.K. Simon 587* (K); [Midlands prov.] Kwekwe distr., Sable Park, 26 Feb 1976, *J.M. Stephens 340* (M, WAG1103229);

##### General distribution.

Africa (Sub-Saharan Africa; North Africa: Egypt [WU!]; Madagascar: G! K!).

#### Glinus lotoides

Taxon classificationPlantaeCaryophyllalesMolluginaceae

L., Sp. Pl. 1: 463 (1753).

6F90EC3C-7BD7-5962-9173-E48B5EEDA911

≡ Mollugo glinus A.Rich., Tent. Fl. Abyss. 1: 48 (1847), nom. illeg. superfl. **Lectotype** (Adamson: 1961: 127): [icon] Alsine lotoides sicula in Boccone (1674: 21, tab. 11, figure B). **Superseded neotype** (Jeffrey 1961: 15): [Italy] Sicily, *Boccone* (OXF, n.v.). **Note.** The protologue of Glinus lotoides L. is based on two main elements, an illustrated treatment of Alsine lotoides sicula from Sicily (Boccone 1674: 21, tab. 11, figure B) and another illustrated treatment of Portulaca baetica, luteo flore, *spuria aquatica* from Spain (Barrelier 1714a: 47, 1714b: figure 336). Linnaeus had not seen any specimen of the species prior to the publication of the protologue (Adamson 1961), in particular the collection of P. Boccone at OXF (Jarvis 2007). The herbarium collections of J. Barrelier are no longer extant (cf. Morton 1970) and had seemingly never been consulted by any botanist because all Barrelier’s legacy but drawings was destroyed by fire after his death (Barrelier 1714a). There were two attempts to lectotypify the name Glinus lotoides. Jeffrey (1961) designated a specimen collected by Boccone and kept at OXF; although this specimen is associated with the illustration in Boccone (1674), it was not examined by Linnaeus and therefore is not part of the original material. Jeffrey’s lectotypification is in effect neotypification. Adamson (1961) designated the illustration in Boccone (1674) as lectotype. This lectotypification is correct and supersedes the neotype designated by Jeffrey.
= Holosteum hirsutum L., Sp. Pl. 1: 88 (1753). **Holotype**: India. Ex Malabariae [Malabar Coast], Hb. *Van Royen 899* (L, n.v., after van Steenis 1966).
= Glinus dictamnoides Burm.f., Fl. Ind.: 113 (1768). **Lectotype** (designated here): [icon] Plate 356, figure 6 in Plukenet (1705). **Note.** The name G. dictamnoides was erroneously attributed by Fenzl to Linnaeus (1771); however, the latter clearly refers to Burman’s “Flora Indica” where G. dictamnoides was described (Burman 1768). Burman (1768) cited no specimens in the protologue. Both Burman (1768) and Linnaeus (1771) cited Plukenet (1705) who depicted this plant in the Plate 356, figure 6. This image shows a hairy shoot with the rounded leaves in whorls and almost sessile verticillate flowers. The obovate vs. orbicular leaf shape was the main character known to Burman to distinguish G. lotoides and G. dictamnoides in situ (Burman 1768; see also a drawing of G. lotoides in the table 36, figure 1). The orbicular and greyish-green leaves of G. dictamnoides were similar to those of Dictamnus creticus Garsault (≡ Origanum dictamnus L.), and such plants were found in Madras [now Chennai], India: “dictamni cretici facie, maderaspatana”. Based on the protologue of G. dictamnoides, Merrill (1921) synonymized this species name with G. lotoides. In agreement with Merrill’s opinion, we designate the cited illustration as lectotype and retain Burman’s name in the synonymy of G. lotoides. The specimens seen from India usually have rounder leaves with ± scattered pubescence and shorter perianth (usually 5.5 mm long in fruiting) compared to the European populations, which have obovate and usually hirsute leaves and longer perianth reaching 6.5–8.5 mm in length. These characters were presumably the main argument to consider the Indian plants as G. lotoides subsp. hirtus (Almeida 1998) based on the name Mollugo hirta described from South Africa but erroneously applied to Indian plants (Clarke 1879). Mollugo hirta represents the plants with different characters (see also notes under G. hirtus). The African plants corresponding with G. dictamnoides are present in eastern and southern parts of the continent. It should be noted that the density of pubescence is very diverse in African plants, and those growing in a humid climate (e.g., Nigeria, Cameroon) usually have green leaves with scattered hairs. The intermediate forms in leaf shape and pubescence degree were frequently seen in the herbarium collections. In light of our molecular studies showing a mixed position of G. lotoides and G. dictamnoides, and scarce morphological differences between them, we prefer to synonymize G. dictamnoides with G. lotoides.
= Tryphera prostrata Blume, Bijdr. Fl. Ned. Ind. 11: 549 (1826). **Note.** Described from two localities on the Island of Java, Indonesia: Pamanukan and Cheribon [Cirebon]. The authentic specimens are not traced, but the description of the genus and species (Blume 1826) indicates the identity of T. prostrata to G. lotoides.
= Pharnaceum pentagynum Roxb., Fl. Indica 2: 103 (1832). **Lectotype** (designated here): [without location, year and collector] “Hb. Roxb.” (K000641797!). **Note.** Another authentic specimen was found at BR: “Herb. Roxburghii” (BR0000005227204).
= Glinus lotoides var. virens Fenzl, Ann. Wiener Mus. Naturgesch. 1: 358 (1836).
≡ Mollugo glinus var. virens (Fenzl) Oliv., Fl. Trop. Afr. 2: 590 (1871). **Lectotype** (Adamson 1961): South Africa. At the junction of Fish and Orange rivers, left side of the Orange River (ca. -28.1, 17.175): [“Garip, bei Verleptpram, am Ufer des Flusses und in der steinigen Niederung, unter 500 Fuss”, 17 Sep 1830,] *J.F. Drège* (K000232021; isolectotypes HAL0117929, HBG516708, K000232020, S05-5102, S05-5103). **Note.** In the type citation, the locality information is added from Drège (1843: 92) and the collection date is complemented from Gunn and Codd (1981). When establishing G. lotoides var. virens, Fenzl (1836) cited several validly published plant names as synonyms (G. dictamnoides, Pharnaceum pentagonum, Physa madagascariensis), and also illustrations and specimens. Since this new name was at the rank of variety and the synonyms were at the rank of species, and Fenzl provided his own description and used his own original material, his variety does not need to be treated as based on any of the synonyms included. Indeed, Adamson (1961) designated a separate type for this varietal name, a specimen collected by Drège in South Africa and cited by Fenzl (1836) in the protologue. This lectotypification is technically correct and should therefore stand. Fenzl (1836) established his variety for the plants that are less villose (or glabrescent at maturity) than the type variety of G. lotoides, with the perianth 5 mm long, and without petaloids. The distribution area of this new variety was circumscribed as East India, Timor, Arabia, Madagascar, and South Africa (Fenzl 1836). This variety was accepted by the later authors, and its range was widened to include many regions of tropical Africa (Oliver 1871 sub Mollugo glinus var. virens; Adamson 1961; Gonçalves 1970, 1978; Figueiredo and Smith 2008; Klaassen and Kwembeya 2013). However, many sheets of similar looking species (G. hirtus and G. zambesiacus) were previously identified as G. lotoides var. virens, and this name was therefore used erroneously in many treatments.
= Glinus micranthus Boiss., Diagn. Pl. Orient., ser. 1, 10: 11 (1849). **Holotype**: [Lebanon] Rascheya [Rachaiya], in ruderatis, May–July 1846, E. Boissier s.n. (G00506579!) located at G-BOIS!

##### Description.

**Figure 19.**
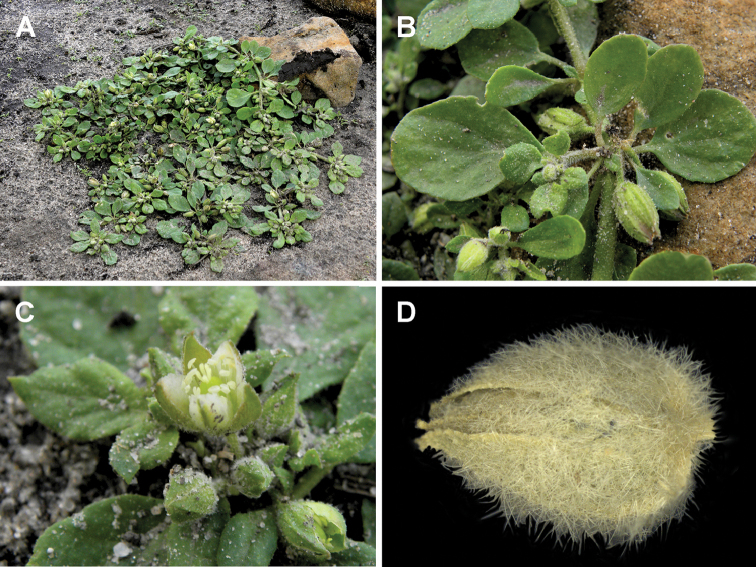
*Glinus lotoides***A** an overview of the plant **B** close-up of the leaves and buds **C** close-up of the flower **D** closed anthocarp. Photographer – Ridha El Mokni (Sidi Mechrig – Sejnane, Bizerta Governorate, Tunisia, 23 Oct 2015).

(Fig. 19). Annual, stellate pubescent. Stems prostrate or ascending, sometimes reaching 2 m in length, but usually shorter (up to 70 cm), prickles absent. Leaves grey or light green, usually moderately pubescent, sometimes bicolored (light green adaxially and grayish-green abaxially), entire, crisp or denticulate, obovate, broadly obovate or almost roundish; petioles 2–10(15) mm, blades (10)15–60 × 4–20(25) mm, mucronulate. Flowers in clusters of 4–12, distant; sessile or with pedicels up to 15 mm (in fruiting the pedicels, if present, may be up to 20 mm long); white or yellowish, sometimes rusty inside and white abaxially, mid-vein green; flower buds and anthocarp ovoid. Perianth segments (tepals) oblong or ovate, entire or sometimes denticulate, in flowering 5.0–6.5 mm long, in fruiting 7.0–8.5 mm long, with a mucro 0.5–1.0 mm long; white petals (1–3) sometimes present (usually much shorter than tepals). Stamens (5)10–16[∞], obdiplostemonous or alternisepalous, outer stamen series (petaloids) sterile, terminating with 1–3 teeth; anthers 0.7–1.0 mm long. Stigmas (3)5, 0.9–1.5 mm long (sometimes a style is present up to 1.0 mm long). Seeds 0.45–0.6 × 0.40–0.55 mm, dark red or almost black, colliculate and without longitudinal striae; aril hood easily visible, 0.20–0.30 mm long.

##### Note.

The five-staminate and three-carpellate individuals of *G. lotoides* are considered here as abnormal forms that seem to be very rare. We assume that such specimens investigated earlier in relation to the floral formulae by Müller (1908) rather belong to *G. hirtus*.

**Figure 20.**
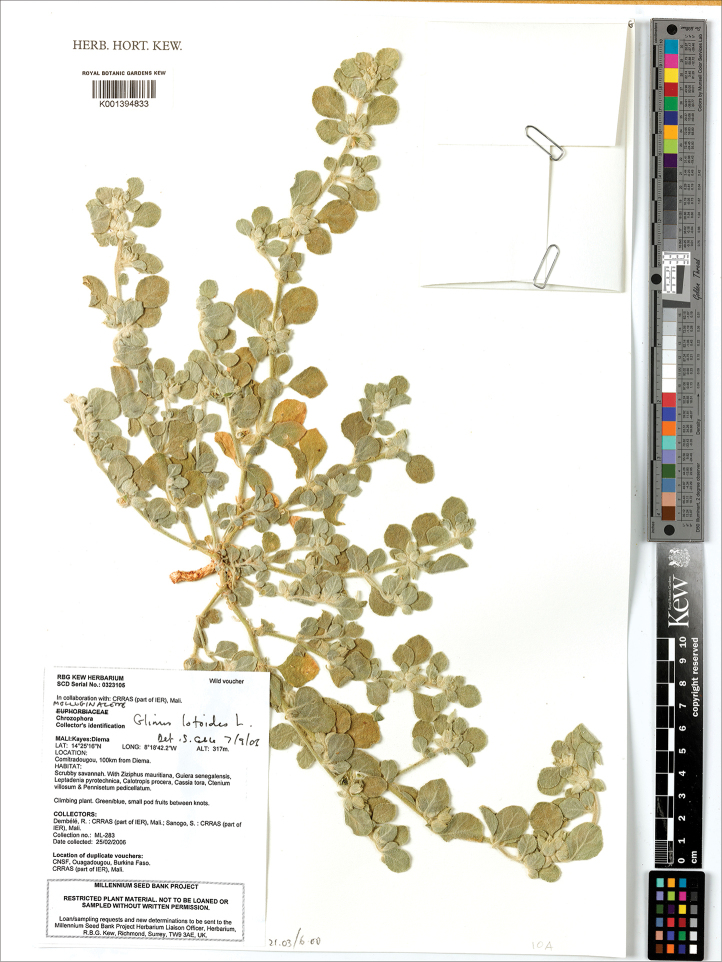
A herbarium specimen of *Glinus lotoides* (Kayes region, Comitradougou, 100 km from Diéma, 14°25'N, 8°18'W, alt. 317 m, 25 Feb 2006, *R. Dembele et al. ML-283*, K001394833). Copyright of the Board of Trustees of the Royal Botanic Gardens, Kew.

The plants with green or glabrescent leaves are more common in the wet climate (e.g., West Africa), and the densely pubescent populations are most frequent in drier conditions (Fig. 20).

##### Habitat.

Sandy sites in river valleys, limestone, clayey and rocky places, seasonally inundated areas; at altitudes of up to 2000 m. This species prefers semiarid regions and only limited number of specimens from West Equatorial Africa are present in the collections.

##### Distribution

**Figure 21.**
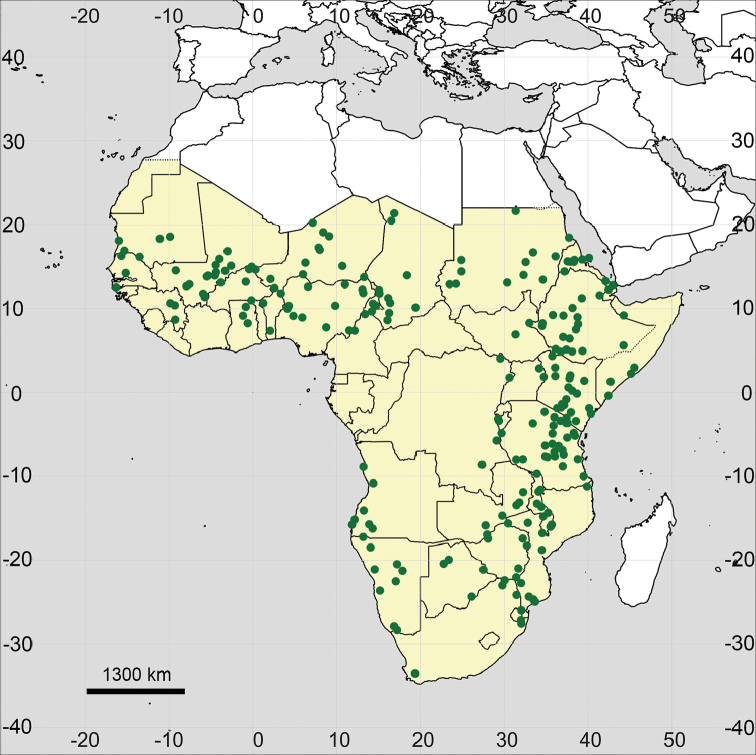
Distribution map of *Glinus lotoides* in Sub-Saharan Africa (colored in yellow).

(Fig. 21). Angola: [Luanda prov.] Barra do Bengo, Jan 1853, *F. Welwitsch 2415* (BM); Luanda distr., between 8 and 9°S, Alto das Cruzes, Sep 1857, *F. Welwitsch 2412* (BM, K, LE, M, P04576620); [Namibe prov.] Mossamedes, Cabo Negro, Sep 1859, *F. Welwitsch 2416* (BM, K); [Namibe prov.] Mossamedes, Cavalheiros, Jul 1860, *F. Welwitsch 2413* (BM); [Namibe prov.] Bero and Giraul rivers, Jun 1900, *J. Gossweiler 72* (COI00070553); Luanda, 1903, *J. Gossweiler 223* (BM, LISC031995, P04576560); [Cunene prov.] nr Cahama, 18 May 1909, *H.H.W.Pearson 2545 & 2548* (BM, BOL217405, K); [Namibe prov.] Mossamedes, Rio Croca, 3 Jun 1937, *L.W. Carrisso & F. Sousa 242* (BM, LISC031994); [Namibe prov.] Porto Alexandre, 15 Sep 1955, *E.J. Mendes 79* (BM, LISC031997); [Namibe prov.] Mossamedes, Camucuio, 14 Oct 1955, *E.J. Mendes 410* (LISC031998, M); Cuanza Sul prov., Foz do Rio Cuvo, 4 Mar 1967, *J.B. Teixeira et al. 11054* (LISC032003, WAG0104171); [Huila prov.] Chiange, 4 Dec 1970, *J.A. de Sousa 86* (K, LISC032011);

Benin: Zou dept. 8 Mar 1970, *L. Aké Assi 11109* (G); [Alibori dept.] Malanville, 13 Nov 1998, *V. Adjakidjè 2593* (WAG0235875); [Alibori dept.] Malanville, 11°52'N, 3°23'E, alt. 270 m, 26 Apr 1999, *P. Houngnon et al. 6507* (BR0000018268560, K, WAG0235862); Atakora dept., Tanguiéta, 20 Jul 2000, *B. Sinsin 3667* (WAG0235863);

Botswana: Ngamiland distr., Kwebe hills, 28 Dec 1897, *E. Lugard 73* (K); [Bakgatla tribal region] Mochudi, 24°10'S, 26°05'E, 1914, *C.C. Harbor 6584* (BM); [Bakgatla tribal region] Mochudi, Jan 1914, *F.A. Rogers 6584* (PRE0403681); [Ngamiland distr.] Maun, Dec 1967, *F.L. Lambrecht 449A* (K); nr Maphaneng, 19°56.75'S, 23°26.2'E, 4 Dec 1977, *P.A. Smith 2115* (K);

Burkina-Faso: [Oudalan prov.] Markoye, 15 Aug 1975, *Anonymous 45121* (P04576576); [Sanmatenga prov.] NE of Pissila, 18 Jun 1987, *J. Lejoly 87/071* (BRLU0026261); [Oudalan prov.] Oursi, 10 Oct 1988, S. *Guinko s.n*. (WAG0042080); Oudalan prov., Tin Akoff, 7 Oct 1998, *S. Kahlheber 1001* (FR-0108631); Oudalan prov., Yomboli, 24 Jul 1999, *J. Müller 82* (FR-0014419); Sourou prov., 50 km W of Tougan on road to Kassoun, 13°10'N, 3°25'W, alt. 276 m, 16 Apr 2002, *L. Sanou 100* (K);

Burundi: Bujumbura, plaine de la Ruzizi km 25, alt. 850 m, 28 Sep 1966, *J. Lewalle 1046* (K); Bujumbura, 800 m, 28 Sep 1971, *J. Lewalle 6145* (BR0000018269406, G, WAG0104170); Bubanza prov., Gihungwe, 800 m, 23 Nov 1974, *M. Reekmans 3952* (BR0000018269437, K, MSB-129441); Bubanza prov., Gihungwe, alt. 850 m, 2 Feb 1975, *M. Reekmans 4296* (K); Bubanza prov., 3 Dec 1977, *M. Reekmans 6677* (K, L0717151, LE, P04576512, PE01696732, WAG0104169);

Cameroon: [Adamawa region] Mayo Lidi, 5 Mar 1933, *H. Lhote 82* (P04576622); [Far North region] Dargala, 30 km ESE of Maroua, 21 Aug 1964, *R. Letouzey 6314* (K, P04576655, WAG0104162); [Far North region] nr Maroua, 400 m, 13 Sep 1964, *W.J.J.O. de Wilde et al. 3240* (BR0000018268638, P04576568, WAG0185134); North region, Garoua, 7 Jun 1975, *P. Wit 3056* (BR0000018268607, PE01942337, WAG0317794); [Far North region] Logone-Birni, 9 Jan 1975, *D. Dulieu 55720* (P04576563);

Chad: Chari-Baguirmi region, Dar Goulla, 28 Mar 1903, *A. Chevalier 7872* (K, P04576585); Tibesti region, Oct 1931, *A. Chevalier s.n*. (P04576654); [Moyen-Chari region] Iro Lake, 12 Jul 1939, *S. de Ganay 127* (P04576532); [Batha region] Mare de Dalato, 31 Aug 1960, *H. Gillet 2375* (P04576666); S of Fort Lamy [N’Djamena], 3 Jan 1963, *W.J.J.O. de Wilde et al. 5188* (BR0000018268676, P04576511, WAG1103207); [Logone Occidental region] nr Moundou, 26 Mar 1963, *B. Descoings 10624* (P04576565); Mayo-Kebbi Ouest region, ne Leré, 30 Mar 1963, *B. Descoings 10727* (P04576567); [Tibesti Ouest Region] nr Zouar, 28 Jan 1965, *H. Scholz 58* (B101143617); [Far North region] Delta of Chari, Djimtilo, 29 Jan 1968, *J. Léonard 4419 & 4420* (BR0000018268614; BR0000018269505, P04576643); [Tandjile region] Satégui, 9 Dec 1968, *Anonymous Fotius 1281* (P04576513); N’Djamena, 28 Jul 1984, *S. Lisowski B-39* (WAG1103213); N’Djamena, 5 Feb 1985, *S. Lisowski 1808* (BR0000018268706);

Djibouti: [Tadjourah Region] Andabba [Lake], 6 Jan 1957, *E. Chedeville 1756* (FT0007026, P04576543);

DR Congo: [Katanga prov.] Kampunda, 3200 ft, 16 Oct 1950, *A.A. Bullock 3430* (B101143607, K); [Haut-Uele prov.] Garamba, 700–800 m, 19 Nov 1952, *Anonymous Troupin 74* (K); [North Kivu prov.] Albert Lake, 5 Mar 1954, *D. van der Ben 1185* (BR0000018269314, K); Katanga, Mitwaba, 21 Aug 1956, *J. Brynaert 520* (K)

Eritrea: [Anseba prov.] Keren, May 1870, *O. Beccari 45* (FI059438, FT0007013); Dahalak [island] Mar 1892, *A. Terracciano 819* (FT0007080); [Southern Red Sea Region] Adarte, 26 Jan 1893, *A. Terracciano & A. Pappi 2741* (FT0007022); [Gash-Barka region] Barea Agordat, 27 Jan 1893, *A. Terracciano 2852* (RO); [Anseba prov.] Bogos, 3 Feb 1893, *A. Terracciano & A. Pappi 2598* (FT0007023); [Gash Barka Region] Mansura, 9 Sep 1905, *A. Pappi 6400* (FT0007016); [Gash-Barka region] Damtai, 30 Jan 1909, *A. Pappi 7840* (FT0007003); [Gash Barka Region] Mansura, 10 Feb 1909, *A. Pappi s.n.* (LE); [Gash-Barka region] Barea Agordat, 23 Feb 1909, *A. Fiori 978* (FT0007029); [Gash-Barka region] Scetel, Dec 1905, *A. Pappi 6747* (RO); [without exact location] 1906, *A. Pappi 6834* (RO); [Gash-Barka region] Mansura [subregion], Beni-Amer, 10 Feb 1909, *A. Pappi s.n.* (LE); Barca [Baraka] river, 30 Mar 1917, *I. Baldrati 230* (FT0007036); Keren, 1300 m, 20 Feb 1919, *A. Fiori 975* (FI059443; FT0007025); [Gash Barka Region] Setit, 4 Mar 1923, *Exped. Corni–Calciati–Bracciani s.n*. (FT0007014); [Northern Red Sea region] Wekiro, 50 ft, 31 Jan 1960, *D.J. Greathead 139* (BM);

Ethiopia: [Amhara region] Magdala [Amba Mariam], 27 Apr 1862, *Steudner 524* (LE); Ogaden [Region] 1891, *L. Robecchi Bricchetti 400* (FT0007010); Addis Ababa, 3 Mar 1916, *L. Buscalioni 1415* (FT0007002); [Oromia region, Borena zone] Melka Guba, 1937, *Anonymous Cutodontis 130* (W); [Southern Nations, Nationalities, and Peoples’ Region] Lago Margherita [Lake Abaya] 21 Jan 1938, *A. Vàtova 1582* (FT0007000); [Southern Nations, Nationalities, and Peoples’ Region] Gondaraba, 29 May 1939, *R. Corradi 7107* (FT007088); [Southern Nations, Nationalities, and Peoples’ Region] Asile, 1 Jul 1939, *R. Corradi 7109A* (FT0007087); [Southern Nations, Nationalities, and Peoples’ Region] Murle Lake, 18 Jul 1939, *R. Corradi 7113* (FT0007085); Somali region, Kelafo, 24 Aug 1960, *Anonymous s.n*. (K); Addis Ababa to Debre Markos, Blue Nile, 9 Feb 1966, *E.F. Gilbert 51* (BR0000018268744, K); [Oromia region, West Shewa zone] Bako, alt. 1650 m, 21 Oct 1969, *C. Parker 198* (K); [Gambela region, Anuak zone] Gambela, 1768 m, 6 Jan 1970, *J.W. Ash 167* (K); Sidamo prov., 1550 m, 2 Mar 1971, *J.J.F.E. de Wilde & M.Anonymous Gilbert 401* (WAG0102276); [Oromia region / Southern Nations, Nationalities, and Peoples’ Region] Ziway Lake, *Feb 1973*, *O. Polunin 11584* (K); [Oromia region, Shewa zone] Meki, 6 May 1976, *M.Anonymous Gilbert & T.Anonymous Jefford 4252* (K); [Oromia region, East Welega zone] between Nekemte and Gimbi, 18 May 1976, *P.C.M. Jansen 6355* (BR0000018268737, FR0104475, FT0007032, G00350481; MBM369175 – image seen! WAG0181906); [Oromia region, Borena zone] 45 km W of Yavello towards Teltele and Konso, 975 m, 15 Nov 2010, *I. Friis et al. 13681* (K, WAG1950295); [Oromia region / Ethiopian Highlands region] Shala Lake, 20 Feb 1979, *M.Anonymous Gilbert & S.B. Gilbert 1173* (K); [Gambela region] N of Abobo, Alwero river, 650 m, 20 Apr 1982, *I. Friis et al. 2476* (K); [Oromia region, Borena zone] 5 km N of Yabelo, 1600 m, 11 Dec 1998, *I. Friis et al. 9418* (K, WAG0322893); [Afar region] 10 km E of Asaita, 400 m, 17 Sep 2001, *I. Friis et al. 10345* (K, WAG0394466);

Ghana: [Upper East region] nr Bawku, 12 Dec 1950, *C.D. Adams & AnonymousK. Akpabla 4290* (K, P04576600, WAG0185140); [Northern region] 53 miles N of Tamale, 20 May 1952, *J.K. Morton 7200* (K, WAG0185141); [Savannah Region] Yapei, 30 Mar 1956, *C.D. Adams 3917* (K); Yeji, 11 Apr 1964, *J.B. Hall 1251* (K);

Guinea: [Kouroussa pref.] Kouroussa, Dec 1900, *M. Pobeguin 605* (K, P04576530); [Nzérékoré Region] Ouamadou, Dec 1917, *J. Berhaut 930* (BR0000018268423); Kankan region, Kankan vill., 23 Apr 1967, *S. Lisowski 61907* (BR0000018268508);

Kenya: Makueni county, Kibwezi, 975 m, without date, *P. Luke 14330* (K); [Turkana county] Lokitaung, 3000 ft, Jan 1932, *A.M. Champion 14* (K); [Eastern prov.] Athi plains, 21 Aug 1938, *P.R.O. Bally 7442* (K); South province, Magadi Road, 3000 ft, 8 Aug 1943, *P.R.O. Bally 2679* (G, K); [Eastern prov.] Athi River Station, 24 Aug 1947, *A. Bogdan 1109* (K); [Marsabit county] 16 miles from Laisamis on road to Marsabit, 2500 ft, 3 Oct 1947, *P.R.O. Bally 5471* (K); [Kajiado county] 1 mile S of Kajiado, 1650 m, 21 Feb 1953, *R.B. Drummond & J.H. Hemsley 1249* (B101143601, BR0000018268799, FT0007096, K); [Lamu county] Witu, [without date] *Thomas 127* (BR0000018269000, G); [Taita-Taveta County] Voi, Sala Gate Rd, 1100 ft, 2 Jan 1967, *J.Anonymous Greenway 12937a* (K); [Turkana county] Ayangyangi swamp, 1900 ft, 4 Sep 1968, *O.M. Mwangangi & D. Gwynne 1235* (BR0000018268829, K); Turkana county, between Lothagam and Kerio delta, 1200 ft, 29 Aug 1968, *O.M. Mwangangi & D. Gwynne 1218* (K); [Turkana county] Ayangyangi swamp, 1°55'N, 36°5'E, 12 Jun 1970, *B. Mathew & M.D. Gwynne 6768* (FT0007099, K); North Eastern prov., 57 km WS of Wajir, 225 m, 6 Mar 1974, *Bally & Carter 16590* (K); Mughwango South swamp, 15 Apr 1972, *J. Ament & F.C. Magogo 9* (K); K4, Tana River county, Kora National Reserve, 18 miles from Kora Res. Camp to Asako, 28 Aug 1983, *J.Anonymous Mutangah 139* (K); Tana River county, Baomo vill., 30 m, 12 Mar 1990, *Q. Luke et al. 141* (K); Embu county, Riakanau, 1125 m, 29 Nov 2000, *S.A.L. Smith et al. 253* (K); Isiolo county, Ngare Mara, 0°30'N, 37°39'E, 945 m, 12 Aug 2005, *C. Obunyali et al. 261* (K); Machakos county, Kitanga, 1°31'S, 37°11'E, 1660 m, 26 Jan 2005, *J.M. Muasya 482* (K); Marsabit county, Log-Logo, 1°59'S, 37°54'E, 520 m, 9 Feb 2005, *J.M. Muasya et al. 505* (K); [Coast prov.] Tana delta, 2°24'S, 40°18'E, 5 m, 4 May 2011, *C. Leauthaud et al. 160* (K);

Malawi: Nsessi, Dec 1887, *L. Scott s.n*. (K); Malawi Lake, 1893–1894, *AnonymousF. Scott Elliot 8403* (K); [Southern region] Mulanje, 16 Nov 1955, *Anonymous Jackson 1761* (BR0000018269536, K); Central region] Ncheu, 29 Sep 1970, *A.J. Salubeni 1491* (K); Central Region, Ntchisi Distr., 1060 m, 20 Jun 1970, *R.K. Brummitt 11583* (K); Northern region, Karonga Distr., Kaporo, 1550 ft, 2 Jan 1974, *J. Pawek 7726* (K); [Southern region] Mangochi Distr., Lake Malawi, 22 Dec 1984, *I.H. Patel et al. 1736* (K); [Northern region] Nkhata Bay Distr., S of Mbuzi Hill, 24 Aug 1984, *A.J. Salubeni & W.Nachamba 3875* (K); [Central region] Salima Distr., Lifidzi breeding centre, 17 Nov 1985, *I.H. Patel & R.B. Kwatha 2898* (K); Southern region, Phalombe, 15°46'47"S, 35°48'44"E, 721 m, 15 Apr 2008, *H.T. Chapama et al. 830* (K);

Mali: [Sikasso region] Zamblara, 16 Jun 1899, *A. Chevalier 991* (P04576672); [Niger Delta region] Djenné, 30 Jun 1899, *A. Chevalier 1129* (P04576675); [Mopti region] Mopti, Niger river, 15 May 1927, *O. Hagerup 72* (K); [Timbuktu region] Timbuktu, Jun 1927, *O. Hagerup 123a* (LE); [Ségou Region] Macina [The Inner Niger Delta], 14 May 1932, *O.B. Lean 14* (K); [Mopti region] Dallah, 19 Jun 1932, *J. Rogeon 388* (P04576669); [Mopti region] Sangha, 14 Jan 1938, *S. de Ganay 73* (P04576552); [Mopti region] Saré Dina, 25 Mar 1952, *J.T. Davey 62* (K); [Mopti Region] Dogo, 7 May 1952, *J.T. Davey 102* (K); [Ségou Region] Kara, 15 Jul 1954, J.*T. Davey 192* (K); [Timbuktu region] nr Timbuktu, 6 Aug 1959, *Anonymous Popov 90* (BM); [nr Bamako] Korofina, 26 Feb 1960, *J. Raynal & A. Raynal 5514* (P04576662); [Sikasso region] Sanzana, 3 Apr 1970, *N. Diarra 590* (P04576520); [Timbuktu region] Ngoro, nr Niafunké, 20 Jan 1991, *A. Raynal-Roques 23030* (P04576559); [Koulikoro Region] Koulikoro, Niger river, 6 May 1993, *R. Ehrlich 463* (B100048300); Kayes region, Comitradougou, 100 km from Diéma, 14°25'N, 8°18'W, alt. 317 m, 25 Feb 2006, *R. Dembele et al. ML-283* (K001394833);

Mauritania: Tagant region, May 1937, *A. Chevalier s.n*. (P04576594); Nouakchott, 13 Feb 1957, *J.Anonymous Adam 12980* (P00695348); [Gorgol region] between Kaédi and Mbout, 14 Jun 1960, *M. Mosnier 590* (P04576667); [Gorgol region] Rinndiao, 16 Oct 1972, *A. Naegele 86* (K); [Trarza region] R’Kiz Lake, 15 Sep 1975, *D. Dupont s.n*. (BR0000018268485); Tagant region, 32 km SE of Tidjikja towards El Khedia, 25 Oct 1987, *M. Carriere et al. 85209* (K);

Mozambique: Gaza prov., 25 km from Vile de João Belo [Xai Xai], 7 Oct 1925, *Anonymous Pedro 218* (K); Sofala prov., Gorongosa NP, 12 Oct 1944, *F.A. Mendonça 2458* (K); Tete prov., Mazowe River, 1000 ft, 22 Sep 1948, *H. Wild 2598* (K); Tete prov., Ilha Micune, 19 Jun 1949, *L.A. Grandvaux Barbosa & F. de Lemos 3187* (K); Gaza prov., Guija, between Aldeia do Guija and Aldeia da Barragem, 15 Nov 1957, *L.A. Grandvaux Barbosa & F. de Lemos 8141* (K); [Gaza prov.] between Chibuto and Canicado, 11 Oct 1957, *L.A. Grandvaux Barbosa & F. de Lemos 8001* (K); [Faza prov.] Sul do Save, Chibuto-Baixo Changana, 24 Aug 1963, *A. Macedo & L. Macuacua 1139* (K); Lorenço Marques [Maputo] prov., Namaacha, 5 Jul 1967, *A. Marques 2043* (WAG1103167); Gaza prov., Caniçado, 23 Aug 1969, *M.F. Correia & A. Marques 1181* (E, WAG1103169); [Sofala prov.] Gorongosa NP, Jul 1970, *K.L. Tinley 1938 & 1941* (BR0000018269178, M, WAG1103228); Gaza prov., Limpopo river, 2 Aug 1973, *M.F. Correia & A. Marques 3156* (WAG1103171); [Tete prov.] Cahora Bassa, 2 Nov 1973, *M.F. Correia & al. 3700* (WAG1103172); [Sofala prov.] Gorongoza NP, 9 Nov 2007, *P. Ballings & B. Wursten 945* (BR581201); Cabo Delgado prov., Ruvuma, 15 Nov 2009, *Q. Luke 13804* (K, P04557299);

Namibia: Waterberg region, Quickborn, 28 Oct 1928, *R.D. Bradfield 301* (K); [Kunene Region] Omuramba to Otamako, 27 Feb 1939, *O. Volk 1201* (M); [Khomas Region] Windhoek, Adodamm, Jan 1947, *Wiss 826* (M); [Erongo region] Brandberg, Ugab river, 8 Aug 1948, *R.Anonymous Stray 2407* (K, PRE0403677); [Erongo region] Kuiseb river, nr Homeb, 11 Oct 1961, *W. Giess 3762* (M); [Kunene region] Kaokoveld, Kunene river, 17°15'S, 12°16"E, 16 Aug 1956, *R. Story 5826* (K, PRE0403678); [Karas Region] Sendlingsdrif, Orange river, Aug 1963, *H. Merxmüller & W. Giess 3259* (M); [Otjosondjupa region] Otjosondu, Omusema river, 1300 m, 10 Mar 1967, *R. Seydel 4495* (B101143620, K, M, PRE0614464); [Karas Region] Lüderitz-South distr., Sendlingsdrif, 21 Sep 1972, *H. Merxmüller & W. Giess 28689* (M, PRE0403676, WAG1103212); [Khomas Region] Windhoek, Farm Regenstein, Dec 1979, *W. Giess 15418* (M);

Niger: [Zinder region] Tasker, [without date] *P. de Fabregues 8834* (P04557298); [Tahoua region] Madaoua, Feb 1932, *A. Chevalier 43716* (PP04576596); [Tilaberi Region] Dolbel, 24 Oct 1962, *J. Jangoux 1214* (BRLU0026263); [Agadez Region] 19°18'N, 7°15'E, 4 Dec 1965, *C.F. Hemming s.n.* (K); [Agadez region] Touaret, 20°20'N, 07°00'E, 10 Dec 1965, *Anonymous Popov 138* (BM); [Diffa region] Bosso, 20 Jun 1966, *J.P. Lebrun 13836* (P04576587); [Agadez region] Iferouane, 19°10'N, 08°30'E, 3000 ft, 22 Jan 1970, *R.A.H. Davies 32* (BM); [Tahoua region] Abalak, 12 Jan 1975, *P. Lavie 870* (P04576521); [Agadez region] Iferouane, 17 Mar 1979, *Dulieu 49673* (P04576588); [Agadez region] Azanyares, 18°9'N, 9°17'E, 19 Mar 1979, *J.E. Newdy ZP64* (K); [Agadez region] Tarouadji, 30 Mar 1980, *E. Schulz s.n.* (B101143619); [Agadez region] 100 km S of Agadez, 23 Nov 1985, *C. Pase 3149* (K); Niamey, 11 Apr 1987, *N. Leman 83* (BR0000018268515); [Tilaberi region] Tapoa zone, West Niger NP, 7 Apr 1996, *H. Breyne 6186* (BR0000018268522, WAG0330951);

Nigeria: [Benue State] Abinsi, 1912, *J.M. Dalziel s.n*. (BM, BR0000018268539, P04576604); [Kwara State] Jebba, 9 Dec 1927, *O. Hagerup 699* (K); [Bauchi State] Bauchi, Feb 1929, *H.V. Lely 144* (K, P04576593); [Sokoto State], Sokoto, 20 Apr 1946, *R.W.J. Keay 15877* (K); [Yobe State] Gashua,8 miles E of Zurmi, bed of Fafara river, 21 Jun 1947, *C.F. Onochie 23365* (K); [Niger State] 10 miles N of Bussa, Shagunu, 10°20'N, 4°25'E, 25 Jul 1965, *C.D.K. Cook 408* (K); [Niger State] Bida distr., at the bank of Niger river, 1 Mar 1968, *B.O. Daramola & A. Binuyo 61946* (K); [Zamfara State] Kaura Namoda area, 22 Jul 1969, *M.Anonymous Latilo 62605* (K); [Taraba State] Sardauna, nr Gashaka, 25 Mar 1970, *J.B. Hall 1577* (K); [Taraba State] Gashaka distr., Gashaka river, alt. 1100 ft, 25 Mar 1970, *Z.O. Gbile & B.O. Daramola 23888* (K); [Niger State] Kainji Lake, 21 Jul 1973, *R. Linnavuori s.n*. (H1209977); [Kwara State] nr Jebba, 15 May 1974, *Latilo et al. 69359* (K, WAG0185138); Borno State, Maiduguri, *E. Denys 740* (BR0000018268553, WAG0104167); Borno State, Gajiganna, 23 Feb 2004, *S. Kahlheber 1082* (FR-0034796);

Senegal: [without exact location] May 1825, *Roger 66* (K); [without exact location and date] *Perrottet s.n*. (RO); [without exact location and date] herb. Maire s.n. (RO); Galam, [without date and collector] *s.n*. (P04577294); [Saint Louis region] Richard Toll [village], 26 Feb 1965, *Anonymous Roberty 16845* (G); Djibelor, 17 May 1982, *C. Vanden Berghen 4987* (BR0000018268478);

Somalia: Somaliland, 75 km S of Hargeisa, near Dofar, 19 Oct 1954, *P.R.O. Bally 10115* (G); [Lower Juba prov.] Jubbada Hoose, Badana Lake, ca. 75 km WSW di Chisimaio, 0°32'S, 41°58'E, 28 Oct 1971, *Anonymous Moggi & R.Bavazzano s.n.* (K); [Shabeellaha Dhexe prov.] Mahaddey Uen [Mahadday Weyne], 13 Mar 1975, *R. Bavazzano 442* (FT0007076); [Lower Shebelle prov.] Afgooye–Merca road, 18 Mar 1975, *R. Bavazzano 486* (FT0007077); Middle Juba prov., Buale to Baardheer, 29 Jun 1978, *S.M.A. Kazmui & al. 777* (WAG0338795); [Middle Shabelle region] 35 km N from Jowhar, 14 Oct 1983, *P. Kuchar 15319* (K); [Middle Shabelle Region] Jowhar, 2007, *A. Sheik A2007* (K);

South Africa: [Western Cape prov.] Cape of Good Hope, [without date] *Drège s.n.* (P04576613); [Limpopo prov.] Messina [Musina], [without date] *F.A. Rogers 19446* (G, Z-000092197); River Limpopo, nr Messina [Musina], 27 May 1927, *R.AnonymousH. Young 14664* (BM); [Zululand] Ubombo, Mkuzi Game Reserve, 100 m, 9 Dec 1959, *C.J. Ward 3352* (K, M, PRE0403692, W); [KwaZulu-Natal prov.] Ingwavuma Distr., 12 May 1965, *Vahrmeyer & Tölken 977* (K, PRE0403694, STU); [KwaZulu-Natal prov.] Ingwavuma, 11 Jul 1974, *H. Furness 97* (E); [Limpopo prov.] Soutpansberg, Limpopo river, 4 Apr 1983, *C. Straub 173* (PRE0653966); Limpopo prov., nr Kruger NP, 350 m, 24 Nov 2000, *N.H.Anonymous Jacobsen 2431* (BR945920);

South Sudan: [Upper Nile State] bank of the Khor Geyni, Pibor river, 4 Jun 1929, *N.D. Simpson 7032* (K); Jonglei State, 8 km S of Maar, alt. 450 m, 18 Feb 1980, *J.M. Lock 80/26* (K);

Sudan: Khartoum, 1860, *Hartmann s.n*. (LE); [River Nile State] Shendi, 25 Oct 1868, *K. Schweinfurth 736* (K); “Kordofan” [former province], Dec 1878, *Exp. Prout 467* (P04577103); Darfur [Region], Jan 1879, *Anonymous 84* (K); [White Nile State] Ed Dueim, 10 Jan 1906, *P.L. de Vilmorin s.n.* (BR0000018268720); Khartoum, 11 Jan 1933, *Anonymous Aylmer 275* (K); Kassala State, Tokar delta, 17–23 Nov 1936, *F.W. Andrews A220* (K); [Gadaref State, south of Hawata town], Huweishrat el Qardud forest, 16 Apr 1961, *J.K. Jackson 4143* (K); Northern State, Wadi Halfa, 29 Sep 1962, *A. Pettet 71* (BR0000018268652, K); Kassala State, Ashkeit, 9 Oct 1962, *T. Ahti 16731* (K); Khartoum, Buni, 30 Jan 1963, *A. Pettet 107* (K, P04577283); [West Darfur State] Jebel Marra, Zalingei, alt. 3400 ft, 7 Feb 1964, *AnonymousE. Wickens 1193* (K); [Central Darfur State] Zalingei, Wadi Aribo, 18 Jan 1965, *W.J.J.O. de Wilde et al. 5348* (WAG1103211); Khartoum, 12 Mar 1965, *W.J.J.O. de Wilde et al. 5836* (BR0000018268713, WAG1103209); North Darfur, Wadi Magrur, 8 Dec 1991, *N. Altmann 347* (B100597466);

Tanzania: Kilimanjaro, 1000 ft, Jan 1894, *Anonymous Volkens 1642* (G); [Rukwa region] Rukwa Lake, [years] 1898–1900, *W. Goetze 1109* (BR0000018268836, G, L1693136, P04577300); [Morogoro region] Kilosa, 28 Dec 1921, *C.F.M. Swynnerton s.n*. (BM); [Dodoma Region] Kondoa distr., 8 Jan 1928, *B.D. Burtt 1869* (K); [Tanga / Manyara regions] Mgera to Kibaya, 1500 m, 23 Aug 1932, *Geilinger 1589* (K); [Dodoma region] Mpwapwa to Gulwe, *H.E. Hornby 390* (K); [Iringa Region] Ilula, 24 Oct 1932, *Geilinger 3292* (K); [Lindi region] 40 km W of Lindi, Lutamba Lake, 3 Oct 1934, *H.J. Schlieben 5427* (BM, BR0000018268959, G, M, P04576499); [Shinyanga / Tabora regions] Shinyanga-Nzega distr., Manyonga river, 3500 ft, Oct 1935, *B.D. Burtt 5232* (BM, BR0000018268997, K); [Iringa region] Idodi, Oct 1936, *E. Ward 29* (K); [Morogoro region] Morogoro distr., Nov 1949, *S.R. Semsei 2868* (BR0000018268867, BR0000018268874, K); [Kilimanjaro region] Mwanga, 18 Aug 1951, *R.E.S. Tanner 393* (K); [Dodoma Region] Mpwapwa, 3000–3500 ft, 3 Nov 1951, *H.E. Hornby 390* (K); [Arusha Region] between Arusha and Moshi, 3500 ft, 23 Sep 1952, *P.R.O. Bally 8325* (K); [Tanga Region] Lushoto distr., 1 May 1953, *R.B. Drummond & J.H. Hemsley 2346* (B101143608, FT0007097, K); Rukwa Region, Rukwa, 2800 ft, 4 Dec 1954, *H.M. Richards 3516* (BR0000018268928, K); [Pwani region] Ruifiji distr., Utete, 2 Dec 1955, *E. Milne-Redhead & P. Taylor 7460* (B101143606, BR0000018269024, K); [Katavi region] Mpanda distr., Kibwezi, 3000 ft, 20 Aug 1958, *J. Newbould & T. Jefford 1687* (K); [Rukwa region] Rukwa, 21 Oct 1959, *H.M. Richards 11517* (K); Dodoma region] Mtera, 19 Apr 1962, *Polhill & Paulo 2077* (B101143604, BR0000018268904, K); [Manyara region] Mbulu distr., Lake Manyara NP, 5 Mar 1964, *P.J. Greenway & Kanuri 11306* (K); Tanga Region, Korogwe distr., 17 Sep 1965, *Semsei 3985* (K); [Manyara region] Tarangire NP, 914 m, 29 Nov 1969, *M. Richards 24827* (K); [Dodoma region] nr Dodoma, 3000 ft, 16 Dec 1969, *W.J. Mapunda & M.D. Raya 1051* (K); [Arusha region] Engaruka valley, 3530 ft, Oct 1970, *H.M. Richards & S. Arasululu 26638* (BR0000018268942, K); [Iringa region] Ruaha NP, 2700 ft, 21 Oct 1970, *H.M. Richards & Arasululu 26297* (K); Arusha Region, Masai distr., Magadini vill., 700 m, 8 Aug 1974, *B. Mhoro & I. Backeus 2028* (K); [Arusha region] Ngorongoro Conservation area, 1 Oct 1977, *J. Raynal 19326* (BR0000018268881, K, P04576664); Morogoro region, Mikumi NP, 500 m, 16 Oct 1986, *A. Borhidi et al. 86150* (K) ; [Kigoma region] Kigoma distr., 22 Aug 1988, *anonymous 1687* (K); [Kilimanjaro region] between Moshi and Himo, 920 m, 3 Feb 1996, *A. Hemp 945* (P000869013);

**Note.** Plants of both specimens of *Glinus lotoides* and *G. oppositifolius* are present on the same herbarium sheet (Tanzania, *Polhill & Paulo 2077*, K).

Uganda: [Nakapiripirit distr.] Kakamongole, at base of Mt Debasien, Jan 1936, *W.J. Eggeling 2607* (BR0000018268768, K); Karamoja [sub-region], Kidepo river, 3400 ft, Apr 1960, *J. Wilson 986* (BR0000018268775, K);

Zambia: Lunsemfwa river, 24 Apr 1929, *J. Burtt Davy 897* (K); [Southern prov.] Makambola Farm, Mazabuka, 3300 ft, 28 Apr 1931, *C.F. Trapnell 345* (K); [Eastern prov.] Nsefu Game Camp, 750 m, 15 Oct 1958, *N.K.B. Robson 138* (K); [Eastern region] Luangwa valley, 13°S, 32°E, 2000 ft, 29 Nov 1957, *Stewart 70* (K); [Eastern prov.] Luangwa Game Reserve, Chilongozi Pontoon, 600 m, 11 Oct 1960, *H.M. Richards 13333* (K); Zambesi river, Feira [Luangwa], 27 Sep 1962, *A. Angus 3350* (K); [Southern prov.] Mazabuka, 2 Oct 1963, *H.J. van Rensburg 2515* (K);

Zimbabwe: [Mashonaland] East prov., Mutoko, Mkota Reserve, 1600 ft, 1 Oct 1948, *H. Wild 2676* (BR0000018269147, K); [Masvingo prov.] Nuanetsi, nr Malipati, 2 May 1961, *R.B. Drummond & R.O.B. Rutherford-Smith 7676* (K); [Matabeleland North prov.] Siabuwa, 2500 ft, Oct 1955, *R.M.Davies 1385* (K); [Mashonaland East prov.] Mutoko Distr., Nyangombe river, 1600 ft, 1 Oct 1948, *H. Wild 2676* (K); [Masvingo prov.] Chiredzi, 31 May 1971, *J.F. Ngoni 147* (K); [Mashonaland West prov.] Lake Kariba, Kessessee Bay, 483 m, 5 Aug 1983, *P. Denny 1215* (L0717097);

##### General distribution.

Africa (Cape Verde, North African countries, Seychelles, Madagascar, Mauritius); W, S & SE Asia; Australia. The distribution in North America is not confirmed in our study.

#### Glinus oppositifolius

Taxon classificationPlantaeCaryophyllalesMolluginaceae

(L.) Aug.DC., Bull. Herb. Boiss., ser. 2, 1: 559 (1901).

2FAE98EB-426A-57DD-87E4-40052BF6E597

≡ Mollugo oppositifolia L., Sp. Pl. 1: 89 (1753). **Holotype**: Sri Lanka. *P. Hermann* in Herb. Hermann 1: fol. 20 (BM000621295!).

##### Description.

Annual, branched from the base, with numerous prostrate stems often forming mats up to 1 m in diameter; young parts of the stems covered with simple crispate hairs usually arranged along one line; prickles absent or unnoticeable with the naked eye. Leaves rosulate, short-lived, and cauline, green, glabrous or puberulent, shortly petiolate (petioles 3–10 mm long), entire or coarsely denticulate, oblong, ovate, obovate or narrowly obovate, (10)15–45(55) mm × 3–15(20) mm, apically shortly acuminate, lateral veins neither recessed adaxially nor prominent abaxially. Flower clusters interrupted, consisting of (1)2–10 flowers, rarely more; flowers usually with unequal pedicels 7.0–20.0 mm long, or sometimes (sub)sessile; buds and closed anthocarp of cylindrical shape. Perianth segments in flowering (2.7)3.0–3.5 mm long, in fruiting (3.5)4.0–5.0(5.5) mm long, glabrous or sparsely pubescent, dorsally green or pink with white margins and ventrally white or pinkish, sometimes turning red at senescence; petaloids usually absent. Stamens usually 5, rarely 4 or 6–7; anthers (03.–0.5)0.6–0.8(1.0) mm long. Stigmas 3, 0.3–0.6 mm long. Seeds reddish or brown-red, rarely yellow-brown, 0.30–0.50 × (0.25)0.3–0.4 mm, colliculate or rarely smooth, longitudinal ridges absent.

##### Note.

*Glinus oppositifolius* is sometimes confused with *Gisekia pharnaceoides* L. (Gisekiaceae). The most remarkable characters of *Gisekia* are the leaves and perianth with white striae (formed by cells with raphides), and its capsules are divided into 5–15 mericarps each containing one exarillate seed (Gilbert 1993b). In contrast to *Gisekia*, *Glinus* species do not have easily visible raphides in any part of the plant and their capsules are multi-seeded with arillate seeds.

### Key to the varieties

**Table:** 

1	Flowers (1–10 in a cluster, rarely more) with pedicels 7.0–20.0 mm long	**G. oppositifolius var. oppositifolius**
–	Flowers often sessile or with short pedicels up to 5.0 mm	**2**
2	Upper part of stems ± lanate; leaves up to 15.0 mm long; clusters with less than 10(15) flowers	**G. oppositifolius var. keenanii**
–	Stems glabrous or sparsely hairy; leaves often longer than 15.0 mm; clusters with 10–20 or even more flowers	**G. oppositifolius var. glomeratus**

#### Glinus oppositifolius var. oppositifolius

Taxon classificationPlantaeCaryophyllalesMolluginaceae

5AA8C7FB-4EDF-5AB3-B5B7-F112588EE155

Figs 22 , 23

= Mollugo spergula L., Syst. Nat., ed. 10, 2: 881 (1759).
≡ Pharnaceum mollugo L., Mant. Pl. Altera: 561 (1771), nom. illeg.
≡ Glinus mollugo Fenzl, Ann. Wiener Mus. Naturgesch. 1: 359 (1836), nom. illeg.
≡ Glinus spergula (L.) Steud., Nomencl. Bot., ed. 2, 1: 668 (1840). **Lectotype** (Jeffrey, 1961: 15): [without location, date and collector] Herb. Linnaeus 112.3 (LINN – image seen!)
= Pharnaceum parviflorum Roth, Nov. Pl. Sp.: 186 (1821) ≡ Mollugo parviflora (Roth) Ser. in DC., Prodr. 1: 391 (1824). **Holotype**: India. B. Heyne s.n. (B† destroyed).
= Physa madagascariensis DC., Prodr. 1: 393 (1824). **Type**: Madagascar. A. du Petit-Thouars (P?, not found). **Note.** This plant was originally described by du Petit-Thouars (1806) as a monotypic genus without any species assigned. De Candolle (1824) named the species with a reference to the protologue of the generic name, without any new material involved. The type of Physa madagascariensis is therefore a specimen collected in Madagascar by Petit-Thouars and presumably deposited at P.
= Mollugo denticulata Guill. & Perr., Fl. Seneg. Tent. 1: 45 (1833).
≡ Glinus denticulatus (Guill. & Perr.) Fenzl, Ann. Wiener Mus. Naturgesch. 1: 361 (1836). **Lectotype** (Jeffrey 1961: 15, as holotype): Senegal. 1831, [AnonymousS.] Perrottet s.n. (P04577215! isolectotypes – BM, G00414309!).
= Mollugo subserrata Blanco, Fl. Filip. [F.M. Blanco]: 51 (1837). **Holotype**: Philippines. [without date] *Blanco 385* (BM!).
= Mollugo glinoides A.Rich., Tent. Fl. Abyss. 1: 48 (1847), nom. illeg., non Cambess. (1830). **Type**: Ethiopia. Shire, Quartin-Dillon s.n. (P, n.v., after Jeffrey 1961: 15).
= Mollugo serrulata Sond., Linnaea 23(1): 15 (1850).
≡ Glinus mollugo var. natalensis Sond. in Harvey & Sonder, Fl. Capensis 1: 120 (1860).**Lectotype** (Jeffrey 1961: 15): South Africa. [Kwa-Zulu] [Port of] Natal [Durban], *Gueinzius 138* (S, isolectotype G!). **Note.** The type of Mollugo serrulata was not labelled by Sonder at G or S. In the latter collection it is stored under Mollugo sp. The specimen seen at G is a typical G. oppositifolius with serrulate leaves.
= Mollugo novo-hollandica F.Muell., Trans. Philos. Soc. Victoria 1: 14 (1855). Described from Australia (banks of the Murray River). Type: n.v.
= Wycliffea obovata Ewart & A.H.K.Petrie, Proc. Roy. Soc. Victoria, n.s. 38: 167 (1926).
Described from Wycliffe (Northern Territory) and Stirling Station (surroundings of Perth) (Ewart and Petrie 1926). Type n.v. (MEL?). **Note.** The protologue of this species as well as the drawings (figure 1) completely correspond with G. oppositifolius, although the seed characters of W. obovata were not described.
= Glinus oppositifolius var. parvifolius Hauman, Bull. Jard. Bot. État Bruxelles 19: 446 (1949). **Holotype**: Congo Belge [Dr Congo], Kwango [prov.], July 1913, Vanderyst 1417 (BR000000895568!). **Note.** Plant with closely set and shorter (up to 10 mm) leaves.

##### Habitat.

Sands, river banks or as a weed; altitude up to 1600 m a.s.l. The most common variety in Sub-Saharan Africa. The associated plants found in Botswana (*A. Heath & R. Heath 456*, K): *Cynodon dactylon*, *Sida cordifolia*, *Glinus bainesii*, *G. hirtus*, *Portulaca oleracea*, *Cyperus polystachyos*, *C. compressus*, *C. longus*, *Pseudognaphalium luteo-album* (formerly known as *Helichrysum luteo-album*). Flowers mostly during the main rains (in southern Africa).

**Figure 22.**
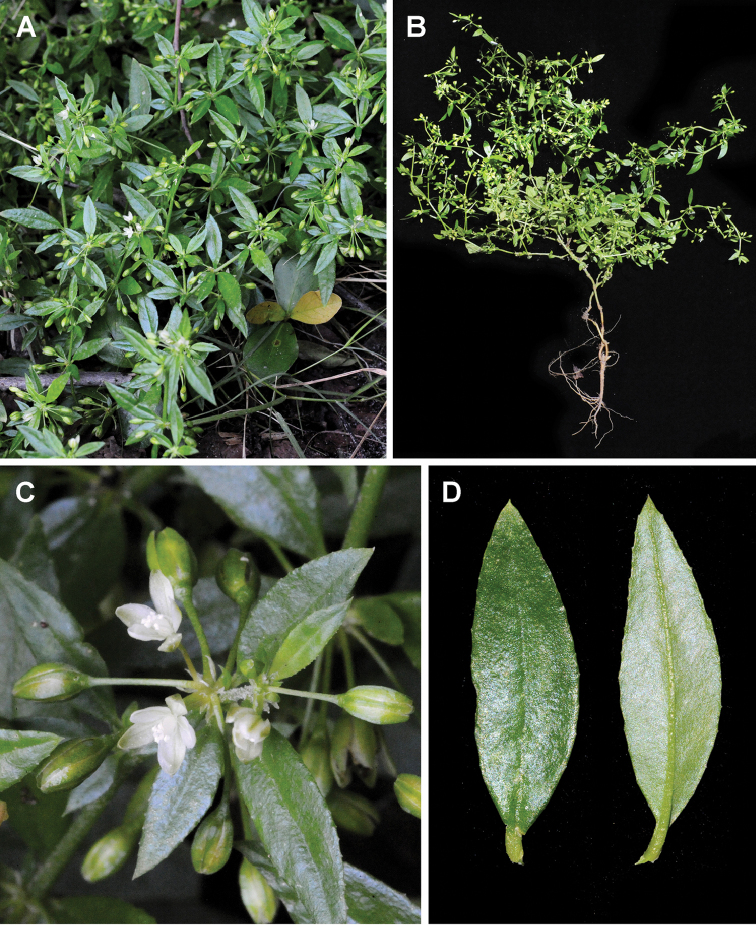
Glinus oppositifolius var. oppositifolius**A, B** general view of the plant **C** close-up of the flowers **D** close-up of the leaves. Photographers – Roger and Alison Heath (Moremi Game Reserve, Ngamiland, Botswana, 07 Feb 2011).

**Figure 23.**
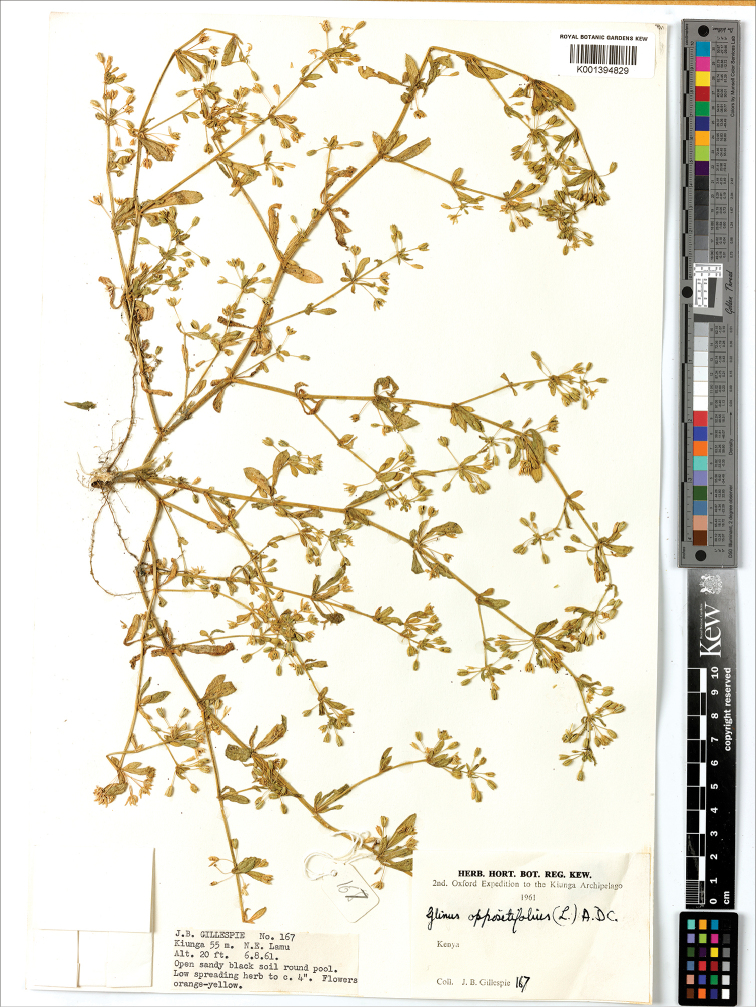
A herbarium specimen of Glinus oppositifolius var. oppositifolius (Kenya, [Lamu county], Kiunga, 55 m NE Lamu, 6 Aug 1961, *J.B. Gillespie 167*, K001394829). Copyright of the Board of Trustees of the Royal Botanic Gardens, Kew.

##### Distribution

**Figure 24.**
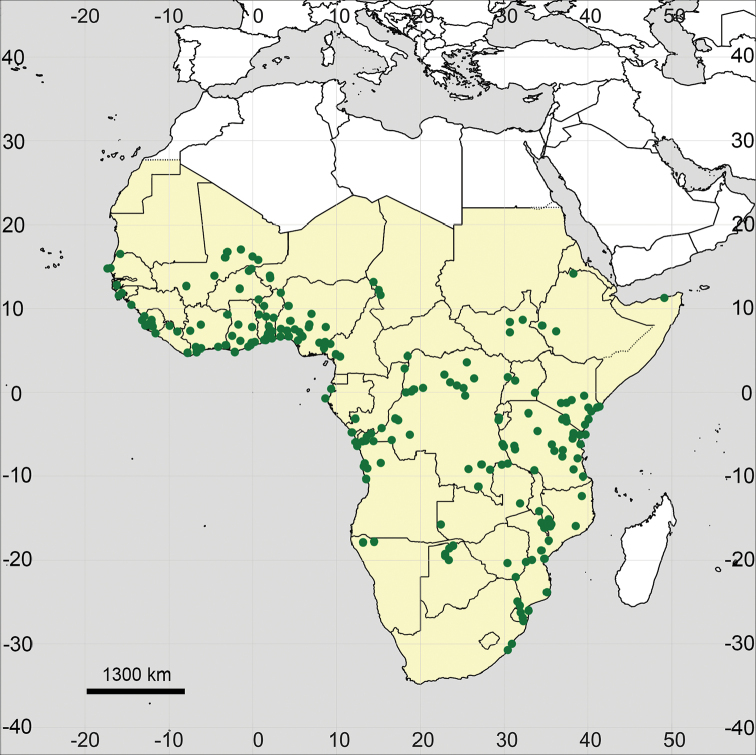
Distribution map of Glinus oppositifolius var. oppositifolius in Sub-Saharan Africa (colored in yellow).

(Fig. 24). Angola: [Bengo prov.] Barra do Bengo distr., Sagoa, Dec 1853, *F. Welwitsch 1265* (BM); [Luanda prov.] between Camama and Calumbo, Jul 1854, *F. Welwitsch 1110* (BM); Luanda distr., between 8 and 9° S, Jan 1858, *F. Welwitsch 2409* (BM, LE); [Bengo prov.] Barra do Dande distr., Dande river, Sep 1858, *F. Welwitsch 1112* (BM); [Zaire prov.] Tombe, 1908, *H.A. Junod 2886* (BM); Luanda, Muceque, Mar 1914, *J. Gossweiler 5880* (COI00070554); Luanda, 1918, *K. Müller 1109* (G); [Kwanza Sul / Bengo provinces] Catemba to Muceque, 60 m, 30 Sep 1935, *J. Gossweiler 10405* (BM); [Bengo prov.] Calemba, 60 m, 30 Sep 1935, *J. Gossweiler 10405* (BM, COI00070555);

Benin: Porto Novo, 6 Mar 1910, *A. Chevalier 23314* (P04577130); Cotonou, 19 Jan 1962, *D. Froment 1141* (BR0000018269932); Ouémé [dept.], Bonou, 12 Mar 1998, *P. Houngnon 5608* (BR0000018269949, K); [Collines dept.] Dassa, 4 Nov 1998, *V. Adjakidjè et al. 2189* (BR0000018269956, K, WAG0095242); Zou [dept.], Djidja, 11 Feb 1999, *J.-P. Essou et al. 1251* (WAG0235854); [Kouffo dept.] Klouékanmé, 15 Feb 1999, *J.-P. Essou et al. 1340* (WAG0235856); Borgou dept.], Tchaourou, 7 Apr 1999, *A. Akoegninou et al. 2258* (WAG0235860); [Alibori dept.] Malanville, 28 Apr 1999, *P. Houngnon et al. 6545* (WAG0235849); Mono dept., Athiémé, 3 Jul 1999, *N. Sokpon et al. 838* (WAG0235853); Zou dept., Cové, 14 Jul 1999, *P. Houngnon 6798* (WAG0235850); [Donga dept.] Bassila, 20 Apr 2000, *P. Houngnon 7634* (WAG0235851); [Collines dept.] Savalou, 9 Jun 2000, *V. Adjakidjè 3595* (WAG0235859); Atakora dept., Natitingou, 28 Jun 2000, *N. Sokpon 1610* (WAG0317484); Mono dept., Comé, 14 Aug 2000, *V. Adjakidjè 3750* (WAG0235874);

Botswana: Northern distr., Mutsoi, 9 Nov 1967, *F.L. Lambrecht 420* (K, PRE0403655); Ngamiland distr., Okavango swamp, 25 Feb 1973, *P.A. Smith 449* (K, PRE0521257); [Central distr.] Boteti river, 20°23'75"S, 24°31'4"E, 15 Feb 1980, *P.A. Smith 3031* (PRE0671278); [North-West distr.] Okavango delta, Maun, 15 May 1984, *P.A. Smith 4443* (BR0000017454513, K); [Ngamiland distr.] Zibalianja lagoon, 18°34.7'S, 23°32.15'E, 957 m, 23 Jan 2004, *A. Heath & R. Heath 456* (BACH, K);

**Figure 25.**
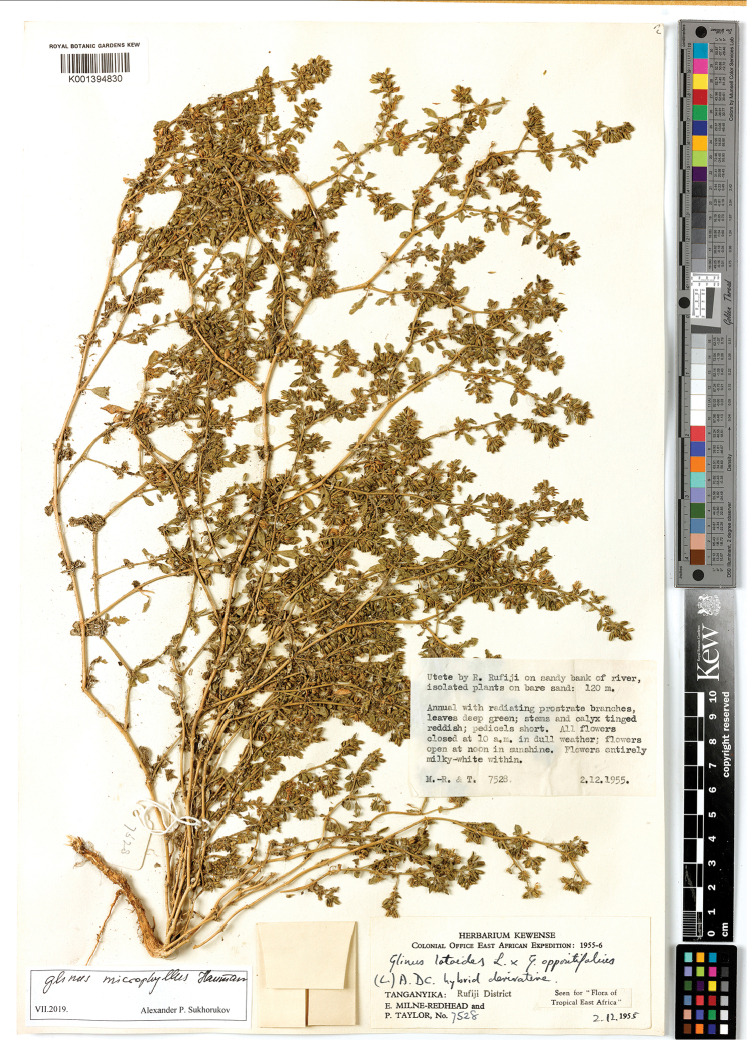
A herbarium specimen of Glinus oppositifolius var. keenanii labeled as *G. microphyllus* (Tanzania, Rufiji distr., 2 Dec 1955, *E. Milne-Redhead & P. Taylor 7528*, K001394830). Copyright of the Board of Trustees of the Royal Botanic Gardens, Kew.

Burkina Faso: Oudalan prov., Oursi, 22 Sep 1995, *S. Kahlheber 145* (FR-0014454); Oudalan prov., Yomboli, 24 Jul 1999, *J. Müller 86* (FR-0014433); Oudalan prov., Darkoye, 8 Aug 1999, *J. Müller 142-c* (FR-0024423); Kompienga prov., 11°09'39"N, 0°37'24"E, 203 m, *M. Schmidt 720* (FR-0021934); Ouagadougou, 8 Jun 1987, *J. Lejoly 87/020* (BRLU0026274);

Burundi: Bujumbura, 800 m, 29 Oct 1968, *J. Lewalle 3076* (BR0000017455862); Bujumbura, 800 m, 28 Sep 1971, *J. Lewalle 6144* (G, WAG0104163); Bubanza prov., Randa, 950 m, 25 Sep 1976, *M. Reekmans 5325* (BR0000017455909, H1235324, K, WAG0185122);

Cameroon: [Southwest region] Mamfe, on the sandbanks by the Cross river, 15 Mar 1953, *C.F.A. Onochie et al. 30889* (K); [Littoral region] nr Masok, Ouem river, 4 Apr 1965, *A.J.M. Leeuwenberg 5378* (BR0000018269987, K, P04577247, WAG0185118); [Littoral prov.] 45 km SE of Yabassi, 18 Jan 1972, *R. Letouzey 11022* (BR0000018270006, P04577207, WAG0185121); [Littoral prov.] Mbombe river, 20 km W of Yabassi, 1 Mar 1976, *R. Letouzey 14779* (BR0000018269994, K, P04577210, WAG0185120);

**Figure 26.**
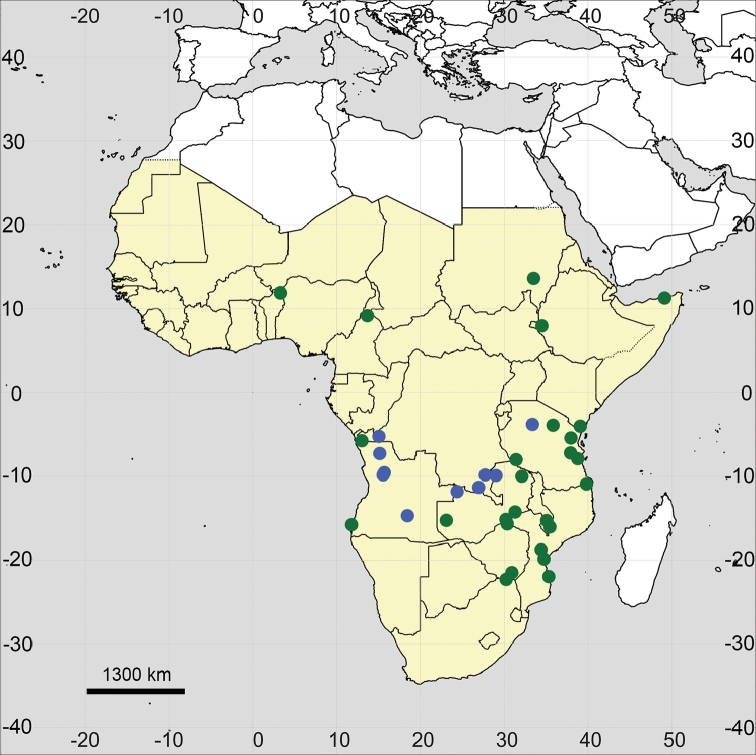
Distribution map of Glinus oppositifolius var. keenanii (green circles) and G. oppositifolius var. glomeratus (blue circles) in Sub-Saharan Africa (colored in yellow).

Chad: [Chari-Baguimi region] Mailao, 1 May 1966, *Stauch 23* (P04577261); [Chari-Baguimi region] between Djimtilo and Lake Chad, 31 Jan 1968, *J. Léonard 4445* (BR0000017456012, G, K, M, P04577268, PE01696734, WAG1103324); [Chari-Baguimi region] Ouazkaga, 27 May 1971, *Anonymous Fotius 1938* (P04577255); Sarh, 7 Jun 1975, *Palayer 605* (P04577265);

DR Congo (selected): [Kwilu prov.] Wombali, Nov 1910, *Vanderyst s.n*. (BR0000013706029); [Province of Équateur] Bokala, *Anonymous 4777* (BM); [Congo Central prov.] Boma, Congo river, 27 Sep 1913, *Bequaert 804* (BR0000017454537); [Kongo Central prov.] Matadi, Nov 1913, *Verschueren 920* (BR0000017455060); [Orientale prov.] Laga, 10 May 1914, *J. Gossweiler 5880* (BM); Leopoldville [Kinshasa], Jul 1915, *L. Achten 17* (BR0000017454667); Bas-Congo [Congo Central prov.], 1921, *J. Claessens 27* (BR0000017454568); [Shopo province] Stanleyville [Kisangani] 25 Jan 1926, *W. Robyns 1418* (K); [Province of Équateur] Bamania, 1930, *J. Lebrun 884* (K); [Congo Central prov.] Boma, 4 Nov 1930, *Vanderyst 27235* (BR0000017454612); [Orientale prov., Tshopo distr.] Yangambi, 6 Jun & 12 Jul 1938, *J. Louis 9711 & 10313* (BM, BRLU0026266, K, P04577193, U1398742); [Province of Équateur] Eala, 5 Aug 1946, *J. Léonard 240* (ALCB012594 – image seen! FT0007107, K, P04577194); [Kongo Central prov.] Vista [Nsiamfumu], 6 Nov 1947, *L. Toussaint 27* (BR0000017454599); Katanga prov., Mitwaba, Nov 1948, *AnonymousF. de Witte 4816* (K); [Tswhopo prov.] Basoko, Jun 1949, *R. Germain 4959* (BR0000017455268); [Haut-Uele prov.] Uele, 3 Mar 1952, *Anonymous Troupin 282* (K, WAG0185123); [Kwango prov.] Popokabaka, 23 Sep 1952, *H. Callens 3707* (BR0000017544780, WAG1103266); [Orientale prov.] Albert Lake, Semliki, 5 Mar 1954, *D. van der Ben 1184* (BR0000017455664, K); [Sud Ubangi prov.] Zongo, 13 Jul 1957, *C. Evrard 2561* (BR0000017455602); Coquille Territory, Indjolo, 3 Oct 1957, *C. Evrard* 2611 (BR0000017455237); [Haut-Katanga prov.] Lupoto, 27 Nov 1957, *A. Schmitz 6031* (BR0000017455763); [Sankuru prov.] [Province of Équateur] Bolomba, 3 Sep 1958, *C. Evrard 4948* (BR0000017455251); [Tshopo prov.] Yangambi, 7 Sep 1959, *P. Bamps 701* (BR0000017455169; WAG0185116); [Bas-Congo prov.] Seke-Banza, Isangila, 22 Sep 1959, *P. Compere 438* (BR00000174554797); [Kongo Central prov.] Kasangulu, Ngombe, 16 Jul 1964, *L. Pauwels 4590* (BR0000017454988); Katanga prov., Kilwa, 900 m a.s.l., 31 Dec 1965, *J.-J. Symoens 11996* (K); [Haut-Lomami prov.] Lualaba river, Bukama, 2 Oct 1970, *M. Lukuesa 740* (BR0000017455756; WAG0185117); Haut-Katanga prov., Kilwa, Moero Lake, 16 Oct 1970, *S. Lisowski 61915* (BR0000017455725); [Tshopo prov.] Kisangani, 5 Nov 1977, *J. Lejoly 2075* (BRLU0026269); [Tshopo prov.] Ubundu, 12 Jul 1981, *Ndjele 427* (BRLU0026273); [Kongo Central prov.] Luozi, 28 Sep 1986, *H. Breyne 5215* (BR0000017454759); [Kwilu prov.] Kikwit, 12 Aug 1991, *B. Masens 1013* (BR0000017455084, K, WAG0405337); [Tshopo prov.] Lokutu, 7 Nov 2004, *Q. Luke et al. 106292* (K); Moenge, Itimbiri river, 360 m, 17 May 2010, *Boyekoli Ebali Congo Exp. 501* (BR0000000571554);

**Figure 27.**
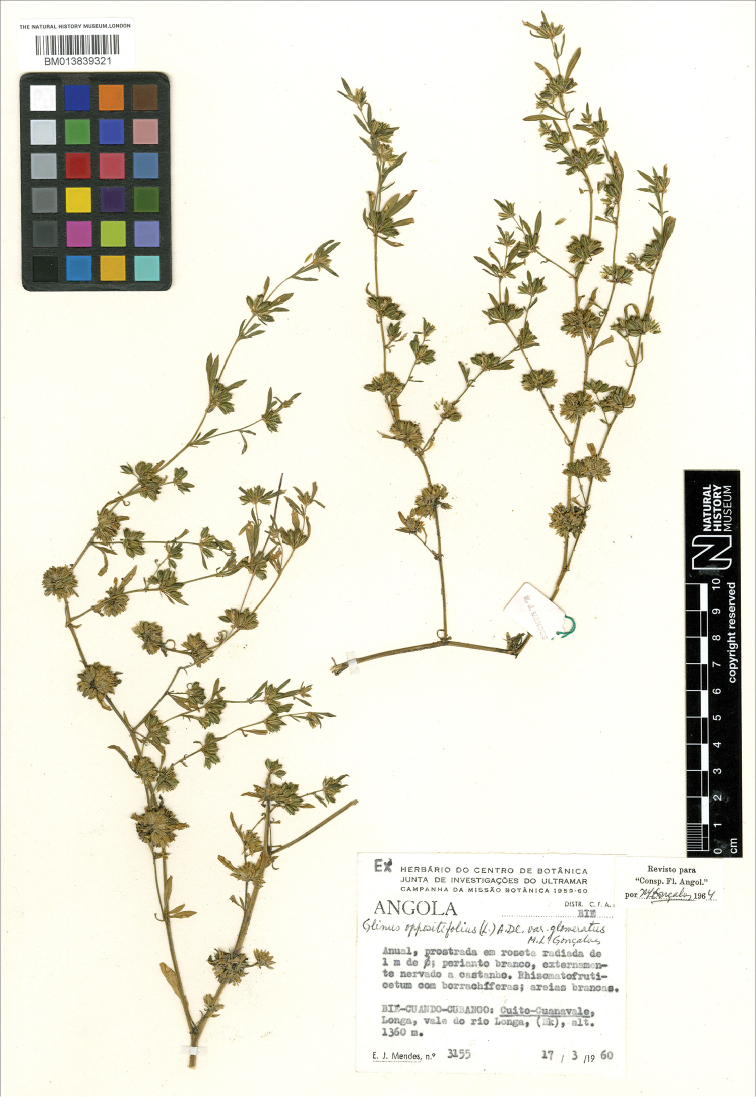
A herbarium specimen of Glinus oppositifolius var. glomeratus (Angola, Cuando-Cubango prov., Longa, Longa river, 1360 m, 17 Mar 1960, *E.J. Mendes 3155*, BM013839321).

Ethiopia: [Southern Nations, Nationalities and Peoples’ region, Keffa zone] Gogeb river, Bonga road, 1300 m, 25 Feb 1966, *W.J.J.O. de Wilde & B.E.E. de Wilde-Duyfjes 10194* (BR0000018270037, K, WAG1103268); [Oromia region] Illubabor zone, Alwero river, Abobo, 630 m, 20 Apr 1982, *I. Friis & al. 2475* (K);

Gabon: [Ogooué-Maritime prov.] Port Gentil, 12 Sep 1968, *F.J. Breteler & R.A. van Raalte 5526* (WAG0185127); N of Libreville, 13 Aug 1992, *J. Dibata 1055* (WAG0070773);

##### Note.

All other specimens identified as *Glinus oppositifolius* indeed belong to *G. hirtus*, *Gisekia pharnaceoides* (Gisekiaceae) and *Polycarpaea* sp. (Caryophyllaceae).

Ghana: Volta river, 27 Mar 1922, *J.M. Dalziel 106* (K, M); [nr Accra] Pokuase, Apr 1931, *F.R. Irvine 1589* (E, K); [Ashanti region] Fumso, 24 Mar 1950, *Anonymous 543* (K); [Western region] Princes Town, 11 Mar 1952, *J.K. Morton 6607* (K, WAG1103299); [Oti region] Kete-Krachi, 19 May 1952, *J.K. Morton 7288* (K); [Greater Accra region] between Weija and Senya Bereku, 20 Mar 1954, *J.K. Morton 204* (WAG0185093); [Eastern region] Mamfe, 6 Apr 1955, *J.K. Morton 320* (K); [Brong-Ahafo region] Jema, 30 Oct 1955, *C.D. Adams 3328* (K); [Greater Accra region] Nungua, 26 Mar 1956, *J.O. Ankrah 20166* (K, M, P04577153, W); [Greater Accra region] Nungua, 25 May 1960, *R. Rose-Innes 31247* (P04577159); [Greater Accra region] Achimota, May 1961, *F.R. Irvine 5474* (K); [Ashanti region] 75 km W of Kumasi, Tano river, 24 Dec 1963, *R.A.A. Oldeman 825* (B101143638, BR0000018269888, K, P04577254, WAG0185089); Brong-Ahafo region, between Nkoranza and Kintampo, 27 Dec 1995, *C.C.H. Jongkind & C.M.J. Nieuwenhuis 2548* (BR0000018269871, WAG0050916);

**Figure 28.**
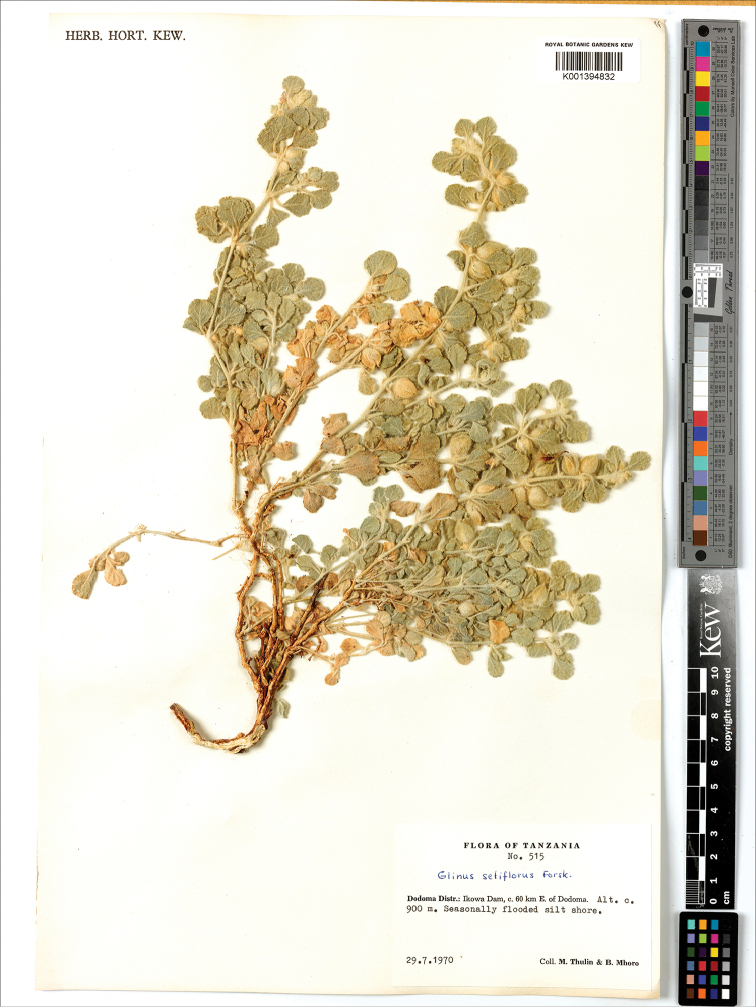
A herbarium specimen of *Glinus setiflorus* (Tanzania, Dodoma region, Ikowa Dam, 60 km E of Dodoma, 900 m, 29 Jul 1970, *M. Thulin & B. Mhoro 515*, K001394832). Copyright of the Board of Trustees of the Royal Botanic Gardens, Kew.

Guinea: [Boke region] Monchon, 6 Feb 1979, *S. Lisowski 51384* (BR0000018269802);

Guinea-Bissau: Antula, 21 Mar 1943, *Anonymous 1499* (B101143640, BR0000018269819, FT0007105, K, M, P04577132, WAG0104159); [Bissagos Islands] Formosa, 26 Apr 1945, *Anonymous 1969* (B101143639, K, M);

Ivory Coast: [San Pédro region] San Pédro, 1900, *M. Thoiré 123* (P04577229); [Woroba distr.] Mankono, 2 Jul 1909, *A. Chevalier 22002* (P04577125); [Bas-Sassandra distr.] 49 km N of Sassandra, nr Dakpadou, 25 Feb 1959, *A.J.M. Leeuwenberg 2858* (BR0000018269864, FT0007110, E, K, M, P04577188, U1398743, WAG0176054); [Montagnes distr.] nr Troya, Cavally river, 8 Mar 1962, *J.J.F.E. de Wilde & A.J.M. Leeuwenberg 3555* (BR0000018269857, K, WAG0185084); [Comoé distr.] Ayamé, 12 May 1965, *L. Aké Assi 8060* (G); [Lacs distr.] Andokoi, 18 Jul 1970, *L. Aké Assi 11277* (G); [Lacs dept.] Betrikan stream, 24 Mar 1971, *J. Audru 3828* (P04577264); Bas-Sassandra distr., Niénokoué, Feb 1983, *N. Stäuble 0861* (G423540); Bas-Sassandra distr., nr Louga, 8 Apr 1973, *J. de Koning 1269* (BR0000018269840); [Zanzan distr.] Bouna, 1 May 1989, *P. Poilecot 3923* (G);

Kenya: [Lamu county] Witu, Dec 1892, *J.W. Gregory s.n*. (BM); Sokoke, 14 Apr 1945, *AnonymousW. Jeffrey 162* (G); [Mombasa county] Kibarani, 4 Apr 1946, *AnonymousM. Jeffrey 515* (K); Kilifi county, 13 Feb 1946, *AnonymousW. Jeffrey 466* (G, K); Machakos county, Kiamkere, 25 Nov 1951, *Kirrika 154* (B101143637, BR0000018270044, K); [Lamu county] Mukunguyu Lake, 5 Nov 1957, *P.J. Greenway & S.P. Rawlins 9449* (FT0007109, K); [Lamu county] Kiunga, 55 m NE Lamu, 6 Aug 1961, *J.B. Gillespie 167* (K001394829); Garissa county, 26 km from Garissa on Hagadera Rd, 0°16'S, 39°47'E, ~230 m, 29 May 1977, *J.B. Gillett 21195* (K); Rare river, 11 Dec 1979, *J.M. Reitsma 413* (BR0000018268782, WAG0318591); Lamu county, Badar Water Pan, 5 Mar 1980, *M.Anonymous Gilbert & P. Kuchar 5892* (K); [Coast prov.] Lamu county, Ras Tenewi, 19 Nov 1988, *P. Luke & S.A. Robertson 1425* (K); Kilifi county, Mangea Hill, 250 m, 27 Dec 1988, *P. Luke 1587* (K); Tana River county, Tana River Primate Reserve, 17 Mar 1990, *P. Luke et al. 524* (K); [Kitui county] Mwingi, 23 Jun 2005, *P. Kirika et al. 542* (K); Lamu county, Bodhei to Basuba, 26 Jul 2006, *L. Festo et al. 2649* (K); [Kilifi county] Malindi, 17 Nov 2010, *S.A. Robertson 7871* (K);

**Figure 29.**
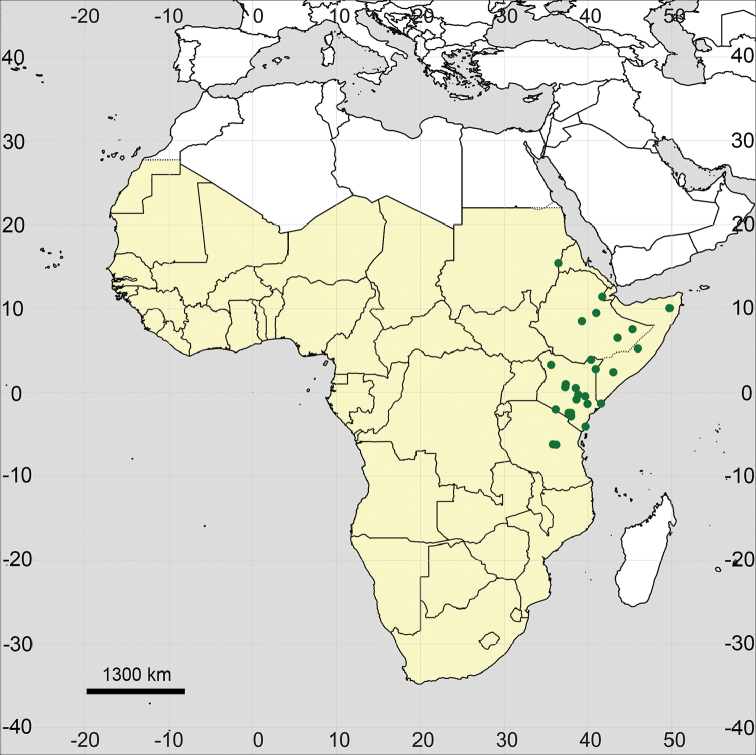
Distribution map of *Glinus setiflorus* in Sub-Saharan Africa (colored in yellow).

Liberia: [Lofa county] Jenneh, 7 Apr 1909, *M. Dinklage 2558* (B101143641); [Nimba county] Ganta, 5 Jun 1973, *A. Jacques-Georges 27822* (BR0000018269833, M, WAG0185083); Maryland county, Cavally river, 8 Apr 2000, *C.C.H. Jongkind & J. Assi-Yapo 4991* (WAG0013107);

Malawi: [Southern region] Chikwawa distr., Lower Mwanza River, 180 m, 3 Oct 1946, *L.J. Brass 17931* (K); [Southern region] Chikwawa distr., Lengwe NP, 300 ft, 15 Dec 1970, *A.Anonymous Hall-Martin 1168* (K, P04577250); [Central region] Dedza Distr., Chipoka, 15 Mar 1972, *A.J. Salubeni 1785* (K); [Southern region] Machinga Distr., Lake Chiuta Harbour, 23 Dec 1984, *I.H. Patel & W. Nachamba 1764* (K); [Southern region] Machinga distr., Dinji vill., 8 Jun 1988, *A.J.Salubeni & I.H. Patel 5203* (K); [Southern region] Zomba distr., Chilwa Lake, Mchisi island, 7 Nov 1986, *A.J. Salubeni & R.B. Kwatha 4814* (K); Southern Region, Mpoto lagoon, Mauzi, Phalombe, 15°39'23"S, 35°49'9"E, 638 m, 7 Dec 2013, *H.T. Vhapama et al. 1088* (K);

Mali: Gao Region, Bamba [without date], *ex herb. A. Chevalier 42330* (P04577220); [Niger Delta region] Djenné, 29 Jun 1899, *A. Chevalier 1193* (P04577226); [Timbuktu Region] Timbuktu, 8 Aug 1927, *O. Hagerup 247* (BR0000017461023, K); [Timbuktu region] Saré-Yamou, 8 May 1932, *ex herb. A. Chevalier 183* (P04577221); Gao region, Bagoundjé, 5 Jul 1936, *M. de Wailly 5079* (P04577225); Gao region, Niger river, 6 Sep 1936, *M. de Wailly 5187* (P04577204); nr Bamako, Sotuba, 13 Oct 1989, *A. Raynal-Roques 22802* (P04577269);

**Figure 30.**
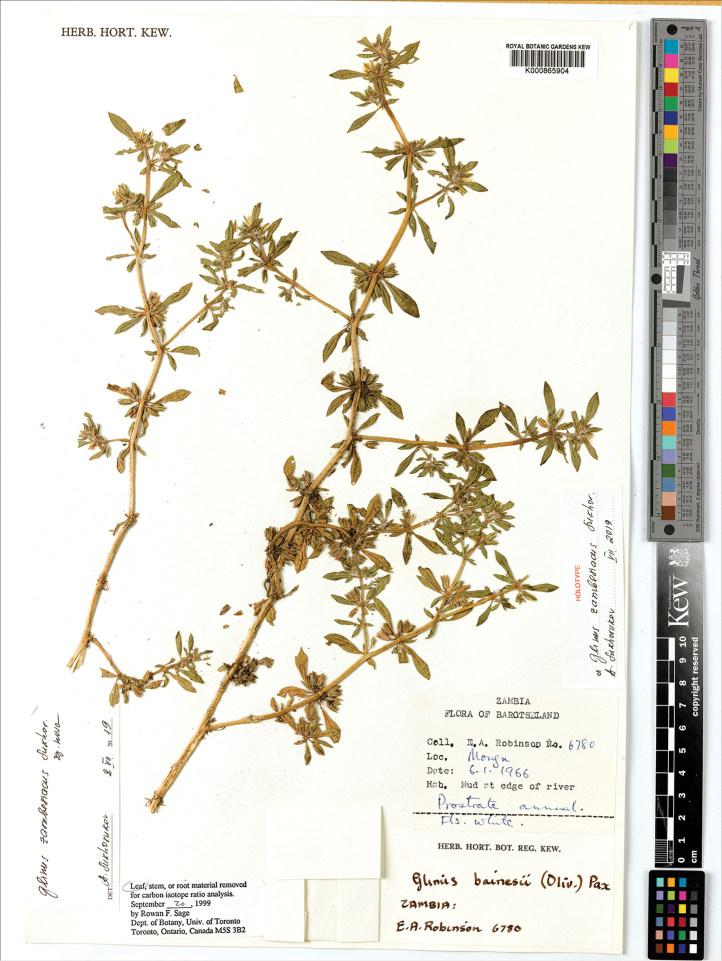
Holotype of *Glinus zambesiacus* Sukhor., sp. nov. (Zambia, Barotseland, [Western province] Mongu, mud at edge of river, 6 January 1966, *Robinson 6780*, K000865904). Copyright of the Board of Trustees of the Royal Botanic Gardens, Kew.

Mauritania: [Trarza Region] Rosso, 11 Oct 1969, *F.N. Hepper 3617* (K, M);

Mozambique: Lorenço Marques [Maputo], Sep 1913, *Anonymous Borle 216* (K); Cabo Delgado prov., 12 Sep 1948, *Barbosa 2097* (K); [Inhambane prov.] Homoine distr., 8 Sep 1948, *M. Myre & M.F. de Carvalho 232* (K); Manica prov., Dombe, 28 Oct 1953, *J. Pedro 4493* (K); [Sofala prov.] Beira, 10 Sep 1962, *A.R.A. Noel 2487* (K); Sofala prov., Gorongosa NP, 3 Feb 1963, *A.R. Torre & J. Paiva 9007* (BR0000017455930, M); Inhaca island, 23 miles E of Lorenzo Marques [Maputo], 200 m, 9 Sep 1964, *A.O.D. Mogg 32038* (K); Lorenço Marques [Maputo] prov., Matola, 21 Jul 1965, *A. Marques 588* (WAG1103275); Zambezia prov., Shire river, Vila Bocage, 13 Dec 1971, *Anonymous Pope & T. Müller 598* (K); Maputo, 10 Oct 1980, *P.A. Schäfer 7280* (BR0000017454452, WAG0155677); Zambezia prov., Mamala, 20 Dec 1996, *A.R. Torre & M.F. Correia 16648* (BR0000017454469);

Namibia: [Kunene region] Ongonga, 14 Jan 1904, *A. Kestilä 30* (H1056354); [Omusati region] Ruacana Falls, 29 Apr 1962, *T.T.Kotze 46* (K, M, PRE0823929);

Niger: Niamey, Sep 1957, *A. Vaillant 891* (K); 100 km N of Niamey, Niger river, 14 May 1968, *C. Geerling & J. Bokdam 2681* (WAG0104161); Niamey, 2 Apr 1987, *N. Leman 81* (BR0000018269963);

Nigeria: Lagos, 26 Mar 1896, *Miller 72* (K); [Cross River State] Oban, 1912, *P.A. Talbot s.n.* (BM); [Benue State] Abinsi, 1912, *J.M.Dalziel s.n*. (BM); [River State] Port Harcourt, Jun 1930, *T.D. Maitland s.n*. (K); [Edo State] Benin, 1934, *W.A. Fairbairn s.n.* (BM); Ondo State, Owo, 31 Mar 1943, *A.P.D. Jones 3087* (K); [Ogun State] Shasha (Omo) forest reserve, Akila, 30 Jan 1947, *C.F. Onochie & V. Emumwen 20687* (K); [Edo State] r[iver] Osse, Iguoriakhi ferry, 29 Jan 1948, *J.P.M. Brenan 8925* (K); [Edo State], Okomu Forest Reserve, 27 Feb 1948, *J.P.M. Brenan 9169* (K); [Ouo State] nr Ibadan, 12 Mar 1950, *R.D. Meikle 1261* (K, P04577150); [Lagos State] Ikeja, 30 Dec 1952, *C.F.A. Onochie 26671* (K); [Oyo State] nr Eruwa, 22 Apr 1958, *D.J. Hamber 430* (K, P04577151); [Cross River State] Ebom, 28 Jun 1955, *R.H. Stone 26* (K); [Oyo State] Gambari Forest Res., SW of Ibadan, 16 Jan 1958, *de Wit 770* (WAG0185105); [Kogi State] Lokoja, 5 Jun 1958, *B.O. Daramola 36930* (K, WAG0185106); [Niger State] Shagunu, 10 km N of Bussa, 31 Jul 1965, *C.D.K. Cook 471* (K, P05307013); [Kogi State], Koton Karfe, 27 Feb 1968, *B.O. Daramola & A. Binuyo 61909* (K); Cross River State, Afunatam, 30 Mar 1972, *J. Lowe 13580* (K); [Cross River State] Ikom, Agbokim waterfalls, 26 Feb 1973, *Latilo & Oguntaya 67685* (K. WAG0185111); [Ekiti State], Igbara Odo, Oruwo stream, 9 Mar 1973, *Olorunfemi & Fagbemi 70748* (K, WAG0185110); [Niger State] Minna, Gurara Waterfalls, 17 May 1973, *Eimunjeze et al. 66411* (WAG0185108); Oyo State, Ibadan, Asejire, 4 May 1974, *Z.O. Gbile 73431* (K, WAG0185112); [Edo State], Iguoriakhi, 16 May 1974, *Eimunjeze & Oguntayo 70213* (K);

Republic of Congo: Niari dept., 29 Nov 1951, *J. Koechlin 1587* (P04576681); Ubangi river, 20 Feb 1963, *de Nere 907* (P04577259); Moutou ya N’Gombé, 12 May 1968, *P. Sita 2036* (P04577256); [Pool Malebo] Mbamu, 14 Oct 1969, *F. Hallé 1641* (P04577257); nr Brazzaville, 26 Aug 1969, *Y. Attims 205* (WAG0034944); nr Brazzaville, 15 Apr 1970, *Y. Attims 442* (BR0000018270020); Kouilou Dept., 20 km SE of Pointe-Noire, Cayo Lake, 22 Mar 2017, *E. Bidault & al. 3022* (BRLU0018798);

**Figure 31.**
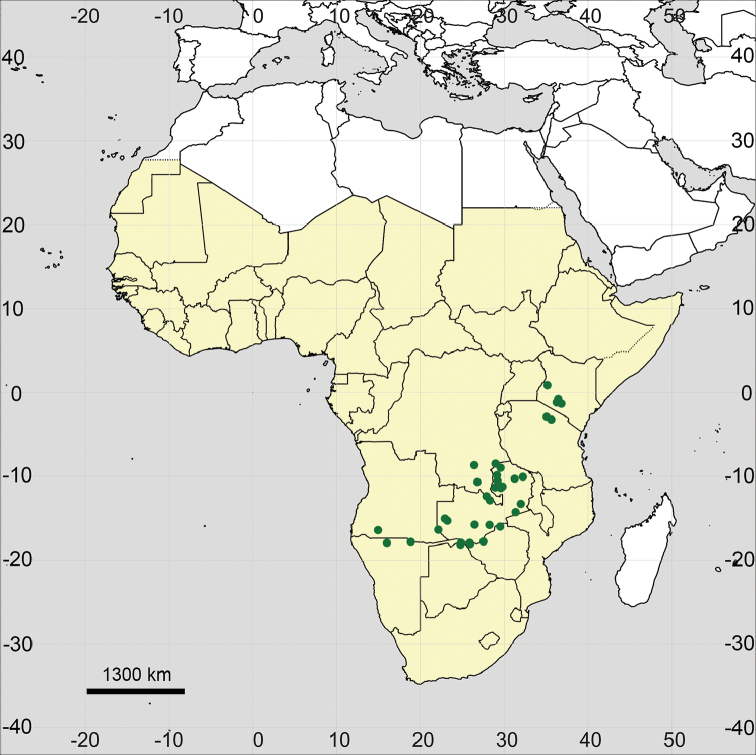
Distribution map of *Glinus zambesiacus* in Sub-Saharan Africa (colored in yellow).

Senegal (selected): “Waalo”, 1831, *Perrottet s.n*. (G); Dakar, 9 Apr 1948, *J.Anonymous Adam 1053* (WAG1103302); [Thiès region]Thiès, May 1948, *R.P. Berhaut 924* (P04577233); [Dakar region] Mbao, 1950–1951, *R.P. Berhaut 2020* (BR0000018269703, P04577235); [Dakar region] Dagoudane-Pikine, 23 Apr 1960, *J. Raynal & A. Raynal 5726* (P04577242); [Thiès region] Kayer, 16 Jun 1961, *J. Raynal & A. Raynal 7067* (P004577267); [Ziguinchor region] Bignona, 31 Mar 1964, *R.P. Berhaut 7267* (BR7000577);

Sierra-Leone: [Northern prov.] nr Madina, 11 Apr 1892, *AnonymousF. Scott Elliot 5561* (BM, K); [Northern prov.] Mokele, Sep 1914, *Anonymous s.n*. (BM); [Southern prov.] Juring, 1 Dec 1926, *F.C. Deighton 297* (K); [Northern prov.] Makump, 3 May 1929, *F.C. Deighton 1707* (BM, K); [Southern prov.] Baoma, 16 Apr 1936, *F.C. Deighton 3166* (K); [Northern prov.] nr Kasanko, 13 May 1951, *P. Adams 225* (K); [Southern prov.] Ngokuma (Kori), 22 Jun 1952, *F.C. Deighton 5845* (K); [Northern prov.] nr Kambia, Magbema, 25 May 1954, *H.D. Jordan 958* (K); [Western Area prov.] Fogbo, 3 Mar 1964, *Morton & Jarr 917* (WAG0185090);

## Conclusions

*Glinus* is a monophyletic genus, presumably originating in tropical Africa, with predominant species diversity in Sub-Saharan Africa. Altogether, we accept six species for Sub-Saharan Africa, and none of them can be considered as locally endemic. Only *G. bainesii* and *G. zambesiacus* are restricted in their distribution to the southern and eastern parts of tropical Africa. A wide range of morphological characters can be used for the identification of *Glinus* species. In total, *Glinus* comprises 8–9 species (*G. bainesii*, *G. hirtus*, *G. lotoides*, *G. oppositifolius*, *G. orygioides*, *G. radiatus*, *G. setiflorus*, *G. zambesiacus*, and probably *G. ononoides*), and further research is needed to clarify the status of the American plants labelled as *Glinus* “*lotoides*”.

## Supplementary Material

XML Treatment for Glinus

XML Treatment for Glinus bainesii

XML Treatment for Glinus hirtus

XML Treatment for Glinus lotoides

XML Treatment for Glinus oppositifolius

XML Treatment for Glinus oppositifolius var. oppositifolius
